# Tackling psychosocial and capital constraints to alleviate poverty

**DOI:** 10.1038/s41586-022-04647-8

**Published:** 2022-04-27

**Authors:** Thomas Bossuroy, Markus Goldstein, Bassirou Karimou, Dean Karlan, Harounan Kazianga, William Parienté, Patrick Premand, Catherine C. Thomas, Christopher Udry, Julia Vaillant, Kelsey A. Wright

**Affiliations:** 1 Social Protection and Jobs Global Practice, World Bank, Washington DC, USA; 2 Africa Gender Innovation Lab, World Bank, Washington DC, USA; 3 UGT/Cellule Filets Sociaux, Niamey, Niger; 4 Kellogg School of Management, Northwestern University, Evanston, IL USA; 5 Innovations for Poverty Action, New York, NY USA; 6 M.I.T. Jameel Poverty Action Lab, Cambridge, MA USA; 7 Department of Economics, Oklahoma State University, Stillwater, OK USA; 8 IRES/LIDAM, UCLouvain, Belgium; 9 DIME, World Bank, Washington DC, USA; 10 Department of Psychology, Stanford University, Stanford, CA USA; 11 Department of Economics, Northwestern University, Evanston, IL USA; 12 Paris School of Economics, Paris, France

**Keywords:** Developing world, Economics, Human behaviour

## Abstract

Many policies attempt to help extremely poor households build sustainable sources of income. Although economic interventions have predominated historically^1,2^, psychosocial support has attracted substantial interest^3–5^, particularly for its potential cost-effectiveness. Recent evidence has shown that multi-faceted ‘graduation’ programmes can succeed in generating sustained changes^6,7^. Here we show that a multi-faceted intervention can open pathways out of extreme poverty by relaxing capital and psychosocial constraints. We conducted a four-arm randomized evaluation among extremely poor female beneficiaries already enrolled in a national cash transfer government programme in Niger. The three treatment arms included group savings promotion, coaching and entrepreneurship training, and then added either a lump-sum cash grant, psychosocial interventions, or both the cash grant and psychosocial interventions. All three arms generated positive effects on economic outcomes and psychosocial well-being, but there were notable differences in the pathways and the timing of effects. Overall, the arms with psychosocial interventions were the most cost-effective, highlighting the value of including well-designed psychosocial components in government-led multi-faceted interventions for the extreme poor.

## Abstract (web summary)

Psychosocial measures improve the cost-effectiveness of multi-faceted interventions against extreme poverty.

## Main

Policies that aim to build sustainable income sources for extremely poor households have historically focused on ‘economic’ interventions such as cash transfers^1,8,9^, grants^10–13^ or microcredit^2,14–22^. Yet the poorest households likely face multiple constraints that limit the ability of any one intervention to provide a pathway out of poverty. Recent evidence has shown that multi-faceted economic-focused programmes can succeed in generating sustained changes^6,7,23–27^. However, psychosocial drivers of poverty have also garnered growing interest^3,5,28–30^, leading to the consideration of psychosocial support in social protection and employment policies. Yet the selection of the most effective components in multi-faceted interventions depends on which combination of constraints drives poverty persistence.

We tested the importance of relaxing capital and psychosocial constraints in alleviating extreme poverty by conducting a four-arm randomized controlled trial (RCT) of a multi-faceted programme implemented by the Government of Niger on top of a poverty-targeted cash transfer programme for women (Table 1). All study groups receive monthly cash transfers. The three treatment arms include a core set of components: savings groups, coaching and entrepreneurship training. A ‘Capital’ arm adds a lump-sum cash grant^6,7^ (and is similar to a graduation programme). A ‘Psychosocial’ arm adds life-skills training and a community sensitization on aspirations and social norms. The ‘Full’ arm adds both the cash grant and the psychosocial interventions. Comparing outcomes in the Full arm with those in the Capital arm provides estimates of the added value of alleviating psychosocial constraints; similarly, comparing outcomes in the Full arm with those in the Psychosocial arm provides estimates of the added value of alleviating capital constraints, inclusive of potential complementarities with the core components.

**Table 1:** Experimental design

		Control	Capital	Psychosocial	Full
	Regular cash transfer programme	+	+	+	+
Core components	Group formation and coaching	−	+	+	+
	Savings groups	−	+	+	+
	Micro-entrepreneurship training	−	+	+	+
	Market access facilitation	−	+	+	+
Psychosocial components	Community sensitization on aspirations and social norms	−	−	+	+
	Life-skills training	−	−	+	+
Cash grant component	Lump-sum cash grant	−	+	−	+
Number of villages (322)		81	80	78	83
Number of sample households (4,712)		1,206	1,191	1,112	1,203

This table describes the components delivered in each of the four experimental arms, along with their corresponding sample sizes.

We contribute to a growing literature on the economic impacts of psychosocial interventions. Whereas interventions targeting beliefs, behaviours, skills and peer relations have shown promising effects on economic behaviour and business outcomes^31–36^, there is mixed evidence on their longer-term impacts on poverty and their added value over economic interventions^4,37,38^. The psychosocial interventions studied here aimed to both build the skills of the beneficiaries and to strengthen instrumental and normative support they receive from their household and community. The psychosocial components thus included life-skills training for beneficiaries as well as innovative, light-touch community programming—a community-wide film screening and discussion targeting social norms and collective aspirations. This design builds on literature around social psychological interventions, sociocultural norms and socio-emotional skills^39–42^.

Further, early graduation studies found limited impacts on women’s empowerment^6,7^, although stronger effects were documented when broader measures were considered^25^. We analyse how the treatment arms differentially affected two dimensions of women’s empowerment, including those related to social well-being and social capital as well as individual control over earnings and decision making.

We find positive and sustained impacts on economic and psychosocial outcomes from all treatment arms. The Psychosocial and Full arms were the most cost-effective, which suggests that integrating psychosocial components within multi-faceted programmes for households in extreme poverty may be key to maximizing the impact per dollar spent. These results also show that government-led multi-faceted interventions can be effective. This is noteworthy as governments are increasingly interested in integrating multi-faceted programmes in national social protection systems, but their effectiveness may differ from the efficacy of smaller-scale NGO programmes measured in past research^43,44^.

## Consumption and economic outcomes

**Fig. 1:**
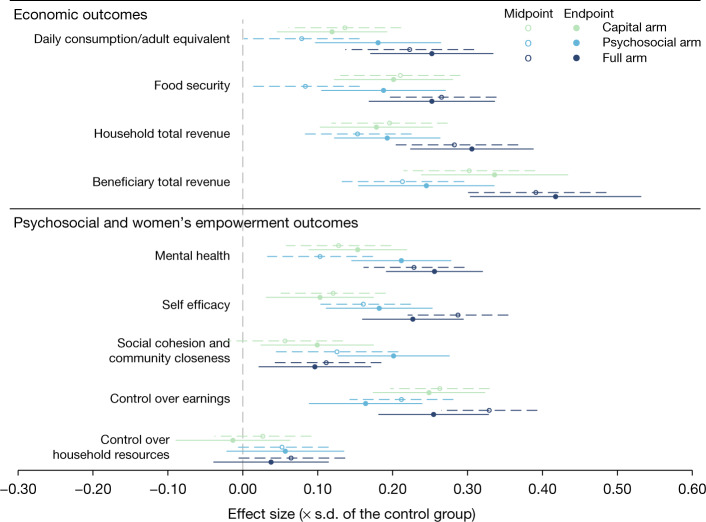
Intent-to-treat estimates for main standardized outcomes. The figure displays treatment effects presented in Table 2. It shows treatment effects on main outcomes, standardized with respect to the control group for ease of interpretation. Results presented are ordinary least squares (OLS) estimates that include controls for randomization strata and, where possible, baseline outcomes. Each circle shows the OLS point estimate and each line the 95% confidence interval corresponding to standard errors clustered at the village level. Dotted lines show results 6 months after intervention (midpoint). Solid lines show results 18 months after intervention (endpoint).

We find positive, consistent and statistically significant impacts of all three arms at the endpoint (a median of 18 months after the intervention) on household consumption and food security (standardized effects in Fig. 1, Table 2; impacts in our pre-specified units in Extended Data Table 1). Daily consumption per adult equivalent increased by 0.12 standard deviations (0.12s.d.) for the Capital arm (standard error = 0.04, *P* = 0.008), 0.18s.d. for the Psychosocial arm (standard error = 0.05, *P* < 0.001) and 0.25s.d. for the Full arm (standard error = 0.05, *P* < 0.001). Effects on food security were 0.20s.d. for the Capital arm (standard error = 0.05, *P* < 0.001), 0.19s.d. for the Psychosocial arm (standard error = 0.05, *P* <0.001), and 0.25s.d. for the Full arm (standard error = 0.05, *P* < 0.001).

**Table 2:** Intent-to-treat estimates for main standardized outcomes

		Capital (Full without Psychosocial)	Psychosocial (Full without Capital)	Full	N, d.f.	Full − Psychosocial (cash grant gross ME)	Full − Capital (Psychosocial component gross ME)	Capital −Psychosocial	Endpoint − midpoint for Capital	Endpoint − midpoint for Psychosocial	Endpoint − midpoint for Full
		Coefficient (standard error) (*P*)				Coefficient (standard error) (*P*)			Coefficient (standard error) (*P*)		
Daily consumption per adult equivalent	6m	0.14 (0.05) (0.003)	0.08 (0.05) (0.094)	0.22 (0.05) (0.000)	4,405 321	0.14 (0.05) (0.003)	0.09 (0.05) (0.069)	0.06 (0.04) (0.179)	−0.02 (0.05) (0.738)	0.10 (0.05) (0.058)	0.03 (0.05) (0.572)
	18m	0.12 (0.04) (0.008)	0.18 (0.05) (0.000)	0.25 (0.05) (0.000)	4,238 319	0.07 (0.05) (0.166)	0.13 (0.04) (0.003)	−0.06 (0.05) (0.200)			
Food security	6m	0.21 (0.05) (0.000)	0.08 (0.04) (0.058)	0.27 (0.04) (0.000)	4,476 321	0.18 (0.05) (0.000)	0.05 (0.05) (0.268)	0.13 (0.05) (0.013)	−0.01 (0.05) (0.863)	0.10 (0.06) (0.065)	−0.01 (0.05) (0.805)
	18m	0.20 (0.05) (0.000)	0.19 (0.05) (0.000)	0.25 (0.05) (0.000)	4,303 319	0.06 (0.05) (0.203)	0.05 (0.05) (0.285)	0.01 (0.05) (0.787)			
Household total revenue	6m	0.20 (0.05) (0.000)	0.15 (0.04) (0.001)	0.28 (0.05) (0.000)	4,476 321	0.13 (0.05) (0.010)	0.09 (0.05) (0.087)	0.04 (0.05) (0.345)	−0.02 (0.05) (0.729)	0.04 (0.05) (0.446)	0.02 (0.05) (0.650)
	18m	0.18 (0.05) (0.000)	0.19 (0.04) (0.000)	0.31 (0.05) (0.000)	4,303 319	0.11 (0.05) (0.018)	0.13 (0.05) (0.011)	-0.01 (0.04) (0.745)			
Beneficiary total revenue	6m	0.30 (0.05) (0.000)	0.21 (0.05) (0.000)	0.39 (0.06) (0.000)	4,476 321	0.18 (0.06) (0.003)	0.09 (0.06) (0.142)	0.09 (0.06) (0.118)	0.03 (0.05) (0.495)	0.03 (0.05) (0.543)	0.03 (0.06) (0.642)
	18m	0.34 (0.06) (0.000)	0.25 (0.06) (0.000)	0.42 (0.07) (0.000)	4,252 319	0.17 (0.07) (0.014)	0.08 (0.07) (0.265)	0.09 (0.06) (0.137)			
Mental health	6m	0.13 (0.04) (0.003)	0.10 (0.04) (0.016)	0.23 (0.04) (0.000)	4,476 321	0.13 (0.04) (0.002)	0.10 (0.04) (0.012)	0.02 (0.04) (0.560)	0.03 (0.05) (0.625)	0.11 (0.05) (0.022)	0.03 (0.05) (0.569)
	18m	0.15 (0.04) (0.000)	0.21 (0.04) (0.000)	0.26 (0.04) (0.000)	4,175 319	0.04 (0.04) (0.247)	0.10 (0.04) (0.007)	−0.06 (0.04) (0.149)			
Self efficacy	6m	0.12 (0.04) (0.005)	0.16 (0.04) (0.000)	0.29 (0.04) (0.000)	4,476 321	0.13 (0.04) (0.001)	0.17 (0.04) (0.000)	−0.04 (0.04) (0.310)	−0.02 (0.05) (0.743)	0.02 (0.05) (0.688)	−0.06 (0.05) (0.238)
	18m	0.10 (0.04) (0.019)	0.18 (0.04) (0.000)	0.23 (0.04) (0.000)	4,175 319	0.04 (0.04) (0.297)	0.12 (0.04) (0.004)	−0.08 (0.05) (0.089)			
Social cohesion and community closeness	6m	0.06 (0.05) (0.230)	0.13 (0.05) (0.012)	0.11 (0.04) (0.013)	4,476 321	−0.01 (0.05) (0.764)	0.06 (0.04) (0.215)	−0.07 (0.05) (0.175)	0.04 (0.06) (0.460)	0.08 (0.06) (0.200)	−0.02 (0.06) (0.789)
	18m	0.10 (0.05) (0.031)	0.20 (0.05) (0.000)	0.10 (0.05) (0.035)	4,160 319	−0.11 (0.05) (0.021)	−0.00 (0.04) (0.944)	−0.10 (0.05) (0.032)			
Control over earnings	6m	0.26 (0.04) (0.000)	0.21 (0.04) (0.000)	0.33 (0.04) (0.000)	4,476 321	0.12 (0.04) (0.004)	0.07 (0.04) (0.081)	0.05 (0.04) (0.231)	−0.01 (0.05) (0.768)	−0.05 (0.05) (0.312)	−0.07 (0.05) (0.112)
	18m	0.25 (0.05) (0.000)	0.16 (0.05) (0.000)	0.25 (0.04) (0.000)	4,252 319	0.09 (0.05) (0.045)	0.01 (0.04) (0.891)	0.08 (0.05) (0.075)			
Control over household resources	6m	0.03 (0.04) (0.496)	0.05 (0.04) (0.167)	0.06 (0.04) (0.142)	4,161 321	0.01 (0.04) (0.777)	0.04 (0.04) (0.386)	−0.03 (0.04) (0.522)	−0.04 (0.06) (0.493)	0.00 (0.06) (0.937)	−0.03 (0.06) (0.676)
	18m	−0.01 (0.05) (0.776)	0.06 (0.05) (0.234)	0.04 (0.05) (0.419)	4,055 319	−0.02 (0.04) (0.664)	0.05 (0.04) (0.218)	−0.07 (0.04) (0.110)			

Results presented are standardized OLS estimates that include controls for randomization strata and, where possible, baseline outcomes. We assign baseline strata means to households surveyed at the midpoint (median 6 months after the intervention) or endpoint (median 18 months after the intervention) but not at baseline, and we control for such missing values with an indicator. All outcomes in this table are standardized with respect to the control group. Extended Data Tables 1–8 show the impacts on outcomes in our pre-specified units and multiple hypothesis test corrections. Supplementary Tables 3, 4 present details on variable construction. Robust standard errors, clustered at the village level, and two-tailed *P*-values are shown in parentheses. ME denotes marginal effects.

The Capital and Full arms quickly achieved these levels of impacts, with no evidence of attenuation between the midpoint (a median of 6 months after the intervention) and endpoint (Capital: difference of −0.02s.d., standard error = 0.05, *P* = 0.738 for consumption and −0.01s.d., standard error = 0.05, *P* = 0.863 for food security; Full: difference of 0.03s.d., standard error = 0.05, *P* = 0.572 for consumption and −0.01s.d., standard error = 0.05, *P* = 0.805 for food security). By contrast, the Psychosocial arm had smaller short-term impacts on consumption and food security, but the effects doubled between the two time points (difference of 0.1s.d., standard error = 0.05, *P* = 0.058 for consumption; 0.1s.d., standard error = 0.06, *P* = 0.065 for food security), catching up with the Capital arm at endpoint (difference between Capital and Psychosocial: −0.06s.d., standard error = 0.05, *P* = 0.200 for consumption; 0.01s.d., standard error = 0.05, *P* = 0.787 for food security).

The three arms also increased total household and beneficiary revenues both at the midpoint and the endpoint, consistent with the effects on consumption (standardized effects in Fig. 1, Table 2; impacts in our pre-specified units in Extended Data Tables 2, 3). At the endpoint, revenues for the beneficiary increased by 0.34s.d. in the Capital arm (standard error = 0.06, *P* < 0.001), 0.25s.d. in the Psychosocial arm (standard error = 0.06, *P* < 0.001) and 0.42s.d. in the Full arm (standard error = 0.07, *P* < 0.001), and household income increased by 0.18s.d. in the Capital arm (standard error = 0.05, *P* < 0.001), 0.19s.d. in the Psychosocial arm (standard error = 0.04, *P* < 0.001) and 0.31s.d. in the Full arm (standard error = 0.05, *P* < 0.001).

These effects were driven largely by increases in off-farm business revenues and activities in all three arms (Extended Data Tables 2–4, Supplementary Tables 6, 9a, 9b, 11). Yearly household business revenues at the endpoint increased in the Capital arm (US$318.30, standard error = US$90.4, *P* < 0.001), Psychosocial arm (US$333.50, standard error= US$88, *P* <0.001) and Full arm (US$540.50, standard error = US$96.3, *P* <0.001). Following the intervention, households owned more off-farm businesses, often engaging in commerce or processing agricultural products, and beneficiaries allocated more labour to these businesses.

Increases in livestock and agricultural revenues also contributed to the overall impact on revenues, although less so than off-farm businesses, and with notable differences between treatment arms and over time (Extended Data Tables 2–5, Supplementary Tables 7–9b). Household livestock revenues increased at the endpoint, especially in the Capital arm (US$70.4, standard error = US$17.6, *P* < 0.001) and Full arm (US$72.6, standard error = US$18.2, *P* < 0.001), with marginally larger effects relative to the Psychosocial arm (differences of US$35.1, standard error = US$19.6, *P* = 0.074 and US$33, standard error = US$19.4, *P* = 0.09, respectively). The Capital and Full arms increased livestock asset value and labour allocated to livestock at both follow-ups, whereas the Psychosocial arm induced relatively less investment in livestock. By contrast, household harvest revenues at endpoint increased in the Psychosocial arm (US$91.1, standard error = US$23, *P* < 0.001) and Full arm (US$80, standard error = US$21.6, *P* < 0.001), with smaller effects in the Capital arm (differences of US$59.53, standard error = US$24.5, *P* = 0.016 and US$48.45, standard error = US$22.3, *P* = 0.03, respectively). This set of results suggests that the cash grants were partly used to accumulate livestock, whereas the psychosocial components contributed to the increase in agricultural revenues.

The cash grant and psychosocial components also had different effects on beneficiary or household revenues (Extended Data Tables 2, 3). By comparing the Full arm to the Psychosocial arm, we find that the cash grant contributed to increases in business revenues at the endpoint for the beneficiary (US$112.4, standard error = US$50.4, *P* = 0.026) and their household (US$207, standard error = US$97.8, *P* = 0.035). This suggests that the woman beneficiary used at least part of the grant to grow her own business. By contrast, comparing the Full arm to the Capital arm shows that the psychosocial components mostly induced an increase in household revenues at the endpoint (US$290.1, standard error = US$112.7, *P* = 0.011), stemming from higher household business revenues (US$222.2, standard error = US$99.5, *P* = 0.026) and harvest values (US$48.5, standard error = US$22.3, *P* = 0.030). This suggests that the psychosocial components had indirect effects on other household members, whereas the cash grant increased the individual beneficiary’s own earnings more directly. However, these alternative pathways should be interpreted with caution; the cash grant and psychosocial components generated a mix of changes in income-generating activities, and there were only rare instances of differences in impacts between the Psychosocial and Capital arms.

Finally, all three arms led to increases in participation in savings groups (Capital: 0.31, standard error = 0.03, *P* < 0.001; Psychosocial: 0.27, standard error = 0.03, *P* < 0.001; Full: 0.33, standard error = 0.03, *P* < 0.001) and amounts saved in these groups at the endpoint (Capital: US$15.7, standard error = US$3, *P* < 0.001; Psychosocial: US$11.6, standard error = US$2.8, *P* < 0.001; Full: US$20.1, standard error = US$3.53, *P* < 0.001) (Supplementary Table 10a). Both the Psychosocial and Full arms also showed sustained increases in a household asset index (Capital: 0.04s.d., standard error = 0.06, *P* = 0.478; Psychosocial: 0.13s.d., standard error = 0.06, *P* = 0.020; Full: 0.15s.d., standard error = 0.06, *P* = 0.007) (Supplementary Table 10a).

Village-level randomization implies that the estimated treatment effects for impacts on eligible households were not biased by spillovers, as long as the treatment did not generate cross-village spillovers. However, the programme could have generated indirect effects on non-eligible households within treatment villages. We cannot test for this directly, given the absence of data on non-eligible households in treatment and control villages. However, we were able to test for specific intermediary outcomes that were potential mediators of spillovers to non-participants. Supplementary Table 12 shows no evidence of adverse or advantageous indirect effects on land market or community tension, but points to some increase in labour usage and transfers. We found little evidence of changes in food prices (Supplementary Table 13).

## Psychosocial well-being and women's empowerment

There were also widespread improvements across dimensions of psychological and social well-being for all arms. Women’s psychological well-being—including mental health, self-efficacy, and future expectations—improved at both time points in all arms (Fig. 1, Table 2, Extended Data Table 6, Supplementary Tables 14–16). For instance, positive effects on mental health at the endpoint—including life satisfaction, inner peace, and depression—ranged from 0.15s.d. in the Capital arm (standard error = 0.04, *P* < 0.001) to 0.21s.d. in the Psychosocial arm (standard error = 0.04, *P* < 0.001) and 0.26s.d. in the Full arm (standard error = 0.04, *P* < 0.001) (Supplementary Table 14).

There were, however, notable differences in temporal trends in psychological well-being across the Capital and Psychosocial arms (Extended Data Table 6). At the midpoint, the effects of the Capital and Psychosocial arms tend to be lower than those of the Full arm across the measures of psychological well-being—for instance on mental health (Capital arm: difference of −0.1s.d., standard error = 0.04, *P* = 0.012; Psychosocial arm: difference of −0.13s.d., standard error = 0.04, *P* = 0.002). Yet at the endpoint there was no evidence of a difference between the Psychosocial arm and the Full arm (difference of 0.04s.d., standard error = 0.04, *P* = 0.247 for mental health; 0.04s.d., standard error = 0.04, *P* = 0.297 for self-efficacy; 0.05s.d., standard error = 0.04, *P* = 0.222 for future expectations). In the case of mental health, this was driven in part by a doubling of impacts between the midpoint and the endpoint in the Psychosocial arm (difference of 0.11s.d., standard error = 0.05, *P* = 0.022). Further, endpoint effects on self-efficacy were marginally larger in the Psychosocial than the Capital arm (difference of 0.08s.d., standard error = 0.05, *P* = 0.089).

The arms positively, although differentially, affected two dimensions of women’s empowerment—namely social well-being and social capital (Extended Data Table 7), and individual control over earnings and household decision making (Extended Data Table 8). The Psychosocial and Full arms, which included the psychosocial components, exhibited substantial impacts on the social dimension. The Capital arm showed strong impacts on control over earnings, although we find no evidence of effects on household decision-making.

All arms had significant positive effects on women’s social well-being and social capital among their community, including increased financial support, social support, social standing and collective action (Extended Data Table 7, Supplementary Tables 17–22). For instance, effects on social support at the endpoint ranged from 0.13s.d. in the Capital arm (standard error = 0.04, *P* = 0.004) to 0.18s.d. in both the Psychosocial (standard error = 0.05, *P* < 0.001) and Full arms (standard error = 0.04, *P* < 0.001). In addition to instrumental support, all arms increased normative support for women’s economic engagement at the midpoint (Capital: 0.15s.d., standard error = 0.04, *P* = 0.001; Psychosocial: 0.19s.d., standard error = 0.04, *P* < 0.001; Full: 0.19s.d., standard error = 0.04, *P* <0.001); at the endpoint, significant impacts were observed in the Psychosocial and Full arms in which the community sensitization targeted social norms directly (Capital: 0.08s.d., standard error = 0.05, *P* = 0.109; Psychosocial: 0.11s.d., standard error = 0.05, *P* = 0.014; Full: 0.17s.d., standard error = 0.05, *P* = 0.001).

Similarly, whereas the Psychosocial and Full arms increased social cohesion and community closeness at the endpoint (Psychosocial: 0.20s.d., standard error = 0.05, *P* < 0.001; Full: 0.10s.d., standard error = 0.05, *P* = 0.035), the effect of the Psychosocial arm was twice as large as that of the Full and Capital arms (Full: difference of 0.11s.d., standard error = 0.05, *P* = 0.021; Capital: 0.10s.d., standard error = 0.05, *P* = 0.032) (Table 2, Extended Data Table 7). Supplementary Table 21 reveals that the positive impact of the Psychosocial arm was associated with reduced reports of personal enemies and an increased interest in caring for the village, the latter of which aligns with the life skills trainings centred on community leadership. Moreover, this arm may have avoided an observed marginal negative effect of the cash grants on indicators of relationship strain, including enemyship and differentiation from one’s community. Despite these relative differences across arms, we found no evidence of a change in community tensions in any arm (Supplementary Table 21).

In addition to community relationships, we also examined women’s intrahousehold relationships (Extended Data Table 8, Supplementary Tables 23, 24). We observed no evidence of effects in any of the arms at the endpoint on an overall intrahousehold index combining both partner-level and household-level dynamics (Capital: 0.02s.d., standard error = 0.04, *P* = 0.634; Psychosocial: 0.04s.d., standard error = 0.04, *P* = 0.323; Full: −0.01s.d., standard error = 0.04, *P* = 0.787). However, the Psychosocial arm improved the sub-index of partner dynamics (Psychosocial: 0.12s.d., standard error = 0.04, *P* = 0.007; Capital: 0.01s.d., standard error = 0.05, *P* = 0.752; Full: 0.02s.d., standard error = 0.04, *P* = 0.714), driven by increases in women’s perceived closeness with their partner and comfort in disagreeing with them (Supplementary Table 23). This improved relationship quality may be related to the positive effects on other household members’ revenues seen in the Psychosocial arm. Of note, across all time points and arms, there was no evidence of an increase in the perceived prevalence of domestic violence in the community; instead, we observed a reduction at the endpoint in the Psychosocial and Full arms (Capital: 0.02s.d., standard error = 0.04, *P* = 0.593; Psychosocial: −0.08s.d., standard error = 0.04, *P* = 0.064; Full: −0.11s.d., standard error = 0.04, *P* = 0.008) (Extended Data Table 8, Supplementary Table 24).

The second dimension of women’s empowerment that we assessed was individual control over earnings and household decision-making (Table 2, Extended Data Table 8, Supplementary Tables 25, 26). All arms had positive and sustained effects on the index of women’s control over their own earnings and productive activities at the endpoint (Capital: 0.25s.d., standard error = 0.05, *P* < 0.001; Psychosocial: 0.16s.d., standard error = 0.05, *P* < 0.001; Full: 0.25s.d., standard error = 0.04, *P* < 0.001). The effect of the Capital arm and Full arm on this index was marginally larger than the Psychosocial arm (differences of 0.08s.d., standard error = 0.05, *P* = 0.075 and 0.09s.d., standard error = 0.05, *P* = 0.045, respectively), driven in part by increases in the probability of owning livestock and control over livestock revenues (Supplementary Table 25). These patterns are consistent with observed increases in women’s share of total household revenue (Capital: 0.06, standard error = 0.02, *P* < 0.001; Psychosocial: 0.03, standard error = 0.01, *P* = 0.035; Full: 0.06, standard error = 0.01, *P* < 0.001), with effects being marginally larger in the Capital than the Psychosocial arm (difference: 0.03, standard error = 0.02, *P* = 0.068) (Extended Data Table 8).

However, there is no evidence that these increases in women’s absolute and proportional revenues translated into broader increases in their decision-making power over household resources in any arm at the endpoint (Capital: −0.01s.d., standard error = 0.05, *P* = 0.776; Psychosocial: 0.06s.d., standard error = 0.05, *P* = 0.234; Full: 0.04s.d., standard error = 0.05, *P* = 0.419) (Table 2, Extended Data Table 8, Supplementary Table 26). Since women were contributing well below 50% of total household revenues (around 27% in the control group at the endpoint), a small increase in this share (by 3–6 percentage points) may have been insufficient to affect overall bargaining power (Extended Data Table 8).

In sum, we find that the Psychosocial and Capital arms both increased women’s psychosocial well-being and empowerment, but in distinct ways. Compared with the Psychosocial arm, women in the Capital arm experienced increased autonomy, including greater control over their own earnings and productive activities, and increased relative share of household revenues. By comparison, in the Psychosocial arm, women strengthened social relationships with their community and their partner, built social capital and experienced increases in revenues primarily through other household members’ activities. Although we were unable to determine directionality of effects among these outcomes, in the Psychosocial arm it is noteworthy that mental health, social cohesion, partner dynamics and household economic outcomes all tend to improve over time.

## Cost-effectiveness of treatment arms

The costs of these interventions were low: US$263 per beneficiary for the Psychosocial arm, US$482 for the Capital arm and US$584 for the Full arm (Extended Data Table 9). The psychosocial interventions were cheaper (US$102, panel 1, measures 2 and 4) than the cash grant (US$321, panel 1, measure 6).

For our primary analysis on cost-effectiveness, we use effects on consumption to estimate benefits. Any programme that posits impacts on multiple outcomes has an empirical and philosophical challenge in determining the optimal outcome. Using consumption has four primary advantages: it is a manifestation of both current and projected economic well-being; it is typically more precisely measured than income or asset values; it encapsulates indirectly the benefits of other outcomes (for example, one may prioritize income generation, but only because it allows individuals to consume more); and finally, it is a common outcome across other interventions (for example, cash transfers), thus expanding the comparability of studies for policy purposes. We also recognize that a potentially more holistic primary outcome could be life satisfaction, and discuss this below.

The comparison of programme costs with estimated effects on consumption shows that the treatment arms were cost-effective under most assumptions. Extended Data Table 9 presents the benefit–cost ratios and internal rates of return (IRRs). The results reveal a particularly high cost-effectiveness of the treatment arms with psychosocial components. We cannot reject equality of the benefit–cost ratios between the Psychosocial and Full arms (difference of 1.709 − 1.275 = 0.434, standard error = 0.43, *P* = 0.32), but the Capital arm has a lower benefit–cost ratio than both the Full arm (difference of 0.796 − 1.275= −0.479, standard error = 0.22, *P* = 0.03) and the Psychosocial arm (difference of 0.796 − 1.709 = −0.913, standard error = 0.42, *P* = 0.03). Note that these ratios do not take into account effects on assets.

**Fig. 2:**
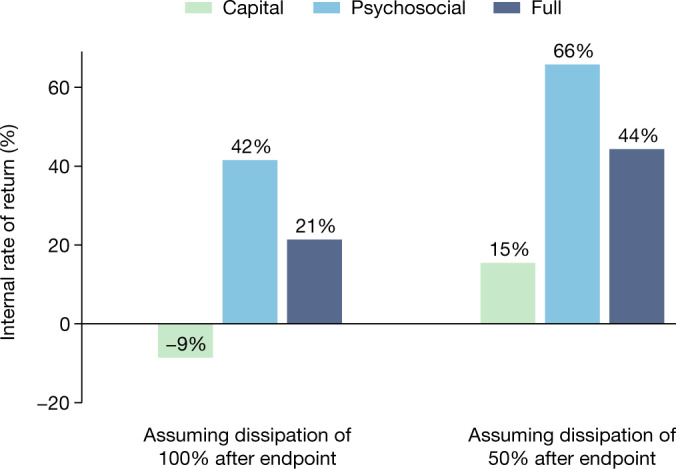
Internal rates of return. Internal rates of return are calculated using the annual cost and benefit data shown in Extended Data Table 9.

Remarkably, the IRRs are 42% for the Psychosocial arm and 21% for the Full arm, based on consumption effects observed by the endpoint, without assuming any further effects (Fig. 2). Assuming a dissipation of impacts of 50% per year after the endpoint, the IRRs are 66% for the Psychosocial arm and 44% for the Full arm, and the Capital arm also reaches a positive IRR (15%). Assuming sustained impacts gives IRRs of 95%, 73% and 48%, respectively.

Separately from the cost–benefit analysis based on consumption, we used impacts on life satisfaction to benchmark cost-effectiveness related to psychosocial well-being (Supplementary Table 28). The cost per 0.1s.d. increase in life satisfaction is estimated at US$181 for the Psychosocial arm, US$246 for the Full arm and US$451 for the Capital arm—a ranking consistent with the results obtained on household consumption. Note, however, that improvements in life satisfaction should not be considered as additive to consumptions gains (life satisfaction may or may not increase because of increased consumption, or vice versa).

## Discussion

Our findings show that three modalities of a multi-faceted intervention induced widespread improvements in consumption, revenues and psychosocial well-being. Of note, the arm primarily addressing capital constraints produced both economic and psychosocial impacts, and the arm primarily addressing psychosocial constraints produced both psychosocial and economic impacts.

We observed differences in pathways across treatments and over time. The Full arm produced the largest effects on consumption and revenues at the midpoint, with sustained effects at the endpoint. In contrast to recent evidence on cash grants^45–47^, we found no evidence of dissipation of the effect of the Capital arm treatment over time. Across various economic and psychosocial outcomes, we found that the impacts of the Psychosocial arm increased over time, in line with the literature on social psychological interventions^16,48^. All arms increased business revenues. In addition, the Full and Capital arms had relatively larger effects on livestock than the Psychosocial arm, whereas the Full and Psychosocial arms had larger effects on agricultural revenues than the Capital arm. Finally, the paths towards women’s empowerment also differed, with the Capital arm increasing control over earnings and activities, whereas the Psychosocial arm strengthened relationships and expanded sources of instrumental and normative social support.

Our experimental design has two limitations. First, it does not allow a simple measure of complementarities between the Psychosocial and the Capital interventions, because the ‘core’ components were included in both arms. It was not possible to include a fourth treatment arm that included only the core components. Second, we measured impacts for eligible households inclusive of potential within-village spillovers, but cannot directly isolate these within-village spillovers^49^. Other studies of multifaceted programmes have not found evidence of strong spillovers^6^, and we found thin evidence of impacts on mediators of spillovers. Lastly, we find little evidence of impact heterogeneity (see, for example, Supplementary Tables 29, 30), but plan to further study this in the future once able to combine data from similar experiments in three other Sahel countries.

This study has direct policy implications. The multi-faceted interventions were delivered through a government-led national cash transfer programme. Sustained effects were obtained at low cost, leading to considerably higher benefit-cost ratios than graduation programmes implemented by NGOs elsewhere^6,25^. Both the Psychosocial and Full arms had rates of returns that were higher than those of the Capital arm and were cost-effective 18 months after the intervention, highlighting the value of addressing psychosocial constraints—not just primarily capital constraints—to open pathways out of extreme poverty.

## Methods

The research protocol was approved by Innovations for Poverty Action Institutional Review Board and preregistered in the AEA RCT Registry (study 0002544). The pre-analysis plan is registered at https://www.socialscienceregistry.org/versions/52534/docs/version/document. All survey participants completed informed consent. They were not compensated for their time as they were all part of the national cash transfer programme.

### Interventions

#### Niger context and cash transfer programme

Niger, one of the poorest countries in the world, has a rural poverty rate^50^ of 51.4% and ranks last in human development indicators^51^. Landlocked in the Sahel, its population is highly exposed to climatic shocks and food insecurity. More than 90% of Nigerien households have a member engaged in agriculture, but agricultural production is dominated by low-productivity subsistence farming with little market access. Only about 25% of farmers commercialize any crop and only 10% of villages have a permanent market. Non-agricultural activities are scarce as a primary occupation in rural areas (less than 10%) but are a secondary occupation for about a third. They mostly consist of agricultural transformation and trading. The wage sector only employs 4% of the workforce, mostly in public sector jobs concentrated in the capital. More than a third of Nigerien women do not participate in the labour force, overwhelmingly because of the burden of housework^52^.

After repeated humanitarian interventions in response to shocks and food insecurity, the Government of Niger set up a social protection system. Its cornerstone was a national cash transfer programme that provided monthly payments of 10,000 XOF for two years (US$15.95, US$38.95 purchasing power parity-adjusted (PPP)), which represented approximately 11% of yearly household consumption for targeted poor rural households. The programme was rolled out in three main phases and reached 100,000 beneficiary households between 2012 and 2019. We studied the 3rd phase of the programme, implemented from 2016 to 2019, which reached approximately 22,500 households. The cash transfers were unconditional but were delivered with child development promotion activities for all households.

The national cash transfer programme applied geographical targeting before using household-level poverty targeting. The programme selected the communes with highest poverty rates in all eight regions in the country. In practice, most selected communes were rural. Within communes, all villages were eligible and public lotteries were organized to select beneficiary villages. Poverty-targeting methods were applied to determine the beneficiary households. Within selected households, a woman over 20 was the recipient of the cash transfers.

#### Multi-faceted interventions

To address constraints to participation in income-generating activities and economic diversification, the multi-faceted programme combined three main sets of interventions and was delivered on top of the regular cash transfer programme^53^. The core components promoted financial inclusion, basic micro-entrepreneurship skills and market access. A second component addressed capital constraints by providing a lump-sum cash grant intended for productive purposes. A third component provided psychosocial interventions that aimed to strengthen aspirations and interpersonal and intrapersonal skills, as well as to address gender and social norms. Supplementary Appendix 1 describes how the intervention was delivered through the government-led national cash transfer programme.

##### Core components included in all three treatment variants

1. Coaching. The coaching component facilitated the delivery and coordination of the various interventions. Beneficiaries formed groups of 15 to 25 members and selected a coach to mentor them throughout the programme. Coaches were men or women from the village, generally selected for their capacity to advise on income-generating activities and to represent the group for service providers and market agents. Coaches facilitated the implementation of group-based programme activities, including promoting the attendance of beneficiaries at meetings and coordinating with service providers. They led group-level coaching sessions, during which challenges and opportunities for income-generating activities were discussed. The group-level coaching sessions occurred during weekly savings group meetings, as described below. Coaches also provided some individualized follow-up to beneficiaries.

2. Saving groups. The groups of beneficiaries formed a village savings and loans association (VSLA), with initial training from the coach. The group received a VSLA kit, elected members to leadership positions and determined the rules governing the association. Key decisions included the cost of a saving ‘share’, maximum loan size, interest rate and duration of a savings cycle. Group members also defined other parameters, such as a mandatory contribution to an emergency fund and penalties. At weekly meetings, members purchased between one and five shares in the savings fund, contributed a fixed amount to the emergency fund, and could take out a short-term loan from the savings fund. A full savings cycle lasted between 9 and 12 months, at which point the accumulated savings, interest, and penalty fees were shared among members in proportion to the number of savings shares owned by each member.

3. Micro-entrepreneurship training. A week-long micro-entrepreneurship training was delivered to the groups. The curriculum was adapted from the International Labour Organization’s Start and Improve Your Business (SIYB) level 1 training, which is tailored to non-literate participants. The curriculum covered fundamental micro-entrepreneurship skills, including basic accounting and management principles, market research, planning and scheduling, saving, and investing. In addition, the training focused on the choice of livelihood activities and the preparation of a basic business plan.

4. Access to markets. Coaches were trained to deliver information sessions on market access. Depending on production cycle timing, they held group sessions to discuss where to buy inputs for agricultural activities, how to choose suppliers, or where to sell products.

##### The capital component

A lump-sum cash grant of 80,000 XOF (US$127 (US$311 in 2016 PPP)) was provided to promote investments in income generating activities. Payments were not conditional on participation in other programme activities.

##### The psychosocial components

The psychosocial components included community-level programming, which consisted of community sensitization on social norms and aspirations, and individual-level programming, which consisted of life skills training for the beneficiaries. While they were relatively light, they aimed to trigger three main mechanisms: (1) to build personal psychological assets, including self-efficacy, self-worth, aspirations, and optimism about the future, while developing behavioural skills related to interpersonal communication, problem-solving, leadership, and goal setting; (2) to promote social empowerment, including social standing in the community, community support and solidarity, and supportive social norms around women’s income generating activities; and (3) to foster positive intra-household dynamics, including interpersonal trust, closeness, and conflict resolution, as well as women’s decision-making power and control over resources. We also expected several of these mechanisms to improve mental health. Supplementary Appendix 2 provides a detailed description of the psychosocial components.

1. Community sensitization on aspirations and social norms. The full community, including elders, economic and traditional leaders, and programme beneficiaries and their husbands (or other family members), were invited to attend a video screening and community discussion. Programme staff projected a short video in local languages that depicted the story of a couple that overcomes household and personal constraints and develops economic activities, with support from family and their community. As a result, they become more economically resilient. After the screening, trained facilitators guided a public discussion on social norms, aspirations, and community values. The sensitization integrated multiple approaches to social and behaviour change. These include role models in the video, peer effects in the audience construction, goal setting and social consensus techniques in the discussion, and values alignment in both the video and discussion.

2. Life skills training. A week-long life-skills training was organized for groups of beneficiaries. Grounded in participatory, problem-centred learning, the training included role plays, games, and case studies. The nine modules of the curriculum focused on building skills for effective decision-making, problem-solving, goal setting, interpersonal communication, and women’s leadership, while simultaneously building self-worth, self-efficacy, and aspirations. In addition, discussions prompted participants to relate their economic goals to broader values and to spousal, gender, and generational roles. The training was delivered by private trainers contracted by the government through small firms.

### Randomized controlled trial design and data

#### Experimental design

In total, approximately 100,000 households have participated in the Niger cash transfer programme since 2012. This study focused on the 3rd wave of the programme, which reached 22,507 beneficiary households in 329 villages in 17 communes of the 5 most populous of Niger’s eight regions (Dosso, Maradi, Tahoua, Tillaberi and Zinder; see Supplementary Fig. 1 for a map of study communes). All of the villages that received cash transfers in the 17 communes were included in our sample. After grouping small neighbouring villages that have less than 8 beneficiaries for ease of programme operations, 322 villages entered the randomization.

The study is a cluster-randomized controlled trial in which villages with existing cash transfer beneficiaries were randomly allocated to one of the four arms (Table 1): one control group (81 villages), and three treatment arms with variants of the intervention components (80 villages in Capital, 78 villages in Psychosocial and 83 villages in Full). Within each village there was no additional randomization across households, and thus all eligible households within each village received the same treatment.

Randomization of the villages was stratified by the 17 communes and the targeting method used to select cash transfer beneficiaries in each village (which is part of a complementary study^54^) and took place in public lotteries. To promote the transparency gained from public lotteries while maintaining balance across targeting methods, we proceeded in two stages. First, for each commune we randomly assigned villages into four lists stratified by targeting method. The strata were based on a categorical variable with four values, one for each of three randomized targeting methods and a fourth for not being part of the targeting study. This stage did not assign the experimental arm label to each list. Second, we organized a public lottery in each of the 17 communes to randomly assign each list to one of the four experimental arms. The lottery was organized by the cash transfer programme government team and held in the capital of the commune in the presence of village chiefs or elders.

One limitation of this design is that we could not include a fourth treatment arm with core components only. While we can therefore test the importance of including capital on top of the core and psychosocial components (by comparing the Full arm to the Psychosocial arm), if the psychosocial components change the marginal value of the capital, then we would not estimate the effect of providing capital as part of a programme without those psychosocial components. Likewise, we test the importance of including psychosocial components on top of a design that includes the lump-sum capital transfer (by comparing the Full arm to the Capital arm). Note that earlier work on the Niger national cash transfer programme has shown that cash transfers (either alone or combined with group savings facilitation as in the core component) increased savings and livestock accumulation, but had little average effects on earnings from income-generating activities or economic diversification^55,56^.

#### Sampling, timeline and data

Out of the 22,507 cash transfer beneficiaries that were assigned to the 4 treatment variants, 4,712 households were drawn into a sample for data collection (1,206 households in control, 1,191 households in capital, 1,112 households in psychosocial and 1,203 households in full). Before the study, we conducted power calculations assuming an intracluster correlation of 0.10 (based on data from Ghana^6^ and a Niger national household survey) and equal sized arms. To maximize power, we sampled all villages in this phase. Sampling 15 households per village allowed for minimum detectable sizes of 0.057s.d. between arms, before adjusting for baseline outcomes or strata.

**Extended Data Fig. 1:**
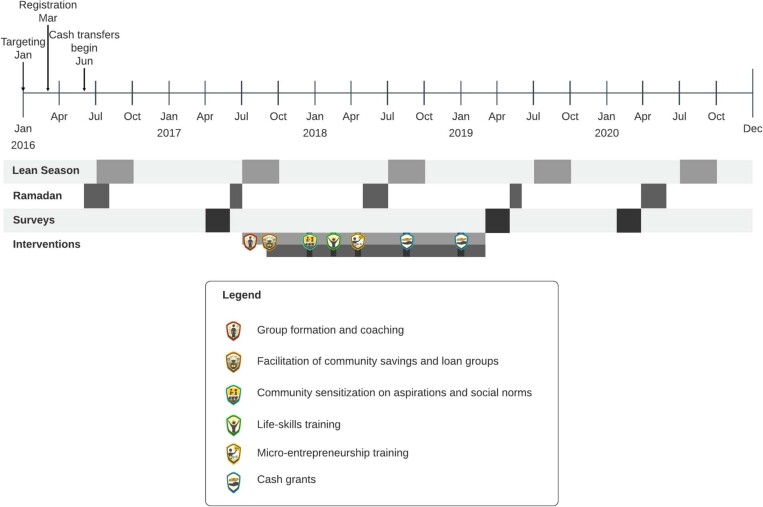
Study Timeline. Notes: Groups were formed around September 2017. Savings activities began shortly thereafter, and other components were then rolled out as per above throughout 2018 and early 2019. Cash grant disbursement was randomized in two phases: cash grants were disbursed in July-August 2018 for the early group, and in November-January 2019 for the late group. Surveys were concluded before Ramadan in 2017, 2019 and 2020, and were stopped in March 2020 due to the onset of the COVID-19 pandemic.

Extended Data Figure 1 summarizes the study timeline. Baseline data collection took place between April and June 2017. The public lotteries took place after data collection in June 2017. The intervention was delivered between September 2017 and January 2019. Two follow-up surveys were collected. The midpoint occurred in February and March 2019, a median of 6 months (3 to 9 months) post-intervention (that is, after the delivery of the lump-sum grant in treatment arms with the capital component). The endpoint survey occurred a year later in February and March 2020, a median of 18 months post-intervention (after the delivery of the cash grants in treatment arms with the capital component). Survey teams, blind to treatment status, were assigned to villages; but the participant could reveal treatment status in the last module of the midpoint survey. During the fieldwork, a remote team checked and updated the field plan for treatment balance across teams and survey weeks.

Supplementary Table 1 reports descriptive baseline statistics and balance tests across the experimental arms for a set of pre-specified variables. The sample was extremely poor. Fewer than 8% of beneficiaries were literate and they had, on average, less than 0.5 year of schooling. Beneficiaries were 38 years old on average, and 99% were female. They took about 70 min to get to the nearest market. On the whole, the random assignment created well-balanced experimental arms.

At the midpoint and endpoint, 95.0% and 91.3% of baseline households were successfully interviewed, respectively. Attrition was balanced across the treatment arms (Supplementary Table 1, bottom panel).

Supplementary Table 2 documents compliance with treatment assignment based on administrative data. Across all treatment arms, the participation rate in VSLA meetings was 92%, and the attendance rate in the micro-entrepreneurship training was 95%. By design, there was more variation in the delivery of individual coaching visits, with on average 52% of beneficiaries receiving coaching visits each month. Across the Psychosocial and Full treatment groups, 94% of beneficiaries attended life skills training and 89% attended the community sensitizations. Across the Capital and Full treatment groups, 99.9% of beneficiaries received the cash grants.

#### Estimation strategy

We estimate separate intent-to-treat treatment effects for each (treatment) arm for pre-specified outcomes based on the following specification:

1 $${Y}_{i,t}={\beta }_{p,t}{T}_{{\rm{P}}{\rm{s}}{\rm{y}}{\rm{c}}{\rm{h}}{\rm{o}}{\rm{s}}{\rm{o}}{\rm{c}}{\rm{i}}{\rm{a}}{\rm{l}}}+{\beta }_{c,t}{T}_{{\rm{C}}{\rm{a}}{\rm{p}}{\rm{i}}{\rm{t}}{\rm{a}}{\rm{l}}}+{\beta }_{f,t}{T}_{{\rm{F}}{\rm{u}}{\rm{l}}{\rm{l}}}+\delta {Y}_{i,0}+{\boldsymbol{\gamma }}\,+{{\rm{\varepsilon }}}_{i,t}$$

where *Y*_*i*,*t*_ is the outcome of interest for household or individual *i* at midpoint or endpoint (*t* = 1 or *t* =2); *T*_Psychosocial_, *T*_Capital_ and *T*_Full_ are indicators for village assignment to the Psychosocial, Capital, or Full treatment arm; **γ** is a vector of randomization strata fixed effects. We estimate this specification separately for each follow-up. Standard errors are clustered at the village level, the unit of randomization. To increase precision, we include a control for the outcome at baseline (*Y*_i,0_) when available. When not available for a subset of households, we set the baseline control to the mean outcome in the randomization strata and include a dummy for a missing measurement at baseline. *β*_*p*,*t*_, *β*_*c*,*t*_ and *β*_*f*,*t*_ are the main parameters of interest. They capture the impact of each treatment arm for regular cash transfer beneficiary households.

To estimate the added value of the cash grant and psychosocial components (or gross marginal effects), we report three additional tests for each data collection round:

First (H_1_), we test the added value (or gross marginal effect) of the cash grant with H_0_: *β*_*f*_ − *β*_*p*_ = 0.

Second (H_2_), we test the added value (or gross marginal effect) of the psychosocial interventions (the community sensitization intervention and life skills training) with H_0_: *β*_*f*_ − *β*_*c*_ = 0.

Third (H_3_), we test for equality of treatment effects between the Capital and Psychosocial arms, which is the same as testing equality of gross marginal effects of the cash grants and psychosocial interventions, with: H_0_: *β*_*c*_ − *β*_*p*_ = 0.

Note that gross marginal effects are inclusive of complementarities with the core components.

Finally, we test for equality of treatment effects between data collection rounds to uncover any temporal effects (for each treatment arm separately).

We conduct our analysis in accordance with a pre-analysis plan. We pre-specified in our pre-analysis plan two primary economic outcomes: consumption per adult equivalent and the (reverse of) FAO’s Food Insecurity Experience Scale (FIES^57,58^). Although it was pre-specified as a secondary outcome, we also report the Food Consumption Score (FCS^59^ in the main outcome Extended Data Table 1, since it provides another measure of food security that captures the beneficiary women’s dietary diversity. Other notable deviations include slight changes of the grouping of outcome variables for expository clarity, and the presentation of standardized effect sizes for key outcomes. Supplementary Appendix 4 summarizes deviations from the pre-analysis plan.

We pre-specified a range of intermediary outcomes to capture the pathways through which the interventions were expected to affect the primary economic outcomes, as well as a range of psychosocial well-being measures (see Supplementary Appendix 3 for more information on psychosocial outcomes). We discuss key intermediary outcomes in the results section, with additional results in the annex. Supplementary Tables 3, 4 provide more details on variable construction.

To account for multiple hypotheses, we calculate *P*-values adjusted within each treatment arm within predetermined families of variables, and report corrections in Supplementary Table 5. Following our pre-analysis plan, we also calculate *P*-values controlling for both the false discovery rate (FDR) and the family-wise error rate (FWER). The FWER is our preferred correction and is displayed in the extended data tables.

#### Cost–benefit calculations

The intervention was designed as low-cost to ensure it could be scaled-up through government systems. Extended Data Table 9 details programme costs obtained from administrative data, per beneficiary of each intervention arm. In 2016 PPP US$, total costs were US$263 for the Psychosocial arm, US$482 for the Capital arm and US$584 for the Full arm. We do not account for cash transfer programme costs (including targeting or payment) since these were incurred for the control group as well. The programme costs were substantially lower than similar graduation programmes implemented in other contexts: US$1,475 PPP in India, US$4,215 PPP in Ethiopia, US$5,483 PPP in Ghana, US$6,044 PPP in Pakistan^6^ and US$6,183 PPP in Afghanistan^25^.

We perform a conservative calculation of estimated benefits that only considers impacts on consumption (obtained from the specification in equation (1)), without accounting for impacts on assets or psychosocial well-being. Cumulated consumption impacts are calculated as half the impacts on yearly consumption at midpoint plus impacts on yearly consumption at endpoint. We consider various scenarios regarding the sustainability of impacts after endpoint. First, we consider zero impacts after endpoint (scenario A). We then consider various yearly rates of dissipation of impacts, including 75% (scenario B1), 50% (scenario B2) and 25% (scenario B3). Lastly, we assume impacts are sustained in perpetuity (scenario C), as in the benchmark case used by some other studies^6^. We use a 5% discount rate when calculating benefit-cost ratios.

We also perform cost-effectiveness calculations of benefits to psychological well-being. For each treatment arm, we compute the cost per 0.1s.d. increase in life satisfaction, as assessed by the Cantril ladder at endpoint. We choose a benchmark of 0.1s.d. given it is approximately the meta-analytic effect of economic interventions on psychological well-being^60^. We additionally compute the cost per case of depression averted within each arm, using the CESD-10 self-report measure of depression at both follow-ups.

### Reporting summary

Further information on research design is available in the Nature Research Reporting Summary linked to this paper.

## Online content

Any methods, additional references, Nature Research reporting summaries, source data, extended data, supplementary information, acknowledgements, peer review information; details of author contributions and competing interests; and statements of data and code availability are available at 10.1038/s41586-022-04647-8.

### Supplementary information

Supplementary Information This file contains Supplementary Notes, Supplementary Fig. 1, and Supplementary Tables 1–30. Supplementary Notes: Part 1 details the implementation of the government-led national cash transfer programme. Parts 2, 3 and 4 provide additional information on the psychosocial components and outcomes and describes deviations from the pre-analysis plan. Supplementary Figures: Shows a map of communes in the study sample. Supplementary Tables: Details: (1) balance and attrition; (2) compliance; (3) variable definitions and construction notes; (4) multiple hypothesis test corrections, (5) extended results (economic outcomes, spillover mediators, and index subcomponents), (6) cost-benefit calculations, and (7) heterogeneity results.

Reporting Summary

## Figures and Tables

**Extended Data Table 1:**
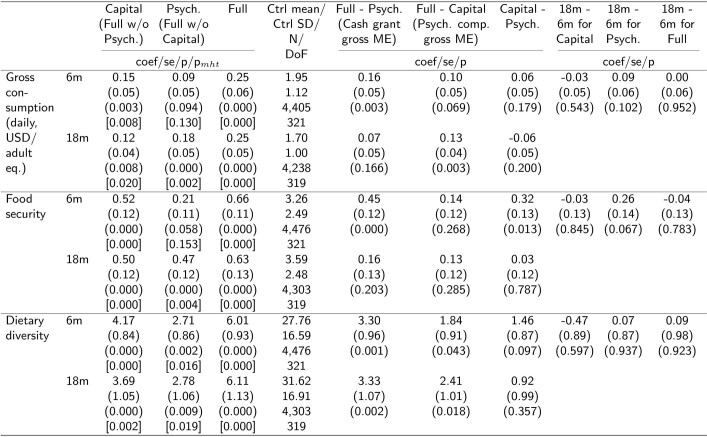
Consumption and Food Security

Notes: Results presented are OLS estimates that include controls for randomization strata and, where possible, baseline outcomes. We assign baseline strata means to households surveyed at midpoint or endpoint but not at baseline and we control for such missing values with an indicator. See Table SI.3 for details on variable construction. Robust standard errors are shown in parentheses, clustered at the village level. Two-tailed p-values are also shown in parentheses, followed by MHT-adjusted p-values shown in square brackets (see table SI.5 for correction details). All monetary amounts are PPP-adjusted USD terms, set at 2016 prices and deflated using Niger CPI published by the World Bank. In 2016, 1 USD = 242.553 XOF PPP. All continuous variables are winsorized at the 98th and 2th percentiles at the most disaggregated level feasible.

**Extended Data Table 2:**
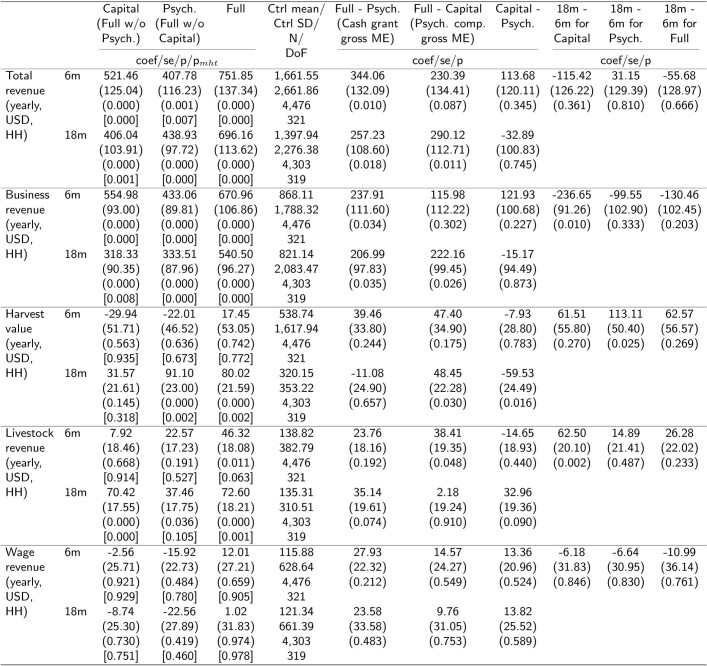
Household Revenues

Notes: Results presented are OLS estimates that include controls for randomization strata and, where possible, baseline outcomes. We assign baseline strata means to households surveyed at midpoint or endpoint but not at baseline and we control for such missing values with an indicator. See Table SI.3 for details on variable construction. Robust standard errors are shown in parentheses, clustered at the village level. Two-tailed p-values are also shown in parentheses, followed by MHT-adjusted p-values shown in square brackets (see table SI.5 for correction details). All monetary amounts are PPP-adjusted USD terms, set at 2016 prices and deflated using Niger CPI published by the World Bank. In 2016, 1 USD = 242.553 XOF PPP. All continuous variables are winsorized at the 98th and 2th percentiles at the most disaggregated level feasible. Wage revenues are wage earnings scaled up by the median monthly profit margin of household businesses.

**Extended Data Table 3:**
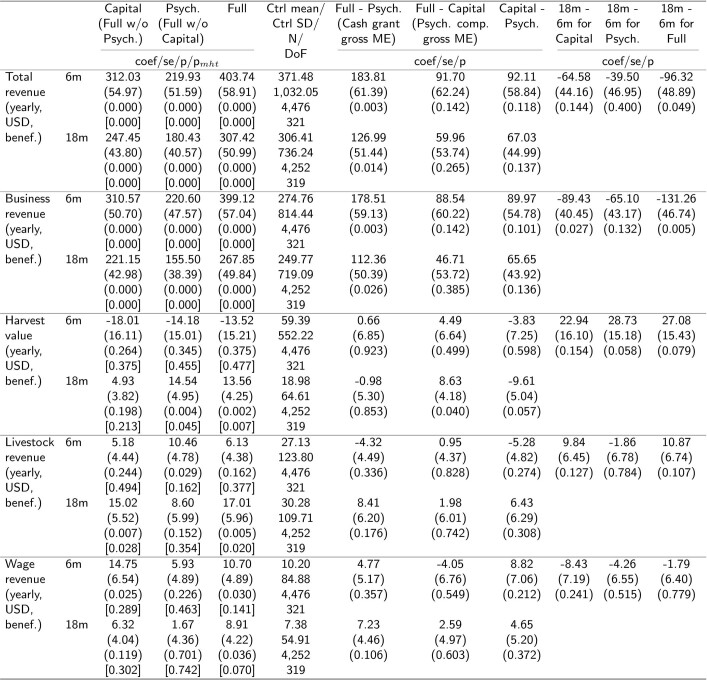
Beneficiary Revenues

Notes: Results presented are OLS estimates that include controls for randomization strata and, where possible, baseline outcomes. We assign baseline strata means to households surveyed at midpoint or endpoint but not at baseline and we control for such missing values with an indicator. See Table SI.3 for details on variable construction. Robust standard errors are shown in parentheses, clustered at the village level. Two-tailed p-values are also shown in parentheses, followed by MHT-adjusted p-values shown in square brackets (see table SI.5 for correction details). All monetary amounts are PPP-adjusted USD terms, set at 2016 prices and deflated using Niger CPI published by the World Bank. In 2016, 1 USD = 242.553 XOF PPP. All continuous variables are winsorized at the 98th and 2th percentiles at the most disaggregated level feasible. Wage revenues are wage earnings scaled up by the median monthly profit margin of household businesses.

**Extended Data Table 4:**
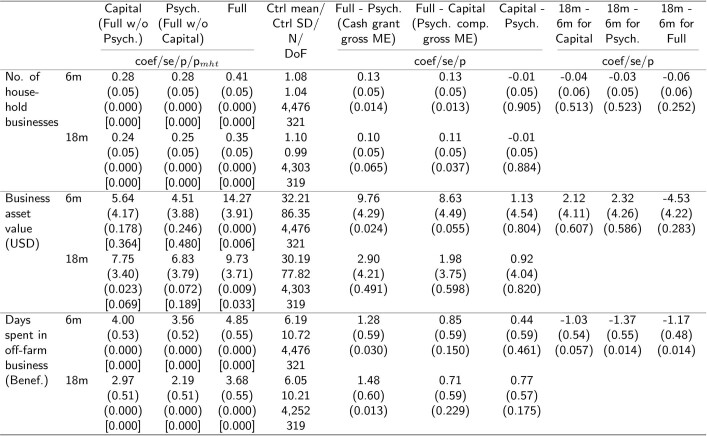
Off-Farm Businesses

Notes: Results presented are OLS estimates that include controls for randomization strata and, where possible, baseline outcomes. We assign baseline strata means to households surveyed at midpoint or endpoint but not at baseline and we control for such missing values with an indicator. See Table SI.3 for details on variable construction. Robust standard errors are shown in parentheses, clustered at the village level. Two-tailed p-values are also shown in parentheses, followed by MHT-adjusted p-values shown in square brackets (see table SI.5 for correction details). All monetary amounts are PPP-adjusted USD terms, set at 2016 prices and deflated using Niger CPI published by the World Bank. In 2016, 1 USD = 242.553 XOF PPP. All continuous variables are winsorized at the 98th and 2th percentiles at the most disaggregated level feasible.

**Extended Data Table 5:**
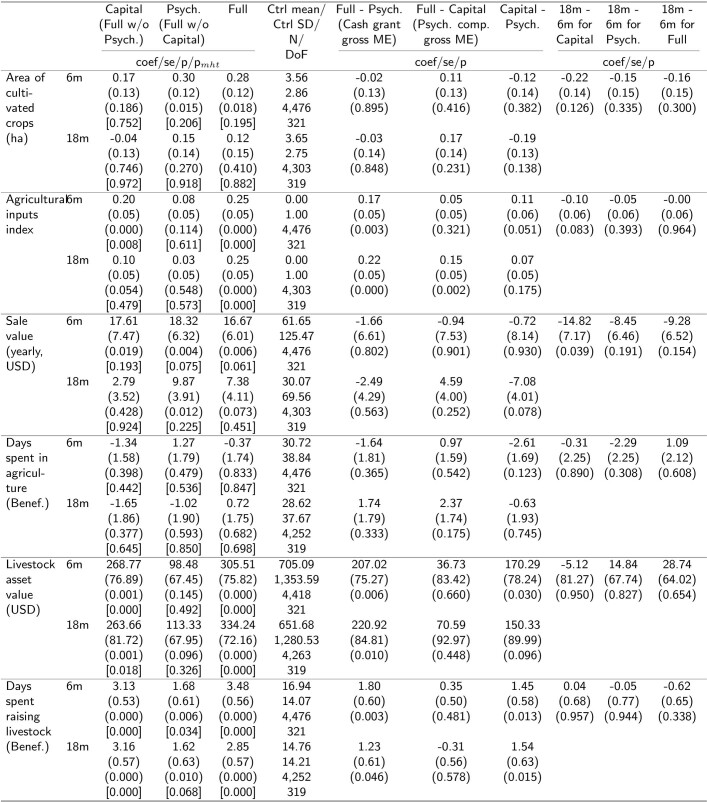
Agriculture and Livestock Activities

Notes: Results presented are OLS estimates that include controls for randomization strata and, where possible, baseline outcomes. We assign baseline strata means to households surveyed at midpoint or endpoint but not at baseline and we control for such missing values with an indicator. See Table SI.3 for details on variable construction. Robust standard errors are shown in parentheses, clustered at the village level. Two-tailed p-values are also shown in parentheses, followed by MHT-adjusted p-values shown in square brackets (see table SI.5 for correction details). All monetary amounts are PPP-adjusted USD terms, set at 2016 prices and deflated using Niger CPI published by the World Bank. In 2016, 1 USD = 242.553 XOF PPP. All continuous variables are winsorized at the 98th and 2th percentiles at the most disaggregated level feasible.

**Extended Data Table 6:**
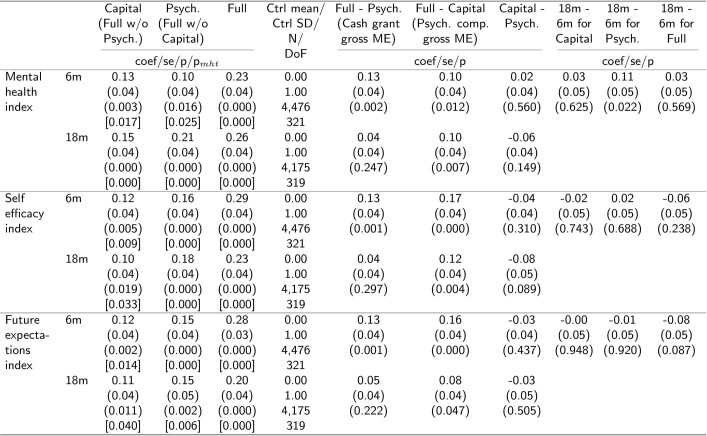
Psychological Well-Being Indices

Notes: Results presented are OLS estimates that include controls for randomization strata and, where possible, baseline outcomes. We assign baseline strata means to households surveyed at midpoint or endpoint but not at baseline and we control for such missing values with an indicator. See Table SI.4 for details on variable construction. Robust standard errors are shown in parentheses, clustered at the village level. Two-tailed p-values are also shown in parentheses, followed by MHT-adjusted p-values shown in square brackets (see table SI.5 for correction details). All indices are standardized with respect to the control group in that survey round. Results from components of each index are provided in Table SI.14 (mental health index components), Table SI.15 (self-efficacy index components), and Table SI.16 (future expectation index components).

**Extended Data Table 7:**
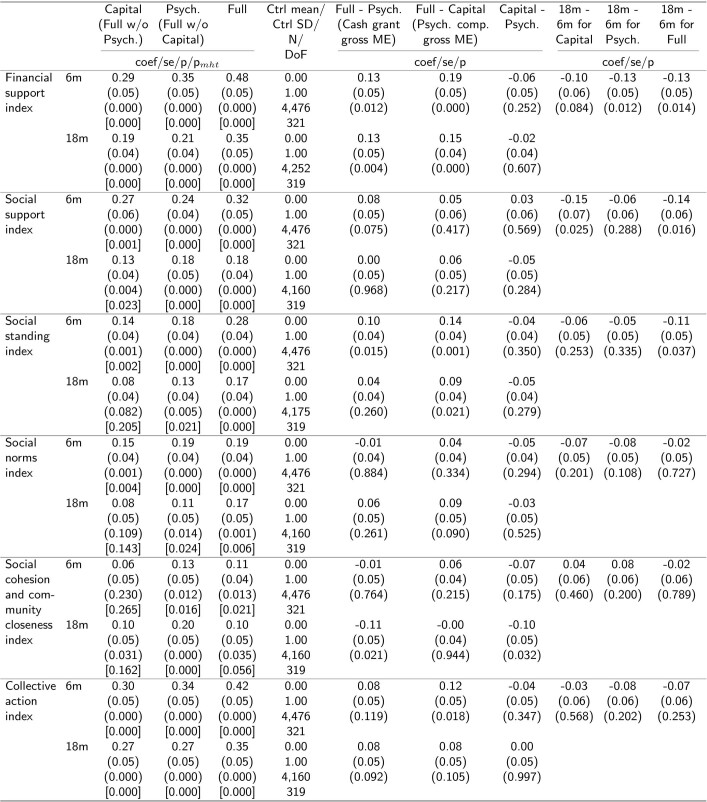
Social Well-Being Indices

Notes: Results presented are OLS estimates that include controls for randomization strata and, where possible, baseline outcomes. We assign baseline strata means to households surveyed at midpoint or endpoint but not at baseline and we control for such missing values with an indicator. See Table SI.4 for details on variable construction. Robust standard errors are shown in parentheses, clustered at the village level. Two-tailed p-values are also shown in parentheses, followed by MHT-adjusted p-values shown in square brackets (see table SI.5 for correction details). All indices are standardized with respect to the control group in that survey round. Results from components of each index are provided in Table SI.17 (financial support index), Table SI.18 (social support index), Table SI.19 (social standing index), Table SI.20 (social norms index), Table SI.21 (social cohesion and community closeness), and Table SI.22 (collective action index).

**Extended Data Table 8:**
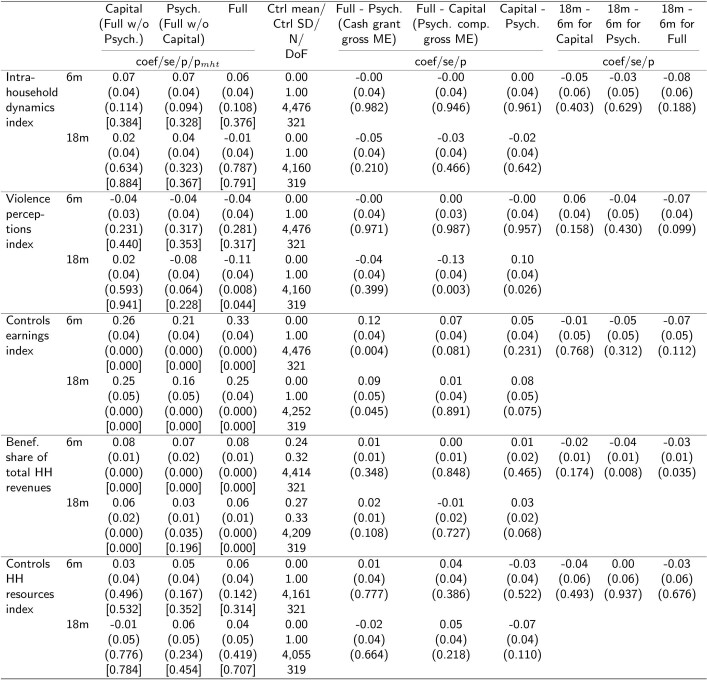
Women’s Control Over Earnings and Household Decision-Making

Notes: Results presented are OLS estimates that include controls for randomization strata and, where possible, baseline outcomes. We assign baseline strata means to households surveyed at midpoint or endpoint but not at baseline and we control for such missing values with an indicator. See Table SI.4 for details on variable construction. Robust standard errors are shown in parentheses, clustered at the village level. Two-tailed p-values are also shown in parentheses, followed by MHT-adjusted p-values shown in square brackets (see table SI.5 for correction details). All indices are standardized with respect to the control group in that survey round. Results from components of each index can be found in Table SI.23 (intra-household dynamics), Table SI.24 (violence perceptions), Table SI.25 (control over earnings and productive agency), and Table SI.26 (control over household resources).

**Extended Data Table 9:**
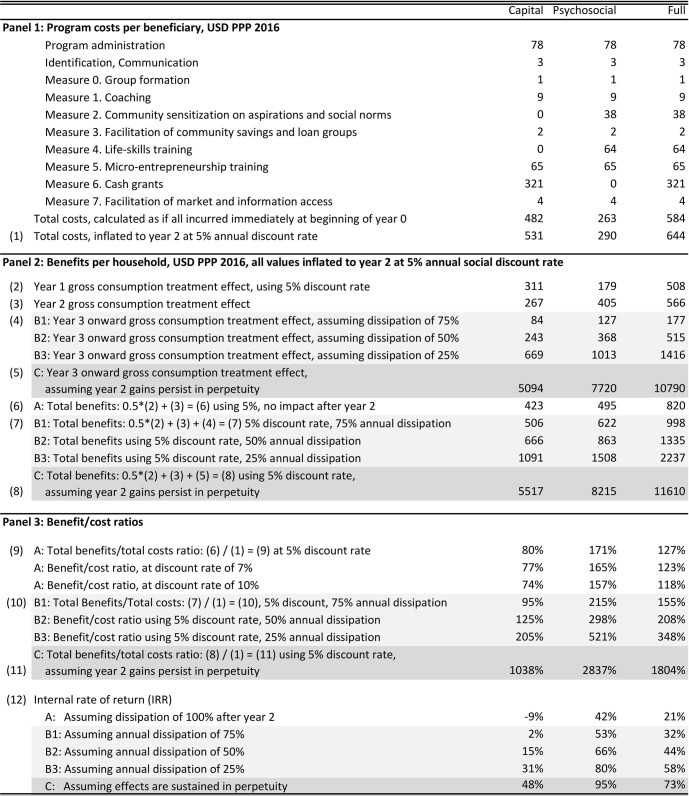
Cost-benefit Analysis

Notes: We use 98% winsorized consumption estimates in our benefits calculation. Note that in rows 6, 7, and 8 we halve consumption benefits in year 1 since we measure them 6 months after the intervention on average. We assume yearly impacts post-intervention. We show results assuming linear growth of impacts by month in Supplementary Table SI.27. All monetary amounts are PPP-adjusted USD terms, set at 2016 prices and deflated using Niger CPI published by the World Bank. In 2016, 1 USD = 242.553 XOF PPP. In nominal terms, in 2016, 1 US dollar = 592.445 XOF. We consider the costs to be incurred in 2018, with an inflation rate of 5.85%. Hence 10,000 XOF in 2018 = 10000 / (592.4 * 1.0585) = $15.95 in 2016. Differences in benefit-cost ratios (row 9) between Psychosocial and Full: 1.709-1.275=0.434, s.e=0.432, p=0.315; between Capital and Full: 0.796-1.275=-0.479, s.e.=0.221, p=0.030; and between Capital and Psychosocial: 0.796-1.709=-0.913, s.e=0.418, p=0.029. Consistent results are found using other scenarios.

## Data Availability

The data used in this paper are available at https://microdata.worldbank.org/index.php/catalog/4294.

## Code availability

The code needed to reproduce the results is available at: https://github.com/dime-worldbank/niger-asp-reprod.git. DIME analytics has verified the computational reproducibility of the results.
