# Supplementary material for: Tackling psychosocial and capital constraints to alleviate poverty
Source: Nature. 2022 Apr 27;605(7909):291–7. doi: 10.1038/s41586-022-04647-8 (PMC9095470; doi:10.1038/s41586-022-04647-8)
Supplement: Supplementary file 1 — This file contains Supplementary Notes, Supplementary Fig. 1, and Supplementary Tables 1–30. Supplementary Notes: Part 1 details the implementation of the government-led national cash transfer programme. Parts 2, 3 and 4 provide additional information on the psychosocial components and outcomes and describes deviations from the pre-analysis plan. Supplementary Figures: Shows a map of communes in the study sample. Supplementary Tables: Details: (1) balance and attrition; (2) compliance; (3) variable definitions and construction notes; (4) multiple hypothesis test corrections, (5) extended results (economic outcomes, spillover mediators, and index subcomponents), (6) cost-benefit calculations, and (7) heterogeneity results. [file 41586_2022_4647_MOESM1_ESM.pdf]

nature portfolio

https://doi.org/10.1038/s41586-022-04647-8

Supplementary information

Tackling psychosocial and capital constraints to alleviate poverty

In the format provided by the authors and unedited

nature portfolio

https://doi.org/10.1038/s41586-022-04647-8

Supplementary information

Tackling psychosocial and capital constraints to alleviate poverty

In the format provided by the authors and unedited

Supplementary Information for "Tackling Psychosocial and Capital Constraints to alleviate Poverty"

March 11, 2022

Contents

1 Government-led program implementation **3**

2 Additional information on the psychosocial components **5**

3 Additional information on the psychosocial outcomes **8**

4 Deviations from the Pre-Analysis Plan **10**

5 Supplementary Figures **11**

Supplementary Fig. 1: Communes in the sample . . . 11

6 Supplementary Tables **12**

1 Supplementary Table SI.1: Balance and Attrition . . . 12
2 Supplementary Table SI.2: Compliance Based on Administrative Data . . . 14
3 Supplementary Table SI.3: Variable Definitions and Construction . . . 15
4 Supplementary Table SI.4: Index Definitions and Construction . . . 21
5 Supplementary Table SI.5: Multiple Hypothesis Test Corrections . . . 28
6 Supplementary Table SI.6: Off-Farm Activities (Household) . . . 31
7 Supplementary Table SI.7: Agriculture (Household) . . . 32
8 Supplementary Table SI.8: Livestock (Household) . . . 34
9 Supplementary Table SI.9a: Labor Participation (Household) . . . 35
10 Supplementary Table SI.9b: Labor Participation (Beneficiary) . . . 36
11 Supplementary Table SI.10a: Financial Engagement . . . 37
12 Supplementary Table SI.10b: Financial Engagement (Extensive Margins) . . . 38
13 Supplementary Table SI.11: Assets (Household) . . . 39
14 Supplementary Table SI.12: Potential Mediators of Spill-Over Effects . . . 40
15 Supplementary Table SI.13: Food Prices (Log Median Village Price, Weighted by Purchase Frequency) . . . 41
16 Supplementary Table SI.14: Mental Health Index Components . . . 42
17 Supplementary Table SI.15: Self-Efficacy Index Components . . . 43
18 Supplementary Table SI.16: Future Expectation Index Components (kids under 30) . . . 44
19 Supplementary Table SI.17: Financial Support Index Components . . . 45
20 Supplementary Table SI.18: Social Support Index Components . . . 46
21 Supplementary Table SI.19: Social Standing Index Components . . . 47
22 Supplementary Table SI.20: Social Norms Index Components . . . 48
23 Supplementary Table SI.21: Social Cohesion and Community Closeness Index Components . . . 50

24 Supplementary Table SI.22: Collective Action Index Components 51
25 Supplementary Table SI.23: Intra-Household Dynamics Index Components 52
26 Supplementary Table SI.24: Violence Perceptions Index Components 53
27 Supplementary Table SI.25: Control Over Earnings and Productive Agency Index Components . 54
28 Supplementary Table SI.26: Control Over Household Resources Index Components 56
29 Supplementary Table SI.27: Cost-benefit analysis (assuming linear growth post-intervention) . . 58
30 Supplementary Table SI.28: Costs and Psychosocial Effects 59
31 Supplementary Table SI.29: Heterogeneity by Baseline Consumption 60
32 Supplementary Table SI.30: Heterogeneity by Baseline Mental Health Index 61

1 Government-led program implementation

The Niger national cash transfer program was put in place through an Adaptive Social Safety Net Project managed by the Safety Net Unit (Cellule Filets Sociaux, CFS) in the Office of the Prime Minister, with support from the World Bank. The government-led program was rolled out in 3 main phases and reached 100,000 beneficiary households between 2012 and 2019. As such, it reached 1 million individuals out of a rural population in poverty of 7 million [1].

Public lotteries were used to select villages for the cash transfer program within targeted communes. This was done for transparency due to the lack of disaggregated data on poverty within communes and the impossibility of covering all villages due to budget constraints. Premand & Barry [2] discuss the process of selecting beneficiary villages through lotteries in more detail for an earlier phase of the program. We do not focus on this aspect as our experiment contains cash transfer beneficiary villages only.

We studied the 3rd phase of the program, implemented from 2016 to 2019, which reached approximately 20,000 households. Three alternative targeting methods were tested and randomized at the village level in the sample used for this study, including proxy means testing, community-based targeting, and a formula to proxy temporary food insecurity. Premand & Schnitzer [3] analyze the relative performance of these targeting methods. The targeting resulted in the selection of approximately 40% of households per village to participate in the program. Note that the program eligibility threshold was below the poverty line, and can be considered closer to an extreme poverty line. About 80 percent of households were below the poverty line in program areas, and approximately the poorest half of them was covered by the program. The program generally provided transfers to the first wife of the household head, though in the context of the study the individual recipient varied among adult women within the households.

The national cash transfer program provided monthly payments of 10,000 XOF for two years ($15.95, $38.95 PPP). The cash transfers were unconditional but were delivered with parenting and child development promotion activities that encouraged investments in young children's human capital for all households in our sample. Premand & Barry [2] disentangle the effects of these parenting and child development promotion activities from cash transfers as part of an RCT embedded in an earlier phase of the program between 2012 and 2014.

The multi-faceted economic inclusion intervention was designed for implementation through national cash transfer programs across the Sahel. The system to deliver the intervention varies across countries, ranging from a fully government-implemented program to a fully NGO-implemented program [4]. The Niger intervention stands out as being closely integrated with a government-led national cash transfer program.[3] This makes it particularly informative about the potential for effective scale-up of multi-faceted interventions through government systems.

National, regional and local staff from the Niger national cash transfer program are responsible for key aspects of implementation, actively leading the delivery of the savings groups, coaching, access to market and cash grant components. Field agents lead the selection, training and supervision of community coaches. Field agents are also in charge of coordinating and supervising the delivery of trainings contracted out to private providers (through small firms). The role of private providers is limited to the hiring of short-term qualified personnel, which are trained by the program to deliver the training content and curriculum. The cash grant was delivered through the cash transfer program payment system, which relied on a network of micro-finance institutions that deliver cash in person.

Coaches were not cash transfer beneficiaries. They were selected by the community in an open assembly. They received 10,000 XOF per month (11% of consumption for beneficiary household, less for coaches' households),

[3] The institutional anchoring of the program in a high-level government structure is also noteworthy. The national cash transfer program is led by a safety nets unit in the Office of the Prime Minster. From a political economy standpoint, governments in Sub-Saharan Africa (including Niger) find the "productive" dimension of social protection programs particularly valuable. As such, the intervention's goal to strengthen productive impacts among rural populations is closely aligned with higher-level policy objectives.

plus small contributions from beneficiaries. This was considered a stipend and not a salary. These community coaches had a few years of education and limited technical skills, but were chosen by the community for their trustworthiness and knowledge about local economic opportunities. They typically had basic literacy skills, and in many cases were younger men who had completed primary school and did not have stable employment.

The productive inclusion intervention is designed to be low-cost and scalable. Costs are kept low by layering the intervention on existing delivery systems of the cash transfer program, leveraging pre-existing targeting efforts, beneficiary registries, or monitoring and information tools. Field implementation is facilitated by working with an existing structure of staff at the local, regional and national levels. The delivery model is designed to ensure feasibility of implementation at scale. A key parameter is the number of program staff per beneficiary.[4] In the context of the government-led program, there is 1 program staff per 8.8 villages (covering 596 beneficiaries, or 25 beneficiary groups). This is a much lower ratio of staff per beneficiary than for standard NGO programs. In contrast, the model relies much more heavily on community coaches. There are on average 1.2 coaches per village (large villages had 2). Each coach is responsible for an average of 56 beneficiaries or 2.4 groups. While the reliance on lower-level agents may reduce quality of implementation, it lowers cost and is a more realistic model for implementation at a large scale.[5] Lastly, the lump-sum cash grant is delivered through the existing cash transfer program payment system. This was considered easier to implement than the in-kind asset transfers provided by many traditional graduation programs.[6]

In light of these implementation modalities, the results can be interpreted as those obtained when delivering a well-designed and highly scalable intervention through a relatively high-performing government implementing agency in a very poor country.

[4] Program staff (field agents) were hired by the national safety nets unit for the cash transfer program (which started in 2011). They do assignments of 2 years (the duration of a cash transfer cycle), typically before rotating to a new area. They are based in the mayor's office in each commune. In communes where the productive interventions were implemented, there were around 30 field agents. Nationally, the total number of field agents working in the cash transfer program is around 100. Field agents are supervised by regional safety net offices (of which there are 8), with dedicated staff. Regional offices then report to the central safety net unit in the prime minister's office.

[5] In the case of the Niger cash transfer program, the approach is a routine way of delivering services and has also been used to implement accompanying measures to promote child development at large scale [2].

[6] While the program is largely government-led and government-implemented, it was funded by the World Bank. This made it possible to use streamlined financial management procedures and hire program staff on term contracts. Although the program staff had a profile similar to other government technical agents, they likely had stronger performance incentives. The World Bank and international NGOs provided technical support for the design and monitoring of the intervention, but the government agency was fully responsible for delivery and implementation.

2 Additional information on the psychosocial components

The psychosocial components included a community-wide sensitization and a life skills training. These were designed to simultaneously address multiple psychosocial constraints to beneficiaries' economic engagement, from individual- to community-level factors, and to do so in culturally responsive ways. We posited that sustained positive impacts on women's economic outcomes would depend not only on women building their skills but also on having instrumental and normative support from their household and community. Thus, in addition to life skills trainings for beneficiaries, we added the innovation of light-touch community programming—a community-wide film screening and discussion that targeted social norms and collective aspirations and that preceded other intervention components.

These design choices build on two foundational ideas in social and cultural psychology. First, beyond individual motivation, behavior is influenced by what the social context permits [5, 6]. Accordingly, individual-level behavior change strategies, like life skills trainings, are expected to have greater and more sustained effects when, for instance, social norms are also supportive of the new behaviors [7]. Second, cultures across the world display different levels of collectivism and social norm enforcement, which shape individual behaviors [8–11]. Hausa and Zarma ethnic groups in Niger are predominantly Muslim, highly collectivistic, and gender roles are guided by strong social norms and religious practices.[1] In such normatively tight and interdependent sociocultural contexts, coordinating individuals' behavior and goals with those of their community and aligning the interventions with commonly-held values was expected to lead both to greater individual motivation and a more supportive social support context [12, 13].

1. Community sensitization on aspirations and social norms

The sensitization aimed to introduce the productive interventions' objectives to the community and to promote social norms and aspirations supportive of women's economic activities.

Content of the intervention. The sensitization was an evening program in which beneficiaries and other family and community members were invited to gather to view a film projected on a large screen, followed by a guided group discussion. The activity lasted approximately 1.5 hours in total.

The 20-minute film showed an aspirational story that combined local stories collected during qualitative piloting with social and behavior change principles. It was recorded in Hausa and Zarma.[2] The film portrayed the story of a Muslim woman named Amina pursuing a new business (processing and selling hibiscus or "bissap" juice) after a drought affected her millet crop. Amina is shown using the support of her family and community to adapt her economic activities, including consulting her mother-in-law on adaptation and change, her cousin on making bissap juice, and her husband on their financial options. She and her husband overcome a conflict on her plan to save with her women's group, with the father-in-law being the mediator. In the end, she and her husband are shown selling bissap juice, food, and solar-based charging in their new shop as their children return from school. In the final scene, Amina shares what she has learned with other women in the village.

Immediately after the film, the audience was led through a group discussion of approximately 40-60 minutes. There were five discussion topics: summarizing the film's main themes; relating the film to the audience's experiences; sharing family and community stories about 'traditions of adaptation'; identifying best practices for more secure future; and creating consensus around solidarity toward this future. Questions prompted the community to relate the film's storyline and characters to their lives; to relate adaptation to traditional communal values and practices, including filial piety, security for future generations, and reciprocity; and to collectively set aspirations and identify practices for economic

[1] For instance, see Hofstede Insights Country Comparison (www.hofstede-insights.com/country-comparison).

[2] See links to full video in Zarma and Hausa with French subtitles, and 4-minute clip with English subtitles.

advancement. Facilitators were encouraged to pick respondents from across the audience to elicit widespread participation and to elevate women's responses.

Aligning desired behaviors with communal values. Given strong social and religious norms in rural Niger, in certain areas women's economic engagement can lead to social censure, particularly if women are seen to be self-interested and ambitious. Some interventions in the international literature actively teach self-focus and personal initiative skills to improve outcomes of income-generating activities [14]. However, the approach here considers a more interdependent model that recognizes the motivational power of matching a group's values and cultural ways of being, as opposed to overlooking or overriding them. The community sensitization thus sought to frame the goals of the economic inclusion interventions and women's economic activity as being aligned with binding, communal values, such as solidarity, filial piety, loyalty, and respect for authority [10, 15–18]. For instance, in the film Amina demonstrates her interdependent goals to help her children, her husband, and her community through her economic activities, beyond her own advancement, and she shows solidarity and loyalty to her peers by sharing her new knowledge with them. Amina shows filial piety and respect for authority by seeking out advice and mediation from her elders, who provide supportive advice based on their own experience adapting to changing circumstances in the past. Relatedly, the discussion asked the community to reflect on 'traditions of adaptation' from their ancestors so that upcoming changes in economic practices could be understood to represent, rather than defy, tradition and filial piety.

Community participation and social norms. Particularly in this more interdependent and normatively tight sociocultural study context, watching the film with close others was expected to better shift social norms around typical and appropriate economic behaviors for women and to generate more sustained social support [19, 20]. Accordingly, beneficiaries were encouraged to invite their husbands, family members, and friends. Given the importance of hierarchy and of local leaders in norm setting and enforcement [21, 22], traditional leaders were engaged in conducting the event and, along with other elders, religious leaders, and economic leaders, were invited to attend. Leveraging group-based decision-making for social change [6], the discussion prompted the audience to build consensus around practicing norms of mutual support and solidarity, particularly of the beneficiaries, that could advance community development.

Role models, aspirations, and goal setting. A goal of role modeling women's economic engagement through film was to build self-efficacy and promote aspirations for economic mobility both among beneficiaries and their community. According to social cognitive theory, the use of role models can encourage uptake of new behaviors via observational learning of social exemplars; it can also build self-efficacy through vicarious experience, which can in turn boost aspirations, motivation, and resilience to setbacks [19]. Because similarity and relatability of characters are central to narrative persuasion, the video involved local actors and the discussion prompted the audience to connect the film's story to their own lives [23]. The film also featured traditional music and proverbs in Hausa and Zarma.

Building on an evidence-based motivational strategy of WOOP, or Wish Outcome Obstacle Plan [24], the discussion further prompted participants to project themselves into the future, to consider the goal of creating a more secure future for the next generation in particular, and then to identify adaptation strategies that could advance this goal as well as difficulties that could stand in the way. The discussion leader closed by expressing positive expectations of a strong future for the community.

2. Life Skills Training

The life skills training was delivered over 7 half-days. It was supported by emerging research on the benefits of socio-emotional or soft skills trainings. Teaching soft skills such as self-worth, interpersonal communication, and sense of control have been shown to promote a range of positive life outcomes, including improved productivity, earnings, health, and educational outcomes [25–29]. Several studies

have found soft skills trainings to be as or more effective in boosting employment and entrepreneurial outcomes compared to hard skills or technical trainings [14, 30].

The training was grounded in 'andragogy,' an approach to adult education that prioritizes self-directed, experiential, problem-centered, participatory, and personalized learning [31]. The training thus incorporated individual- and group-based exercises, including collective brainstorming sessions, case studies, role plays, games, open discussions, and illustrations.

The training included 9 modules:

1. Overview of the life skills curriculum, key competencies, and their utility for achieving one's aspirations and realizing one's values.
2. Our values and perspectives: Identification of one's three top communal and individual values (e.g., honor, dignity, generosity, loyalty, piety, education, family); understanding how one can exercise these values through their work.
3. Self-affirmation and self-worth: Identifying one's strengths, value to one's family and others, and role in society; linking self-worth to personal initiative and self-expression as well as to openness to feedback and change; understanding differences between self-worth and selfishness, aggressiveness, or passiveness.
4. Decision-making and problem solving: How to identify a problem; analyze relevant information; gather different perspectives; identify multiple solutions; anticipate consequences; make informed decisions that meet one's values, vision, and the interests of concerned persons; act on the decision; and reflect on the outcomes.
5. Second viewing of the sensitization film: Reinforcement of key themes and competencies displayed in Amina's story, including her values and decision-making, creativity in the face of shocks, leading by example, and contributions to her community's development.
6. Interpersonal communication and using one's voice: Understanding the significance of good communication, common obstacles, and key principles of good communication and self-expression (e.g., using a message clear and simple, credible information, productive circumstances and channels of communication, an approach tailored to the relationship and status of the other person, maintained eye contact, audible and slow speech, amicable and open verbal and non-verbal communication).
7. Financial education: Setting and prioritizing top financial goals and charting out intermediate steps; mental contrasting with future savings, building a culture of saving, defining one's capacity to save, and seeing savings as a source of resilience; budgeting across seasons and across expense types.
8. Leadership and gender: Identifying female leaders and role models and qualities of leaders (e.g., having strong networks; taking responsibility; mediating conflicts; being forward-looking, inspiring, calm, and courageous); discussing ways women can contribute to community decision-making and support their communities and families; and brainstorming ideas for actions that participants could take in their communities.
9. Third viewing of the sensitization film and summary: Analysis of the film in relation to the competencies displayed by different actors; summary of the session including an exchange of participants' experiences and lessons learned.

3 Additional information on the psychosocial outcomes

Process of measure selection, adaptation, and development

Through piloting, the research team selected and adapted items from existing scales as well as developed original measures with face validity, or subjective construct accuracy in the local context. We additionally included culturally meaningful indicators of psychological and social well-being (e.g., inner peace, enemyship). In the selection and development of psychosocial measures, careful attention was paid to cultural relevance and comprehensibility. All items, and their references where relevant, are described in Supplementary Table SI.4. Internal consistency, or reliability, for each index is presented below from the control arm data at the 1st follow-up.[7]

Psychological well-being indices

The psychological well-being outcomes assessed mental health and psychosocial assets, including self-efficacy and future expectations. Broadly, mental health and psychosocial assets predict a range of positive economic, health, and empowerment outcomes [34, 35]. In particular, poor mental health, self-efficacy, and hope have been posited to contribute to poverty through their effects on motivation and future-oriented decision-making [35–38].

Mental health (17 items, $\alpha = .82$) included CESD-R-10, a depression screener designed for community samples; functional disability items from SRQ-20; life satisfaction using an adapted Cantril ladder; sense of inner peace; and self-assessed mental health. Disability items, assessing somatic symptoms and role functioning, and self-assessed mental health item were included to capture cultural differences in mental illness symptoms (e.g., somatization) and functional impairments [39, 40]. Inner peace was considered a culturally specific indicator of well-being, according to qualitative piloting and West African studies [41].

Self-efficacy (8 items, $\alpha = .76$) captured judgments of one's capabilities, specifically in relation to problem solving, goal pursuit, and coping, and a related self-esteem question. Self-efficacy has been found to be a motivational keystone, particularly of goal setting and pursuit [42]. It has been theorized as a driver of the development of social, human, and financial capital and thus of economic development broadly [35].

Future expectations (3 items, $\alpha = .76$) gauged expectations for personal and intergenerational socioeconomic status as well as life satisfaction in the future, through adapted MacArthur ladders. Notably, hope, or positive expectations for the future, has been posited to contribute to graduation programs' effects on poverty reduction [37]. Additionally, Delavande *et al.* [43] find that subjective expectations often accurately predict future behaviors and outcomes and, in this case, capture expected trajectories of mobility.

Social well-being indices

The social well-being outcomes were seen as potential determinants of women's economic outcomes in the low literacy, low resource, and normatively tight study setting where women's opportunities often come through their relationships and where reciprocal networks of support can be critical to cope with shocks [44, 45]. Moreover, social well-being is often integral to individual happiness and wellbeing among interdependent groups [41, 46].

Financial support (3 items, $\alpha = .26$) and *social support* (6 items, $\alpha = .66$) assessed women's level of social capital [44]. Financial support assessed the extent of one's financial support network, based on perceived ability to receive financial help in times of need and number of financial supporters. Social support assessed the extent of one's instrumental support network, based on the number of relationships one has for acquiring information, advice, and opportunities. Together, these questions capture whether beneficiaries are able to develop informal systems of support for economic resilience and opportunity.

[7]Note that, while Cronbach's alpha values for some indices here would be considered low in Western samples, these results are comparable to other studies which find that psychological measures have lower consistency in low-income countries, in sub-Saharan Africa specifically, than in the West [32, 33].

Social standing (4 items, $\alpha = .75$) assessed the MacArthur socioeconomic status ladder and three context-specific ladders of community standing: being respected, having one's opinion followed, and showing moral behavior. Such self-assessments of one's social position have been found to predict health, well-being, and feelings of financial security, above and beyond income [34].

Social norms (8 items, $\alpha =.55$) assessed descriptive and prescriptive norms supportive of women's economic engagement. The *descriptive norms* sub-index (4 items, $\alpha = .55$) assessed perceptions of other women in the village engaging in economic activities, such as starting new activities, becoming vendors, and traveling outside the village. The *prescriptive norms* sub-index (4 items, $\alpha = .61$) assessed perceptions of other men and women believing that women should engage in, i.e., social approval versus censure of, such activities.

Social cohesion and community closeness (9 items, $\alpha = .47$) assessed social interdependence and collectivism, expectations of social support, feelings of trust and closeness, experienced tension (reversed), and number of enemies (reversed). Interdependence and collectivism items measure how individuals view themselves in relation to others, i.e., as similar, connected, and responsive versus separate, unique, and autonomous; they reflect culturally-specific forms of motivation that were integrated into the design of the psychosocial interventions. Additionally, the number of enemies, or people who wish to sabotage your success, was included as a locally relevant indicator of social cohesion [47], particularly given that enemies can arise from envy and resentment following inequalities in new economic opportunities or resources.

Collective action (5 items, $\alpha = .34$) assessed community engagement and support through the number of groups belonged to, the number of leadership positions held, monetary and volunteer contributions to community projects, and self-reported collective initiative. This measure assessed women's engagement in community leadership as well as the potential of the interventions to create indirect benefits to communities.

4 Deviations from the Pre-Analysis Plan

We deviate slightly from the deposited pre-analysis plan when constructing outcomes and grouping them into tables. Multiple hypothesis testing is shown in Supplementary Table SI.5 on the original defined outcome families.[1] The changes in construction of the economic variable outcomes were made only to improve interpretability or comparison and are generally related to scaling. As we document below, we also add some additional outcomes that were not pre-specified, but think the reader will expect.

In Extended Data Table 1, for ease of interpretability, we show a reversed food insecurity scale (sum of food insecurity experiences) instead of the Rasch model with severity weights. We also include the beneficiary's dietary diversity as a primary outcome.

In Extended Data Tables 2 and 3, we show the beneficiary's productive revenues, and include the same outcomes for the household for comparison. We scale all revenues to 12 months and show a calculated total revenue. For ease of exposition, Extended Data Tables 4 and 5 combine pre-defined outcomes from several pre-specified families into a new family and includes a newly defined agricultural inputs index.

For the indices in Extended Data Tables 6-8, we document a few deviations from the pre-analysis plan. The two components of the "self and social worth index" were analyzed as separate indices given loadings on separate factors in exploratory factor analysis and for interpretability. The "social trust and cohesion index" was revised to focus exclusively on community relations, including four collectivism items, while remaining household related items, alongside items of the "relationship quality index," were moved to an "intra-household dynamics index" for finer comparisons of social dynamics within and outside the household. One item from the "household relations index" and all items from the "productive activities index" were combined into a "social norms index" given their common question structure and content (i.e., related to women's productive activities). The "productive agency index" and items from the "decision weight index" related to women's earnings and businesses were combined into a single "control over earnings and productive agency index" while remaining items from the "decision weight index" and "decision possibility index" were combined into a "control over household resources index" in order to distinguish patterns across women's personal affairs and household affairs.

We expand the pre-specified family of non-agricultural activity outcomes (Supplementary Table SI.6) by adding the beneficiary's count of good business practices and an indicator for whether a beneficiary's main activity is off-farm. To agricultural outcomes (Supplementary Table SI.7), we add an agricultural inputs index which was not pre-specified. To livestock outcomes (Supplementary Table SI.8), we add sale revenue, TLU, and total value which were not pre-specified. For labor participation (Supplementary Table SI.9), we include household outcomes following the set-up in Extended Data Table 2–showing results in days rather than hours for ease of comparison across activity types.

In Supplementary Table SI.10a, instead of a household asset count, we present an index, and combine with the family of variables on financial engagement. Supplementary Table SI.11 shows a subset of the variables in the original assets family as some are shown previously.

Additional analysis mentioned in the pre-analysis plan but not discussed in this paper will be reported as part of the multi-country study when data from other sites become available.

[1] In the pre-analysis plan, we indicated we would apply multiple hypothesis corrections on the set of tests that include all individual arm treatment effects on each outcome within an outcome family. Instead, we correct for multiple hypotheses within each treatment arm across the outcomes in each family. We now believe this is sufficient because treatment arms were pre-defined.

5 Supplementary Figures

Supplementary Fig. 1: Communes in the sample

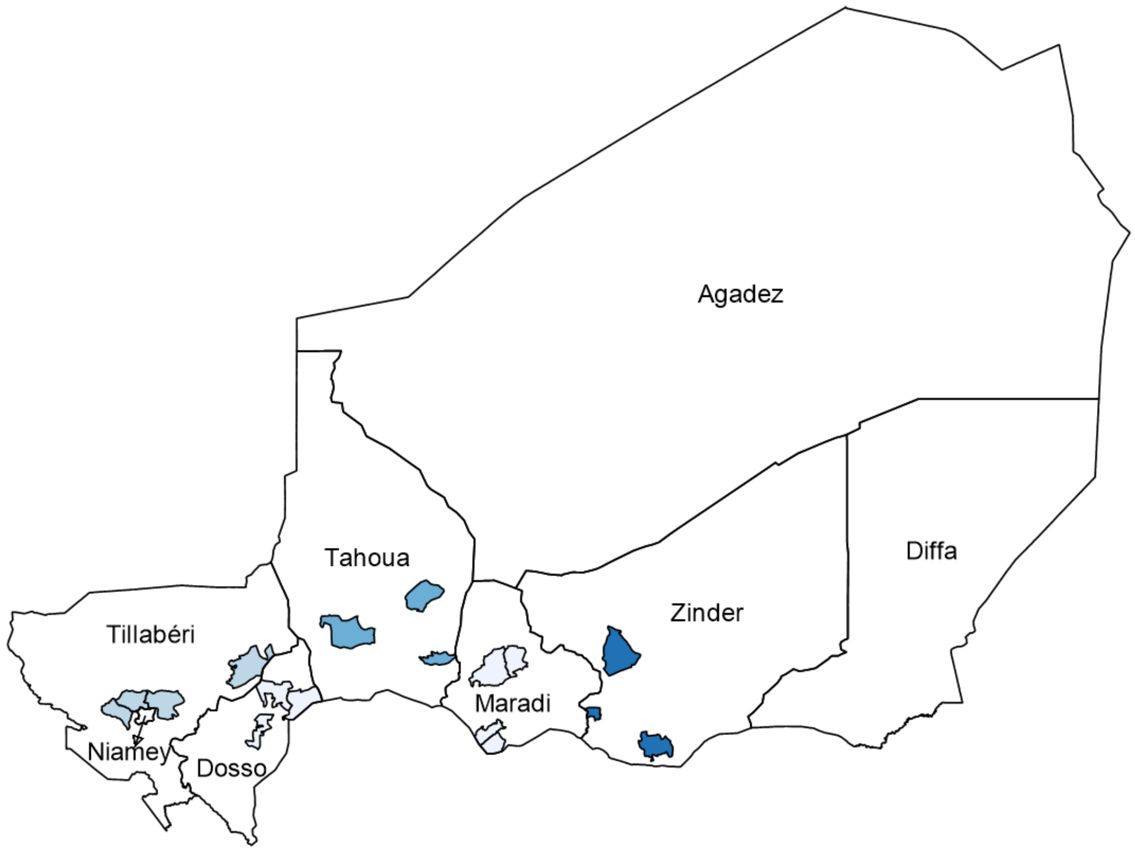

Notes: Authors' creation; boundaries from OCHA Common Operational Data.

6 Supplementary Tables

Supplementary Table SI.1: Balance and Attrition

	Control Mean/SE	Capital Mean/SE	Psychosocial Mean/SE	Full Mean/SE	T-test p-value						Joint F-test p-value	Pooled F-test p-value
Variable	(1)	(2)	(3)	(4)	(1)-(2)	(1)-(3)	(1)-(4)	(2)-(3)	(2)-(4)	(3)-(4)	(5)	(6)
Beneficiary is HH head	0.104	0.109	0.111	0.102	0.423	0.282	0.843	0.714	0.381	0.232	0.660	0.606
	(0.011)	(0.012)	(0.010)	(0.011)								
Beneficiary is handicapped	0.013	0.021	0.015	0.018	0.174	0.632	0.133	0.179	0.569	0.740	0.653	0.178
	(0.003)	(0.004)	(0.004)	(0.004)								
Female (HH head)	0.102	0.116	0.114	0.113	0.135	0.163	0.310	0.977	0.541	0.491	0.883	0.181
	(0.011)	(0.013)	(0.012)	(0.012)								
Female (beneficiary)	0.986	0.990	0.987	0.988	0.326	0.821	0.860	0.514	0.751	0.760	0.839	0.417
	(0.003)	(0.003)	(0.004)	(0.003)								
Polygamy (HH head)	0.285	0.263	0.266	0.300	0.498	0.292	0.300	0.968	0.077*	0.106	0.154	0.918
	(0.018)	(0.015)	(0.015)	(0.018)								
Polygamy (beneficiary)	0.266	0.252	0.255	0.284	0.810	0.508	0.213	0.929	0.117	0.106	0.213	0.772
	(0.017)	(0.015)	(0.014)	(0.017)								
Age (HH head)	47.607	47.201	47.240	46.562	0.496	0.188	0.106	0.853	0.307	0.152	0.468	0.155
	(0.478)	(0.433)	(0.509)	(0.478)								
Age (beneficiary)	37.818	37.971	37.821	37.095	0.412	0.679	0.348	0.314	0.037**	0.106	0.195	0.655
	(0.436)	(0.423)	(0.478)	(0.464)								
Education (years, HH head)	0.537	0.646	0.574	0.505	0.161	0.377	0.931	0.937	0.381	0.655	0.531	0.341
	(0.072)	(0.084)	(0.079)	(0.070)								
Education (years, beneficiary)	0.306	0.406	0.284	0.350	0.100	0.799	0.400	0.086*	0.933	0.252	0.271	0.290
	(0.042)	(0.053)	(0.044)	(0.052)								
Primary education (0/1, HH head)	0.025	0.027	0.030	0.024	0.743	0.120	0.658	0.146	0.771	0.528	0.653	0.349
	(0.005)	(0.006)	(0.006)	(0.005)								
Primary education (0/1, beneficiary)	0.015	0.015	0.010	0.012	0.786	0.201	0.416	0.484	0.899	0.869	0.777	0.468
	(0.004)	(0.004)	(0.003)	(0.003)								
Literate (HH head)	0.264	0.277	0.285	0.263	0.224	0.174	0.894	0.626	0.312	0.381	0.638	0.287
	(0.017)	(0.016)	(0.017)	(0.017)								
Literate (beneficiary)	0.071	0.082	0.071	0.075	0.156	0.963	0.454	0.379	0.814	0.767	0.619	0.431
	(0.007)	(0.010)	(0.009)	(0.010)								
Health index (HH head)	0.000	0.027	0.031	0.034	0.515	0.498	0.329	0.941	0.638	0.761	0.951	0.371
	(0.030)	(0.035)	(0.031)	(0.034)								
Health index (beneficiary)	-0.000	0.035	0.002	0.044	0.348	0.947	0.351	0.293	0.953	0.483	0.657	0.535
	(0.036)	(0.035)	(0.036)	(0.033)								

Continued on next page

Supplementary Table SI.1: Balance and Attrition – continued from previous page

	Control Mean/SE	Capital Mean/SE	Psychosocial Mean/SE	Full Mean/SE	T-test p-value						Joint F-test p-value	Pooled F-test p-value
Variable	(1)	(2)	(3)	(4)	(1)-(2)	(1)-(3)	(1)-(4)	(2)-(3)	(2)-(4)	(3)-(4)	(5)	(6)
No. of rooms in house	2.497	2.422	2.490	2.629	0.577	0.898	0.037**	0.471	0.004***	0.064*	0.019**	0.538
	(0.076)	(0.064)	(0.076)	(0.071)								
Minutes to health center	47.079	46.827	50.488	51.100	0.460	0.400	0.477	0.371	0.447	0.938	0.617	0.702
	(2.707)	(3.579)	(3.448)	(2.733)								
Minutes to market	73.008	68.875	76.453	72.459	0.237	0.416	0.454	0.068*	0.748	0.250	0.271	0.616
	(3.523)	(4.124)	(3.686)	(3.487)								
Minutes to water source	12.143	12.413	12.586	12.652	0.882	0.948	0.494	0.252	0.876	0.387	0.914	0.768
	(1.144)	(1.089)	(1.227)	(0.930)								
Distance to capital of commune (km)	23.402	22.122	23.793	22.128	0.285	0.856	0.077*	0.574	0.975	0.397	0.525	0.415
	(1.200)	(1.102)	(1.294)	(1.140)								
Response rate in baseline sample												
Follow-up 1	0.957	0.953	0.950	0.950	0.420	0.455	0.267	0.991	0.801	0.946	0.991	0.306
	(0.007)	(0.008)	(0.008)	(0.009)								
Follow-up 2	0.910	0.927	0.929	0.907	0.522	0.107	0.445	0.809	0.230	0.148	0.341	0.610
	(0.015)	(0.010)	(0.009)	(0.015)								
N	1188	1165	1084	1171								
Clusters	81	80	78	83								

Notes: Standard errors for all tests are clustered at the village level. Fixed effects using randomization strata are included in all estimation regressions. The joint F-test in column 5 shows the p-value from a test of equality of treatment arms. while the pooled F-test in column 6 shows the p-value from a test of pooled treatment (i.e., a regression with a dummy for any treatment arm). The health index variable is a z-score index standardized against the control group and generated using three physical activity variables: the reported difficulty of (1) lifting a 10 kg bag, (2) walking 4 hours, and (3) working all day in the field. The three components range from 1 to 4. In calculating distance-to-commune, we assign commune centroids to households located more than 30 km away from a centroid that excludes those outlying households in each commune. *** $p < 0.01$, ** $p < 0.05$, * $p < 0.1$.

Supplementary Table SI.2: Compliance Based on Administrative Data

	Treatment Arm			
	Pooled	Capital	Psychosocial	Full
1. Attendance rates of beneficiaries at community savings and loan groups	92.0%	92.8%	91.7%	91.5%
2. Percentage of targeted beneficiaries who received individual coaching each month	52.2%	51.9%	50.5%	54.1%
3. Attendance rates of beneficiaries at micro-entrepreneurship training	95.0%	96.4%	94.9%	93.6%
4. Attendance rates of beneficiaries at community sensitization on aspirations and social norms	89.3%	-	89.0%	89.8%
5. Attendance rates of beneficiaries at life skills training	93.8%	-	94.0%	93.5%
6. Percentage of targeted beneficiaries who received their cash grant	99.9%	99.7%	-	100%

Notes: We show compliance data collected by the program's administrators. Participation in savings groups and coaching sessions is measured at the group level. The individual coaching visits were not designed to reach all households each month. Participation in the community sensitization session (video screening) is measured at the village level. The cash grant provision and participation in training sessions are measured at the individual level.

Supplementary Table SI.3: Variable Definitions and Construction

Variable	Definition
	**Extended Data Table 1 Consumption and Food Security**
Gross consumption (daily, USD/adult eq.)	Total daily consumption per adult equivalent is the sum of daily household food and non-food consumption as well as expenditures on education, healthcare, household repairs, and celebrations, divided by the number of adult equivalents per household. The number of adult equivalents is calculated using the OECD equivalence scale which assigns a value of 1 to the first adult in the household, 0.7 to each additional adult, and 0.5 to each child.<br>For food consumption, we ask about household consumption of a variety of food products in the last week. To get food expenditure, we multiply amounts consumed by prices. For food products that are both purchased and consumed, we use reported purchase prices. For food products that are consumed but not purchased, we use median purchase prices. We winsorize consumed values within each food product and we divide weekly household totals by 7.<br>For non-food consumption, we ask about monthly (and yearly) expenses on a wide variety of goods and services that are typically consumed on a monthly (or yearly) basis. For education expenditures, we ask about spending in the last academic year on transportation and various school-related items such as tuition fees and accommodation. For health expenditures, we ask about monthly and yearly expenses such as doctor's consultations and vaccination costs. For celebrations, we ask about food, non-food, drink, and clothing expenditures made over the last year during events such as marriages or religious ceremonies. For household repair expenditures, we ask about costs incurred over the last year improving or repairing household features such as roofs, walls, or animal pens. For each of these expenditures, we winsorize values at the finest level possible and re-scale to daily values.
Food security scale	Using the FIES scale, we ask eight yes/no questions about a household's food security over the last year where 0 = "yes" and 1 = "no" and we present a raw sum. The questions are "have you or other members of your household 1) been worried about not having enough food, 2) been unable to eat nutritious and healthy foods, 3) had to eat a smaller variety of foods, 4) had to skip a meal, 5) eaten less than they thought they should, 6) run out of food, 7) been hungry but did not eat, and 8) gone an entire day without eating?" [48, 49]
Dietary diversity	We ask beneficiaries about the number of days in the last week that the they consumed items in 8 different food groups, and we compute a linear combination of these items: [50]<br>$2{\cdot}\text{cereals} + 2{\cdot}\text{tubers} + 3{\cdot}\text{pulses} + \text{vegetables} + \text{fruit} + 4{\cdot}\text{meat/fish/eggs} + 4{\cdot}\text{milk} + 0.5{\cdot}\text{oil} + 0.5{\cdot}\text{sugar}$
	**Extended Data Tables 2 and 3 Revenues**
Total revenue (yearly, USD)	Sum of business revenue, harvest value, livestock revenue, and wage revenue.
Business revenue (yearly, USD)	For each business within the household, we ask directly about revenue generated in the last month in which a business was operational, and we ask for the number of operational months in the last year. We also ask who owns, manages, and works in each business. We winsorize revenues at the business-level at the 98th percentile. We exclude businesses that are both owned and managed by someone outside the household. To get yearly amounts, we multiply this monthly revenue by the number of months a business was in operation in the last 12 months. To get the beneficiary's share, we divide revenues by the number of co-owners and we sum only across beneficiary-owned/managed businesses.

Continued on next page

Supplementary Table SI.3 Variable Definitions and Construction – continued from previous page

Variable	Definition
Harvest value (yearly, USD)	To get harvest value, we multiply regional median prices of crops sold by the quantity of crops harvested. For crop prices, we ask about quantities sold and revenue generated, and we compute regional, unit-specific medians of revenue over quantity sold. We also ask about the quantity of each crop harvested in the last 12 months in both the rainy and dry seasons across all the plots owned or managed by members of the household. We omit harvested quantities that have unconvertible units or whose values are extremely unlikely such as outliers and cases where sale value exceeds harvest value. After dropping outlying harvest values, we winsorize values at the 98th percentile. Because we collect harvest quantities at the crop level (not the plot level), if crops are grown on more than one plot, we divide harvest value in proportion to plot size. Finally, we divide harvest value evenly among the owners of a plot to get the beneficiary's share of the value.
Livestock revenue (yearly, USD)	To capture livestock revenue, we ask about the number of animals (bulls, cows, calves, muttons, sheep, goats, camels, donkeys, horses, chickens, and guinea fowls) that the household sold or slaughtered over the last 12 months and we ask about the revenue generated from these sales. We also ask who controls the revenue generated. We winsorize revenues within each animal type and we divide the result by the number of people who control livestock revenue to get the beneficiary's share.
Wage revenue (yearly, USD)	For employment, we ask only beneficiaries and household heads (often the same person) whether they worked at least one hour as apprentices or employees for the state, a company, or anyone outside their household in the last 12 months. We ask them what their income was in that period from such jobs, and we winsorize responses at the job level. For better comparison with other income sources, we scale up this wage income using the regional median monthly profit margin of household businesses.
	**Extended Data Table 4 Off-Farm Businesses**
No. of household businesses	Number of off-farm businesses owned or operated by a household member in the last 12 months.
Business asset value (USD)	We ask about the value of equipment and materials the household currently owns and employs in its off-farm business activities.
Days spent in off-farm business	We ask the beneficiary how many days in the last month they worked in each household business. We sum across businesses within each household.
	**Extended Data Table 5 Agriculture and Livestock Activities**
Area of cultivated crops (ha)	We ask about the size of this land (owned or cultivated) and we omit plots that are under 50 square meters or over 6 hectares when computing household totals.
Agricultural inputs index	Index of agricultural inputs from Supplementary Table SI.7 (seeds, fertilizer, phytosanitary products, and labor) standardized against control group.
Sale value (yearly, USD)	For sale values, we multiply regional, unit-specific median selling prices by quantities sold. As we did for harvest values, we omit sale quantities that have unconvertible units or whose values are extremely unlikely such as outliers and cases where sale value exceeds harvest value. After dropping outlying sales values, we winsorize values at the 98th percentile.

Continued on next page

Supplementary Table SI.3 Variable Definitions and Construction – continued from previous page

Variable	Definition
Days spent in agriculture	We ask the beneficiary how many days in the last rainy season (last 4 months) they worked on household plots. (Max = 120 for 4 months)
Livestock asset value (USD)	We ask each household about the quantity and expected sale value of different types of livestock they currently own. We compute and winsorize unit values at the livestock level.
Days spent raising livestock	We ask the beneficiary how many hours they spent rearing livestock in the last day, how many days in the last month, and how many months in the last year. We compute total days employed per year and divide by 12 to get a monthly average number of days.

For index construction (Extended Data Tables 6-8), see Supplementary Table SI.4

Supplementary Table SI.6 Off-Farm Activities (Household)

Household has a business {0,1}	We ask about all the off-farm income generating activities (businesses) managed by household members in the last 12 months and report whether the household operated any of them.
Main activity off-farm {0,1}	We ask beneficiaries whether the off-farm activity they report to be involved in is their main income-generating activity.
No. of household businesses	See Extended Data Table 3.
Beneficiary's investments (yearly, USD)	We ask beneficiaries how much money they invested in each of these off-farm businesses over the last 12 months. We sum across different funding sources (e.g. NGOs, savings, or friends and family) and we winsorize values at the business level at the 98th percentile.
Business revenue (monthly, USD)	For each business within the household, we ask directly about revenues generated over the last 30 days or in the last month that the business was in operation. We winsorize revenues at the business-level at the 98th percentile. We exclude businesses that are both owned and managed by someone outside the household.
Business profits (monthly, USD)	Construction of profits is similar to revenues as we ask directly about profits from off-farm businesses in the last 30 days.
Beneficiary's healthy business practices index	For beneficiaries who confirm that they are engaged in a productive activity, we ask ten yes/no questions to understand whether they employ good business practices such as tracking creditors, monitoring profitability, seeking feedback from customers, and setting sales targets. Using these ten questions, we construct an index standardized against the control group.

Supplementary Table SI.7 Agriculture (Household)

Cultivated any crop {0,1}	We ask if any member of the household has cultivated any land whether owned or not in the last 12 months.
Area of cultivated crops (ha)	See Extended Data Table 4.
Harvest value (yearly, USD)	Same variable as beneficiary harvest value in Supplementary Table SI.2 but without the scaling for the beneficiary's share.
Lost annual crop {0,1}	We ask about the status of household crops. Households can report that they finished harvesting a crop, that they started but have not yet finished, that they finished, or that they lost an entire harvest. This indicator captures whether a household lost the entire harvest.

Continued on next page

Supplementary Table SI.3 Variable Definitions and Construction – continued from previous page

Variable	Definition
Purchased seeds {0,1}	Indicator for whether a household bought some of the seeds it used on any plot during the rainy season.
Used chemical fertilizer {0,1}	Indicator for whether a household used inorganic or chemical fertilizer on any plot during the rainy season.
Used phytosanitary products {0,1}	Indicator for whether a household used phytosanitary products on any plot during the rainy season.
Used paid labor {0,1}	Indicator for whether a household used paid labor (in cash or in kind) on any plot during the rainy season.
Sold annual crop {0,1}	For crops that have been completely or partially harvested, we ask whether any portion has been sold.
Sale value (yearly, USD)	See Extended Data Table 5.
Commercialization %	Commercialization percent of crops (sale value ÷ harvest value)
**Supplementary Table SI.8 Livestock (Household)**	
Livestock count (TLU)	If a household reports that any of its members owned or raised livestock in the last 12 months, we ask for the number of animals that currently belong to the household itself. We report these numbers using Tropical Livestock Units where conversion factors are as follows [51]:<br>Camels = 1.1; Horses, mares, or donkeys = 0.8; Cows and calves = 0.70; Bulls = 0.5<br>Pigs = 0.20; Sheep, goats, and mutton = 0.10; Guinea Fowl = 0.03; Chicken = 0.01
Livestock asset value (USD)	See Extended Data Table 5.
Change in livestock count (yearly, TLU)	We also ask how many animals any household members owned 12 months ago to compute the change in livestock ownership. We present this change in Tropical Livestock Units as we do for livestock count above.
Livestock purchase value (USD)	If households purchased livestock in the last 12 months, we ask them about the value of their purchases. Like animal sale values, we winsorize purchase values at the animal level at the 98th percentile.
Livestock revenue (yearly, USD)	Same as beneficiary livestock revenue in Extended Data Table 3 but not reduced to beneficiary's share.
**Supplementary Table SI.9 Labor Participation**	
Days spent in off-farm business	See Extended Data Table 4.
Days spent in agriculture	See Extended Data Table 5.
Days spent raising livestock	See Extended Data Table 5.
Days spent in salaried employment	We ask both the beneficiary and household head how many days they spent employed in the last week, how many weeks in the last month, and how many months in the last year. We compute total days employed per year and divide by 12 to get a monthly average number of days. (Max = 30 for each person)
**Supplementary Table SI.10a Financial Engagement**	
Takes part in tontine/AVEC {0,1}	We ask beneficiaries whether they take part in a savings group where savings groups are either tontines where one contributor receives a payout every meeting or VSLAs where payouts are shared among the contributors at the end of every cycle.

Continued on next page

Supplementary Table SI.3 Variable Definitions and Construction – continued from previous page

Variable	Definition
Tontine/AVEC savings (3 months, USD)	We report beneficiaries' savings over the last 3 months by multiplying the size of contributions by their frequency and the number of months in the last 3 months that the beneficiary contributed. We then winsorize these monthly contributions and compute totals regardless of the type of savings group.
Other savings (3 months, USD)	We also ask beneficiaries if they deposited any savings in the last 3 months (1) formally with a bank, savings bank, or other institution, (2) informally in the form of credit with traders or suppliers, agricultural cooperatives, or mobile money or (3) as cash with friends, neighbors, or family members outside the household, or at home in a box or bag, or in the form of jewelry. We winsorize these values at the deposit level at the 98th percentile and we report totals across deposit locations.
Household asset index	We generate a PCA index using the first principal component of the reported current ownership of household assets such as televisions, cellphones, beds, and tables.
	**Supplementary Table SI.11 Assets (Household)**
Agricultural asset value (USD)	We ask about agricultural equipment such as rakes, plows, and pickaxes currently owned by the household. We divide reported values by quantities to get unit values and we winsorize unit values within each asset type.
Business asset value (USD)	See Extended Data Table 4.
	**Supplementary Table SI.12: Potential Mediators of Spill-Over Effects**
Used group labor on farm {0,1}	Indicator for whether a household employed any mutual aid labor groups for any plot during the rainy season.
Used hired labor on farm {0,1}	Indicator for whether a household employed any paid labor for any plot during the rainy season.
Has employees in off-farm activity {0,1}	Indicator for whether a household employed any non-household members in its off-farm income-generating activities in the last 12 months.
Cultivated a plot not owned by household {0,1}	Indicator for whether a household cultivated any plot owned by someone who is not a household member in the rainy season.
Household received private money {0,1}	Indicator for whether a household received money, products, or goods from someone who is not a household member in the last 12 months.
Household sent private money {0,1}	Indicator for whether a household sent cash or in-kind transfers outside the household in the last 12 months.
Community tensions infrequent {1-4}	In the last 6 months, has there been tension between members of your community? 1 yes, a lot - 4 not at all
	**Supplementary Table SI.13: Food Prices**
Log grain prices	We use reported purchase prices to derive a village-level median price for each unit of each food item. We then take a log of village medians.
Log tuber prices	Same construction as log grain prices but for tubers.
Log vegetable prices	Same construction as log grain prices but for vegetables.

Continued on next page

Supplementary Table SI.3 Variable Definitions and Construction – continued from previous page

Variable	Definition
Log meat prices	Same construction as log grain prices but for meats.

Notes: All continuous variables are winsorized at the 98th and 2th percentiles at the most disaggregated level feasible.

Supplementary Table SI.4: Index Definitions and Construction

Variable	Components	Sub-components (if any)	Possible answers
		**Extended Data Table 6 Psychological Well-Being Indices**	
Mental health index	Less depression: Sum of 10 questions from CESD-R-10 [52]	No. of days you felt the details of life bothered you more than usual	0 - 7 days
		No. of days you felt you had trouble concentrating on what you were doing	0 - 7 days
		No. of days you felt sad	0 - 7 days
		No. of days you felt that what you were doing took all your energy	0 - 7 days
		No. of days you felt you were confident in the future (reversed)	0 - 7 days
		No. of days you felt nervous, tense, or worried	0 - 7 days
		No. of days you felt you had trouble sleeping peacefully	0 - 7 days
		No. of days you felt happy (reversed)	0 - 7 days
		No. of days you felt alone	0 - 7 days
		No. of days you felt so tired that you could do nothing	0 - 7 days
	Less disability: Sum of 4 questions from SRQ-20 [53]	No. of days you have had a headache	0 - 7 days
		No. of days your digestion was bad	0 - 7 days
		No. of days you had difficulty fulfilling your family responsibilities	0 - 7 days
		No. of days you had difficulties in your daily work	0 - 7 days
	Life satisfaction today (MacArthur Scale): "Imagine a ladder, with steps numbered from 0 at the bottom to 10 at the top. The top of the ladder represents the best possible life for you and the bottom of the ladder represents the worst possible life for you. On which step of the ladder would you say you personally feel you stand at this time?" [54, 55]		1 lower - 10 higher
	Inner Peace (MacArthur Scale): "Now the staircase represents inner peace. The top of the ladder represents as much inner peace as possible. The bottom of the ladder represents as little inner peace as possible. So if you point up the stairs you have a life with complete or total inner peace, if you point down you have no inner peace. Which step of the stairs are you on these days?"		1 lower - 10 higher

Continued on next page

Supplementary Table SI.4 Index Definitions and Construction – continued from previous page

Variable	Components	Sub-components (if any)	Possible answers
	Self-reported mental health (standardized assessment): "In general, would you say that your mental health is excellent, very good, good, fair or poor?"		1 poor - 5 excellent
Self efficacy index	Index of sum of 7 questions from GSE-10 [56] and 1 from Rosenberg Self-Esteem [57]	GSE 1: You can successfully solve problems if you put in enough effort.	1 not at all - 4 yes, absolutely
		GSE 2: If someone doesn't want you to do something, you can find a way to do whatever you want to do anyway.	1 not at all - 4 yes, absolutely
		GSE 3: It is easy for you to stay on the path you have set out for yourself and achieve your goals in life.	1 not at all - 4 yes, absolutely
		GSE 4: You are confident that you are able to cope well with unexpected events.	1 not at all - 4 yes, absolutely
		GSE 7: You can stay calm when you are faced with difficulties because you have the ability to adapt.	1 not at all - 4 yes, absolutely
		GSE 8: When you have to solve a problem, you can usually find more than one solution.	1 not at all - 4 yes, absolutely
		GSE 9: If you find yourself in a difficult situation, you can usually find a solution.	1 not at all - 4 yes, absolutely
		Rosenberg self-esteem: You are able to do things as well as most people.	1 not at all - 4 yes, absolutely
Future expectations index	Expected social status (MacArthur Scale): "At the top of the stairs are the people with the most resources, the most education, and the most respected activities. At the bottom are the lowest status people who have the least money, the least education, and the least respected or no jobs. The higher you are on this staircase, the closer you are to the people at the top. The lower you are, the closer you get to the people who are at the bottom. In two years, do you think your position will go up, down, or stay the same? What step will you be on in two years?"		1 lower - 10 higher
	Expected life satisfaction two years from now (MacArthur Scale): "Now the staircase represents how satisfied you have been with your life these days. The top of the ladder represents the best possible life for you, one in which the conditions are ideal. The bottom of the ladder represents the worst possible life for you, in which the conditions are not at all the way you want them to be. So if you point up the stairs you are very satisfied with your life, if you point down you are not at all satisfied with your life. In two years, do you think your satisfaction with life will go up, down, or stay the same?"		1 lower - 10 higher

Continued on next page

Supplementary Table SI.4 Index Definitions and Construction – continued from previous page

Variable	Components	Sub-components (if any)	Possible answers
	Expected social status of child at age 30 (MacArthur Scale): "Now let's think about your youngest child (son or daughter) or grandchild (grandson or granddaughter). What will be their position on the status stairs when they turn 30? If they are over 30, indicate their current position."		1 lower - 10 higher
	**Extended Data Table 7 Social Well-Being Indices**		
Financial support index	You can count on your village community to help you in case of financial difficulties.		1 not at all - 4 yes, absolutely
	Sum of number of people whom you could ask for money:	No. of siblings that you can ask for money.	Integer
		No. of family members that you can ask for money.	Integer
		No. of friends that you can ask for money.	Integer
		No. of other people that you can ask for money.	Integer
	How likely is it that you will be able to raise 30,000 XOF over the next month?		1 not at all likely - 4 very likely
Social support index	How many people do you know who have personally succeeded in life?		Integer
	How many people can you go to for advice on income-generating activities?		Integer
	How many people come to you for advice on income-generating activities?		Integer
	How many people can you go to for advice on arguments or disputes?		Integer
	How many people come to you for advice on arguments or disputes?		Integer
	How many people can you trust to sell your products in other markets?		Integer
Social standing index	Index of sum of 4 MacArthur Scale questions	Now, a staircase represents how great a person you are. There are people who are not good people and who do not behave well (point down). But there are also people who have a lot of good qualities and who have good behavior (point the top). Can you point me to the step of the stairs you are on?	1 lower - 10 higher
		Now the staircase represents how respected you are in the community. At the top there are the most respected people, such as the village chief and religious leaders, and at the bottom there are the least respected people, such as the excluded. Can you point me to which step of the staircase you are on these days?	1 lower - 10 higher

Continued on next page

Supplementary Table SI.4 Index Definitions and Construction – continued from previous page

Variable	Components	Sub-components (if any)	Possible answers
		Now the staircase represents how much your opinion is followed in the community. At the top are the people whose opinion is followed the most in the community, and at the bottom are the people whose opinion is least followed. Can you point me to the step of the staircase you are on these days?	1 lower - 10 higher
		At the top of the stairs are the people with the most resources, the most education, and the most respected activities. At the bottom are the lowest status people who have the least money, the least education, and the least respected or unemployed jobs. The higher you are on this staircase, the closer you are to the people at the top. The lower you are, the closer you get to the people who are at the lowest. And you, can you point me to the step of the stairs have you been these days?	1 lower - 10 higher
Social norms index	Descriptive norms index	How many out of 10 women go outside the village when they want?	0 - 10
		Out of 10 women who want to become street vendors, how many will be supported by their parents rather than not being taken seriously?	0 - 10
		To develop their activities, women often need to apply for loans from others. How many in 10 women do you think would receive money from the person they asked?	0 - 10
		How many in 10 women started a new activity in the past 12 months?	0 - 10
	Prescriptive norms index	How many out of 10 men you know think that women should not go freely to other villages or towns?	0 - 10
		How many out of 10 men you know think that women should not have their own income-generating activity?	0 - 10

Continued on next page

Supplementary Table SI.4 Index Definitions and Construction – continued from previous page

Variable	Components	Sub-components (if any)	Possible answers
		How many out of 10 women you know think that women should not go freely to other villages or towns?	0 - 10
		How many out of 10 women you know think that women should not own their own income-generating activity?	0 - 10
Social cohesion and community closeness index	You can count on the help of the women of your village when you have difficulties. Do you agree?		1 not at all - 4 yes, absolutely
	Out of 10 people in the village, how many are honest and trustworthy people?		1 - 10
	Are there people who hate you and wish you failed or to sabotage your success? (reversed)		1 yes, many - 4 none
	In the last 6 months, has there been tension between members of your community? (reversed)		1 yes, a lot - 4 not at all
	How close do you feel to your community? (expressed by surveyor with hand gesture, holding hands far apart versus close together.)		1 independent - 4 integrated
	When making a decision, it is important to take into account the opinions of other members of your community. Do you agree? [58]		1 not at all - 4 yes, absolutely
	(Reversed) You like to be unique and different from other members of your community. Do you agree? [58]		1 not at all - 4 yes, absolutely
	It is your duty to take care of the people in your village, even when you have to sacrifice yourself. Do you agree? [59]		1 not at all - 4 yes, absolutely
	You should always respect the decisions made by your household, even if you don't agree. Do you agree? [59]		1 not at all - 4 yes, absolutely
Collective action index	Number of groups or associations (such as youth groups or tontines) in which respondent has been a member over the last 2 months.		Integer
	Number of positions of responsibility held in any group over the last 12 months.		Integer
	Amount donated to funds for community projects over the last 2 months (winsorized).		Value (USD)
	Number of days volunteered in community activities over the last 2 months.		Integer
	Works with community to achieve common goals.		1 not at all - 4 yes, absolutely
	**Extended Data Table 8 Women's Control Over Earnings and Household Decision-Making**		
Intra-household dynamics index	Partner dynamics index	When you disagree with your spouse, you feel comfortable telling him so.	1 never - 4 most of the time

Continued on next page

Supplementary Table SI.4 Index Definitions and Construction – continued from previous page

Variable	Components	Sub-components (if any)	Possible answers
		Do you think your spouse will always do the things that are best for you?	1 never - 4 most of the time
		How close do you feel to your partner? (expressed by surveyor with hand gesture, holding hands far apart versus close together.)	1 independent - 4 integrated
	Household dynamics index	Your household has NOT prevented you from visiting friends or family in the last 12 months.	0 no - 1 yes
		In the last 6 months, have you had tensions with other members of your household? (reversed)	1 yes, a lot - 4 not at all
		How close do you feel to your household? (expressed by surveyor with hand gesture, holding hands far apart versus close together.)	1 independent - 4 integrated
Violence perceptions index	How many out of 10 women experience tension in their household?		0 - 10
	In this village, is it common for husbands to beat their wives if they burn food.		1 no - 4 very usual
	In this village, is it common for husbands to beat their wives if they neglect the children.		1 no - 4 very usual
Controls earnings index	Does your opinion matter in decisions regarding the money you earn? (The Demographic and Health Surveys Program, USAID)		1 does not matter at all - 3 matters a lot
	Could you make your own decisions without the advice of anyone regarding the money you earn?		1 does not matter at all - 3 matters a lot
	Does your opinion matter in decisions regarding agriculture?		1 does not matter at all - 3 matters a lot
	Does your opinion matter in decisions regarding livestock?		1 does not matter at all - 3 matters a lot
	Does your opinion matter in decisions regarding off-farm activities?		1 does not matter at all - 3 matters a lot
	Your household has NOT prevented you from working outside the home in the last 12 months.		0 no - 1 yes
	You control any revenue from sale of a crop.		0 no - 1 yes
	You own or manage an off-farm business.		0 no - 1 yes
	You own livestock.		0 no - 1 yes
	You control revenue from the sale of livestock.		0 no - 1 yes
	You stayed a night outside the village for a productive (income-generating) purpose in the last 12 months.		0 no - 1 yes
Beneficiary share of total household revenues	Beneficiary's share of total yearly household revenues. See Extended Data Table 2 for construction of total revenues.		Percentage

Continued on next page

Supplementary Table SI.4 Index Definitions and Construction – continued from previous page

Variable	Components	Sub-components (if any)	Possible answers
Controls household resources index	Does your opinion matter in decisions regarding the money your partner earns?		1 does not matter at all - 3 matters a lot
	Does your opinion matter in decisions regarding current household expenses?		1 does not matter at all - 3 matters a lot
	Does your opinion matter in decisions regarding major household purchases?		1 does not matter at all - 3 matters a lot
	Does your opinion matter in decisions regarding family planning?		1 does not matter at all - 3 matters a lot
	Does your opinion matter in decisions regarding your own healthcare?		1 does not matter at all - 3 matters a lot
	Does your opinion matter in decisions regarding your children's education?		1 does not matter at all - 3 matters a lot
	Could you make your own decisions without the advice of anyone regarding current household expenses?		1 does not matter at all - 3 matters a lot
	Could you make your own decisions without the advice of anyone regarding household purchases?		1 does not matter at all - 3 matters a lot
	Could you make your own decisions without the advice of anyone regarding family planning?		1 does not matter at all - 3 matters a lot
	Could you make your own decisions without the advice of anyone regarding your own healthcare?		1 does not matter at all - 3 matters a lot

Notes: The variable listed in the first column is a z-score index of the components listed in the second column. Some components are made up of sub-components, which are shown to the right of the components. All indices are standardized against the control group.

Supplementary Table SI.5: Multiple Hypothesis Test Corrections

MHT family	6 months post-intervention									18 months post-intervention								
	Capital Arm			Psychosocial Arm			Full Arm			Capital Arm			Psychosocial Arm			Full Arm		
Variable	Actual	FDR[†]	FWER[‡]	Actual	FDR	FWER	Actual	FDR	FWER	Actual	FDR	FWER	Actual	FDR	FWER	Actual	FDR	FWER
**Extended Data Table 1 Consumption and Food Security**																		
Gross consumption (daily, USD/adult eq.)	**0.003**	**0.003**	**0.008**	*0.094*	*0.094*	0.130	**0**	**0**	**0**	**0.008**	**0.008**	**0.020**	**0**	**0.001**	**0.002**	**0**	**0**	**0**
Food security	**0**	**0**	**0**	*0.058*	*0.087*	0.153	**0**	**0**	**0**	**0**	**0**	**0**	**0**	**0.001**	**0.004**	**0**	**0**	**0**
Dietary diversity	**0**	**0**	**0**	**0.002**	**0.005**	**0.016**	**0**	**0**	**0**	**0**	**0.001**	**0.002**	**0.009**	**0.009**	**0.019**	**0**	**0**	**0**
**Extended Data Table 2 Household Revenues**																		
Total revenue (yearly, USD, HH)	**0**	**0**	**0**	**0.001**	**0.001**	**0.007**	**0**	**0**	**0**	**0**	**0**	**0.001**	**0**	**0**	**0**	**0**	**0**	**0**
Business revenue (yearly, USD, HH)	**0**	**0**	**0**	**0**	**0**	**0**	**0**	**0**	**0**	**0**	**0.001**	**0.008**	**0**	**0**	**0**	**0**	**0**	**0**
Harvest value (yearly, USD, HH)	0.563	0.836	0.935	0.636	0.636	0.673	0.742	0.742	0.772	0.145	0.181	0.318	**0**	**0**	**0.002**	**0**	**0**	**0.002**
Livestock revenue (yearly, USD, HH)	0.668	0.836	0.914	0.191	0.319	0.527	**0.011**	**0.018**	*0.063*	**0**	**0**	**0**	**0.036**	**0.044**	0.105	**0**	**0**	**0.001**
Wage revenue (yearly, USD, HH)	0.921	0.921	0.929	0.484	0.605	0.780	0.659	0.742	0.905	0.730	0.730	0.751	0.419	0.419	0.460	0.974	0.974	0.978
**Extended Data Table 3 Beneficiary Revenues**																		
Total revenue (yearly, USD, benef.)	**0**	**0**	**0**	**0**	**0**	**0**	**0**	**0**	**0**	**0**	**0**	**0**	**0**	**0**	**0**	**0**	**0**	**0**
Business revenue (yearly, USD, benef.)	**0**	**0**	**0**	**0**	**0**	**0**	**0**	**0**	**0**	**0**	**0**	**0**	**0**	**0**	**0**	**0**	**0**	**0**
Harvest value (yearly, USD, benef.)	0.264	0.264	0.375	0.345	0.345	0.455	0.375	0.375	0.477	0.198	0.198	0.213	**0.004**	**0.006**	**0.045**	**0.002**	**0.003**	**0.007**
Livestock revenue (yearly, USD, benef.)	0.244	0.264	0.494	**0.029**	**0.049**	0.162	0.162	0.202	0.377	**0.007**	**0.011**	**0.028**	0.152	0.190	0.354	**0.005**	**0.006**	**0.020**
Wage revenue (yearly, USD, benef.)	**0.025**	**0.041**	0.289	0.226	0.283	0.463	**0.030**	**0.049**	0.141	0.119	0.149	0.302	0.701	0.701	0.742	**0.036**	**0.036**	*0.070*
**Extended Data Table 6 Psychological Well-Being Indices**																		
Mental health index	**0.003**	**0.004**	**0.017**	**0.016**	**0.016**	**0.025**	**0**	**0**	**0**	**0**	**0**	**0**	**0**	**0**	**0**	**0**	**0**	**0**
Self efficacy index	**0.005**	**0.005**	**0.009**	**0**	**0**	**0**	**0**	**0**	**0**	**0.019**	**0.019**	**0.033**	**0**	**0**	**0**	**0**	**0**	**0**
Future expectations index	**0.002**	**0.004**	**0.014**	**0**	**0**	**0**	**0**	**0**	**0**	**0.011**	**0.017**	**0.040**	**0.002**	**0.002**	**0.006**	**0**	**0**	**0**
**Extended Data Table 7 Social Well-Being Indices**																		
Financial support index	**0**	**0**	**0**	**0**	**0**	**0**	**0**	**0**	**0**	**0**	**0**	**0**	**0**	**0**	**0**	**0**	**0**	**0**
Social support index	**0**	**0**	**0.001**	**0**	**0**	**0**	**0**	**0**	**0**	**0.004**	**0.007**	**0.023**	**0**	**0**	**0**	**0**	**0**	**0**
Social standing index	**0.001**	**0.001**	**0.002**	**0**	**0**	**0**	**0**	**0**	**0**	*0.082*	*0.098*	0.205	**0.005**	**0.006**	**0.021**	**0**	**0**	**0**
Social norms index	**0.001**	**0.001**	**0.004**	**0**	**0**	**0**	**0**	**0**	**0**	0.109	0.109	0.143	**0.014**	**0.014**	**0.024**	**0.001**	**0.001**	**0.006**
Social cohesion and community closeness index	0.230	0.230	0.265	**0.012**	**0.012**	**0.016**	**0.013**	**0.013**	**0.021**	**0.031**	**0.046**	0.162	**0**	**0**	**0**	**0.035**	**0.035**	*0.056*
Collective action index	**0**	**0**	**0**	**0**	**0**	**0**	**0**	**0**	**0**	**0**	**0**	**0**	**0**	**0**	**0**	**0**	**0**	**0**
**Extended Data Table 8 Women's Control Over Earnings and Household Decision-Making**																		
Intra-household dynamics index	0.114	0.191	0.384	*0.094*	0.157	0.328	0.108	0.177	0.376	0.634	0.776	0.884	0.323	0.323	0.367	0.787	0.787	0.791
Violence perceptions index	0.231	0.289	0.440	0.317	0.317	0.353	0.281	0.281	0.317	0.593	0.776	0.941	*0.064*	0.107	0.228	**0.008**	**0.014**	**0.044**
Controls earnings index	**0**	**0**	**0**	**0**	**0**	**0**	**0**	**0**	**0**	**0**	**0**	**0**	**0**	**0.002**	**0**	**0**	**0**	**0**
Benef. share of total HH revenues	**0**	**0**	**0**	**0**	**0**	**0**	**0**	**0**	**0**	**0**	**0**	**0**	**0.035**	*0.088*	0.196	**0**	**0**	**0**
Controls HH resources index	0.496	0.496	0.532	0.167	0.208	0.352	0.142	0.177	0.314	0.776	0.776	0.784	0.234	0.292	0.454	0.419	0.524	0.707
**Supplementary Table SI.6 Off-Farm Activities (Household)**																		
Household has a business {0,1}	**0**	**0**	**0**	**0**	**0**	**0**	**0**	**0**	**0**	**0**	**0**	**0**	**0**	**0**	**0.002**	**0**	**0**	**0**

Continued on next page

Supplementary Table SI.5: Multiple Hypothesis Test Corrections – continued from previous page

MHT family	6 months post-intervention									18 months post-intervention								
	Capital Arm			Psychosocial Arm			Full Arm			Capital Arm			Psychosocial Arm			Full Arm		
Variable	Actual	FDR†	FWER‡	Actual	FDR	FWER	Actual	FDR	FWER	Actual	FDR	FWER	Actual	FDR	FWER	Actual	FDR	FWER
Beneficiary's main activity is off-farm {0,1}	**0**	**0**	**0**	**0**	**0**	**0**	**0**	**0**	**0**	**0**	**0**	**0.004**	**0.001**	**0.001**	**0.006**	**0**	**0**	**0**
No. of household businesses	**0**	**0**	**0**	**0**	**0**	**0**	**0**	**0**	**0**	**0**	**0**	**0**	**0**	**0**	**0**	**0**	**0**	**0**
Beneficiary's investments (yearly, USD)	**0**	**0**	**0**	**0**	**0**	**0**	**0**	**0**	**0**	**0**	**0**	**0**	**0.001**	**0.001**	**0.005**	**0**	**0**	**0**
Business revenue (monthly, USD)	**0**	**0**	**0**	**0**	**0**	**0**	**0**	**0**	**0**	**0.001**	**0.001**	**0.007**	**0.002**	**0.002**	**0.007**	**0**	**0**	**0**
Business profits (monthly, USD)	**0**	**0**	**0**	**0**	**0**	**0**	**0**	**0**	**0**	**0.002**	**0.002**	**0.008**	**0.003**	**0.003**	**0.004**	**0**	**0**	**0**
Beneficiary's healthy business practices index	**0**	**0**	**0**	**0**	**0**	**0**	**0**	**0**	**0**	**0**	**0**	**0**	**0**	**0.001**	**0.005**	**0**	**0**	**0**
**Supplementary Table SI.7 Agriculture (Household)**																		
Cultivated any crop {0,1}	0.843	0.843	0.855	0.858	0.858	0.850	0.845	0.845	0.877	0.293	0.503	0.918	0.412	0.504	0.746	0.819	0.819	0.846
Area of cultivated crops (ha)	0.186	0.319	0.752	**0.015**	**0.046**	0.206	**0.018**	**0.036**	0.195	0.746	0.884	0.972	0.270	0.460	0.918	0.410	0.547	0.882
Harvest value (yearly, USD)	0.563	0.676	0.938	0.636	0.740	0.968	0.742	0.810	0.950	0.145	0.290	0.670	**0**	**0.001**	**0.008**	**0**	**0.001**	**0.009**
Lost annual crop {0,1}	0.705	0.769	0.928	0.629	0.740	0.987	0.496	0.595	0.888	0.433	0.578	0.888	0.292	0.460	0.881	0.125	0.213	0.573
Purchased seeds {0,1}	0.221	0.332	0.763	0.523	0.740	0.985	0.261	0.348	0.764	*0.055*	0.220	0.506	*0.055*	0.221	0.489	0.155	0.232	0.612
Used chemical fertilizer {0,1}	**0.014**	*0.055*	0.203	0.287	0.491	0.883	**0.003**	**0.013**	*0.063*	**0.006**	*0.068*	0.105	0.462	0.504	0.827	**0.001**	**0.002**	**0.023**
Used pytosanitary products {0,1}	**0.026**	*0.062*	0.237	0.679	0.740	0.911	**0.008**	**0.019**	0.118	0.118	0.284	0.697	0.232	0.460	0.920	**0**	**0.001**	**0**
Used paid labor {0,1}	**0.003**	**0.019**	*0.074*	*0.064*	0.153	0.445	**0**	**0**	**0**	0.116	0.284	0.679	0.293	0.460	0.909	**0**	**0**	**0**
Agricultural inputs index	**0**	**0.002**	**0.008**	0.114	0.228	0.611	**0**	**0**	**0**	*0.054*	0.220	0.479	0.548	0.548	0.573	**0**	**0**	**0**
Sold annual crop {0,1}	0.419	0.559	0.903	**0.003**	**0.016**	*0.071*	*0.052*	*0.089*	0.347	0.851	0.884	0.966	0.307	0.460	0.836	0.547	0.657	0.898
Sale value (yearly, USD)	**0.019**	*0.057*	0.193	**0.004**	**0.016**	*0.075*	**0.006**	**0.018**	*0.061*	0.428	0.578	0.924	**0.012**	*0.073*	0.225	*0.073*	0.146	0.451
Commercialization %	**0.037**	*0.073*	0.290	**0**	**0.005**	**0.012**	*0.087*	0.130	0.409	0.884	0.884	0.893	0.445	0.504	0.916	0.725	0.791	0.937
**Supplementary Table SI.8 Livestock (Household)**																		
Livestock count (TLU)	**0**	**0**	**0**	**0**	**0.001**	**0**	**0**	**0**	**0**	**0**	**0**	**0**	**0.001**	**0.004**	**0.011**	**0**	**0**	**0**
Livestock asset value (USD)	**0.001**	**0.001**	**0**	0.145	0.319	0.492	**0**	**0**	**0**	**0.001**	**0.002**	**0.018**	*0.096*	0.161	0.326	**0**	**0**	**0**
Change in livestock count (yearly, TLU)	**0**	**0**	**0.004**	0.403	0.504	0.722	**0.018**	**0.018**	*0.061*	0.666	0.666	0.689	0.396	0.495	0.666	0.277	0.277	0.304
Livestock purchase value (USD)	**0**	**0**	**0**	0.554	0.554	0.580	**0**	**0**	**0**	0.238	0.298	0.501	0.860	0.860	0.884	**0.017**	**0.021**	**0.046**
Livestock revenue (yearly, USD)	0.668	0.668	0.709	0.191	0.319	0.521	**0.011**	**0.014**	**0.042**	**0**	**0**	**0**	**0.036**	*0.089*	0.186	**0**	**0**	**0.001**
**Supplementary Table SI.9a Labor Participation (Household)**																		
Days spent in off-farm business (HH)	**0**	**0**	**0**	**0**	**0**	**0**	**0**	**0**	**0**	**0**	**0**	**0**	**0**	**0**	**0**	**0**	**0**	**0**
Days spent in agriculture (HH)	0.917	0.917	0.931	**0.013**	**0.026**	*0.063*	**0.029**	**0.038**	*0.086*	0.975	0.975	0.978	0.852	0.852	0.858	0.395	0.526	0.648
Days spent raising livestock (HH)	**0.002**	**0.003**	**0.014**	*0.089*	0.118	0.221	**0**	**0**	**0**	**0**	**0**	**0**	0.123	0.245	0.375	**0**	**0**	**0**
Days spent in salaried employment (HH)	0.156	0.208	0.354	0.190	0.190	0.227	**0.041**	**0.041**	*0.066*	0.923	0.975	0.994	0.274	0.365	0.543	0.583	0.583	0.626
**Supplementary Table SI.9b Labor Participation (Beneficiary)**																		
Days spent in off-farm business (Benef.)	**0**	**0**	**0**	**0**	**0**	**0**	**0**	**0**	**0**	**0**	**0**	**0**	**0**	**0**	**0**	**0**	**0**	**0**
Days spent in agriculture (Benef.)	0.398	0.398	0.442	0.479	0.479	0.536	0.833	0.833	0.847	0.377	0.503	0.645	0.593	0.773	0.850	0.682	0.682	0.698
Days spent raising livestock (Benef.)	**0**	**0**	**0**	**0.006**	**0.012**	**0.034**	**0**	**0**	**0**	**0**	**0**	**0**	**0.010**	**0.020**	*0.068*	**0**	**0**	**0**
Days spent in salaried employment (Benef.)	0.171	0.228	0.367	*0.051*	*0.068*	0.140	**0.025**	**0.033**	0.112	0.699	0.699	0.713	0.773	0.773	0.779	0.285	0.381	0.540
**Supplementary Table SI.10a Financial Engagement**																		
Takes part in tontine/AVEC {0,1}	**0**	**0**	**0.010**	**0.001**	**0.003**	*0.059*	**0.001**	**0.003**	**0.023**	**0**	**0**	**0**	**0**	**0**	**0**	**0**	**0**	**0**

Continued on next page

Supplementary Table SI.5: Multiple Hypothesis Test Corrections – continued from previous page

	6 months post-intervention									18 months post-intervention								
**MHT family**	Capital Arm			Psychosocial Arm			Full Arm			Capital Arm			Psychosocial Arm			Full Arm		
Variable	Actual	FDR†	FWER‡	Actual	FDR	FWER	Actual	FDR	FWER	Actual	FDR	FWER	Actual	FDR	FWER	Actual	FDR	FWER
Tontine/AVEC savings (3 months, USD)	0.525	0.525	0.557	0.485	0.485	0.515	0.297	0.297	0.328	**0**	**0**	**0**	**0**	**0**	**0**	**0**	**0**	**0**
Other savings (3 months, USD)	*0.060*	0.120	0.232	**0.036**	**0.047**	*0.095*	**0**	**0**	**0**	**0.008**	**0.010**	**0.029**	0.457	0.457	0.491	**0.001**	**0.001**	**0.003**
Household asset index	0.280	0.373	0.523	**0.027**	**0.047**	0.135	**0.002**	**0.003**	**0.019**	0.478	0.478	0.504	**0.020**	**0.027**	*0.058*	**0.007**	**0.007**	**0.018**
**Supplementary Table SI.11 Assets (Household)**																		
Agricultural asset value (USD)	0.937	0.937	0.942	0.426	0.426	0.449	0.981	0.981	0.981	0.148	0.148	0.192	0.825	0.825	0.843	*0.072*	*0.072*	*0.092*
Business asset value (USD)	0.178	0.355	0.364	0.246	0.426	0.480	**0**	**0.001**	**0.006**	**0.023**	**0.046**	*0.069*	*0.072*	0.144	0.189	**0.009**	**0.018**	**0.033**

Notes: For variables in Extended Data Tables 4 and 5, see Supplementary Tables SI.6-SI.8. At each survey phase and within each treatment arm, we correct p-values for multiple hypothesis testing within each family of variables. Columns labeled *actual* show the p-values used in the main tables. Italics: $p < 0.1$. Bold: $p < 0.05$. Bold and underlined: $p < 0.01$.

†: Columns labeled FDR show False Discovery Rate-adjusted q-values following the step-up approach of Benjamini & Hochberg [60] which assumes that the p-values within a family are positively correlated.

‡: Columns labeled FWER show Family-Wise Error Rate-corrected p-values following the procedure outlined by Barsbai *et al.* [61] that captures the existing correlation between p-values by exploiting treatment randomization and runing a bootstrap resampling procedure.

In the pre-analysis plan, we indicate we would apply multiple hypothesis corrections on the set of tests that include all individual arm treatment effects on each outcome within an outcome family. Here we instead only correct for multiple hypotheses *within* each treatment arm across the outcomes in each family. We now believe this is sufficient because treatment arms were pre-defined.

Changes to the pre-specified outcomes families were only made only for ease of display, interpretability, and comparisons. For the avoidance of doubt, the multi-hypothesis tests are shown for the originally defined outcome families.

Supplementary Table SI.6: Off-Farm Activities (Household)

		Capital (Full w/o Psych.)	Psych. (Full w/o Capital)	Full	Ctrl mean/ Ctrl SD/ N	Full - Psych. (Cash grant gross ME)	Full - Capital (Psych. comp. gross ME)	Capital - Psych.	18m - 6m for Capital	18m - 6m for Psych.	18m - 6m for Full
		coef/se/p				coef/se/p			coef/se/p		
Household has a	6m	0.10	0.11	0.11	0.68	0.00	0.02	-0.02	-0.01	-0.03	-0.01
business {0,1}		(0.02)	(0.02)	(0.02)	0.47	(0.02)	(0.02)	(0.02)	(0.02)	(0.02)	(0.02)
		(0.000)	(0.000)	(0.000)	4,476	(0.878)	(0.334)	(0.431)	(0.789)	(0.162)	(0.578)
	18m	0.09	0.08	0.10	0.71	0.02	0.01	0.01			
		(0.02)	(0.02)	(0.02)	0.45	(0.02)	(0.02)	(0.02)			
		(0.000)	(0.000)	(0.000)	4,303	(0.254)	(0.559)	(0.622)			
Beneficiary's	6m	0.12	0.12	0.16	0.26	0.04	0.05	-0.00	-0.03	-0.05	-0.04
main activity is		(0.02)	(0.02)	(0.02)	0.44	(0.02)	(0.02)	(0.02)	(0.02)	(0.02)	(0.02)
off-farm {0,1}		(0.000)	(0.000)	(0.000)	4,476	(0.040)	(0.021)	(0.875)	(0.142)	(0.023)	(0.059)
	18m	0.08	0.07	0.12	0.26	0.05	0.04	0.02			
		(0.02)	(0.02)	(0.02)	0.44	(0.02)	(0.02)	(0.02)			
		(0.000)	(0.001)	(0.000)	4,303	(0.007)	(0.071)	(0.371)			
No. of	6m	0.28	0.28	0.41	1.08	0.13	0.13	-0.01	-0.04	-0.03	-0.06
household		(0.05)	(0.05)	(0.05)	1.04	(0.05)	(0.05)	(0.05)	(0.06)	(0.05)	(0.06)
businesses		(0.000)	(0.000)	(0.000)	4,476	(0.014)	(0.013)	(0.905)	(0.513)	(0.523)	(0.252)
	18m	0.24	0.25	0.35	1.10	0.10	0.11	-0.01			
		(0.05)	(0.05)	(0.05)	0.99	(0.05)	(0.05)	(0.05)			
		(0.000)	(0.000)	(0.000)	4,303	(0.065)	(0.037)	(0.884)			
Beneficiary's	6m	27.99	19.22	38.72	23.94	19.50	10.74	8.76	-14.18	-11.04	-23.53
investments		(4.17)	(4.11)	(4.82)	68.25	(5.08)	(5.07)	(4.55)	(3.75)	(3.69)	(4.13)
(yearly, USD)		(0.000)	(0.000)	(0.000)	4,476	(0.000)	(0.035)	(0.055)	(0.000)	(0.003)	(0.000)
	18m	13.80	8.18	15.19	19.84	7.01	1.39	5.62			
		(2.80)	(2.42)	(2.98)	45.59	(2.90)	(3.22)	(2.81)			
		(0.000)	(0.001)	(0.000)	4,252	(0.016)	(0.666)	(0.046)			
Business revenue	6m	49.90	36.40	61.66	73.44	25.26	11.76	13.50	-21.64	-11.75	-13.37
(monthly, USD)		(8.31)	(7.38)	(9.47)	153.94	(9.58)	(10.16)	(8.69)	(8.26)	(8.96)	(9.28)
		(0.000)	(0.000)	(0.000)	4,476	(0.009)	(0.248)	(0.121)	(0.009)	(0.189)	(0.150)
	18m	28.26	24.64	48.29	73.31	23.65	20.03	3.62			
		(8.16)	(7.76)	(8.64)	178.03	(8.65)	(8.88)	(8.32)			
		(0.001)	(0.002)	(0.000)	4,303	(0.007)	(0.025)	(0.664)			
Business profits	6m	14.10	11.35	20.34	29.15	8.99	6.24	2.76	-5.21	-2.59	-4.36
(monthly, USD)		(2.75)	(2.63)	(3.44)	57.70	(3.53)	(3.60)	(3.03)	(3.34)	(3.43)	(3.60)
		(0.000)	(0.000)	(0.000)	4,476	(0.011)	(0.084)	(0.363)	(0.119)	(0.451)	(0.226)
	18m	8.89	8.76	15.98	27.94	7.22	7.09	0.13			
		(2.89)	(2.88)	(3.18)	60.06	(3.23)	(3.16)	(3.00)			
		(0.002)	(0.003)	(0.000)	4,303	(0.026)	(0.026)	(0.966)			
Beneficiary's	6m	0.44	0.31	0.46	0.00	0.16	0.02	0.13	-0.22	-0.15	-0.22
healthy business		(0.06)	(0.05)	(0.06)	1.00	(0.06)	(0.06)	(0.06)	(0.06)	(0.06)	(0.06)
practices index		(0.000)	(0.000)	(0.000)	4,283	(0.006)	(0.706)	(0.028)	(0.000)	(0.016)	(0.000)
	18m	0.22	0.16	0.25	0.00	0.09	0.03	0.06			
		(0.04)	(0.05)	(0.04)	1.00	(0.05)	(0.04)	(0.05)			
		(0.000)	(0.000)	(0.000)	4,134	(0.063)	(0.538)	(0.192)			

Notes: Results presented are OLS estimates that include controls for randomization strata and, where possible, baseline outcomes. We assign baseline strata means to households surveyed at midline or endline but not at baseline and we control for such missing values with an indicator. See Table SI.3 for details on variable construction. Robust standard errors, clustered at the village level, and two-tailed p-values are shown in parentheses. All monetary amounts are PPP-adjusted USD terms, set at 2016 prices and deflated using Niger CPI published by the World Bank. In 2016, 1 USD = 242.553 XOF PPP. All continuous variables are winsorized at the 98th and 2th percentiles at the most disaggregated level feasible.

Supplementary Table SI.7: Agriculture (Household)

		Capital (Full w/o Psych.)	Psych. (Full w/o Capital)	Full	Ctrl mean/ Ctrl SD/ N	Full - Psych. (Cash grant gross ME)	Full - Capital (Psych. comp. gross ME)	Capital - Psych.	18m - 6m for Capital	18m - 6m for Psych.	18m - 6m for Full
		coef/se/p				coef/se/p			coef/se/p		
Cultivated any crop {0,1}	6m	0.00	0.00	-0.00	0.96	-0.00	-0.00	0.00	-0.01	-0.01	0.00
		(0.01)	(0.01)	(0.01)	0.19	(0.01)	(0.01)	(0.01)	(0.01)	(0.01)	(0.01)
		(0.843)	(0.858)	(0.845)	4,476	(0.717)	(0.697)	(0.974)	(0.256)	(0.386)	(0.988)
	18m	-0.01	-0.00	-0.00	0.98	0.00	0.01	-0.00			
		(0.01)	(0.01)	(0.01)	0.14	(0.01)	(0.01)	(0.01)			
		(0.293)	(0.412)	(0.819)	4,303	(0.592)	(0.389)	(0.796)			
Area of cultivated crops (ha)	6m	0.17	0.30	0.28	3.56	-0.02	0.11	-0.12	-0.22	-0.15	-0.16
		(0.13)	(0.12)	(0.12)	2.86	(0.13)	(0.13)	(0.14)	(0.14)	(0.15)	(0.15)
		(0.186)	(0.015)	(0.018)	4,476	(0.895)	(0.416)	(0.382)	(0.126)	(0.335)	(0.300)
	18m	-0.04	0.15	0.12	3.65	-0.03	0.17	-0.19			
		(0.13)	(0.14)	(0.15)	2.75	(0.14)	(0.14)	(0.13)			
		(0.746)	(0.270)	(0.410)	4,303	(0.848)	(0.231)	(0.138)			
Harvest value (yearly, USD)	6m	-29.94	-22.01	17.45	538.74	39.46	47.40	-7.93	61.51	113.11	62.57
		(51.71)	(46.52)	(53.05)	1,617.94	(33.80)	(34.90)	(28.80)	(55.80)	(50.40)	(56.57)
		(0.563)	(0.636)	(0.742)	4,476	(0.244)	(0.175)	(0.783)	(0.270)	(0.025)	(0.269)
	18m	31.57	91.10	80.02	320.15	-11.08	48.45	-59.53			
		(21.61)	(23.00)	(21.59)	353.22	(24.90)	(22.28)	(24.49)			
		(0.145)	(0.000)	(0.000)	4,303	(0.657)	(0.030)	(0.016)			
Lost annual crop {0,1}	6m	0.00	0.01	-0.01	0.10	-0.02	-0.01	-0.00	0.01	-0.02	-0.02
		(0.01)	(0.01)	(0.01)	0.30	(0.01)	(0.01)	(0.01)	(0.02)	(0.02)	(0.02)
		(0.705)	(0.629)	(0.496)	4,476	(0.271)	(0.274)	(0.879)	(0.672)	(0.272)	(0.405)
	18m	0.01	-0.02	-0.02	0.18	-0.01	-0.04	0.03			
		(0.02)	(0.02)	(0.02)	0.39	(0.02)	(0.02)	(0.02)			
		(0.433)	(0.292)	(0.125)	4,303	(0.624)	(0.024)	(0.088)			
Purchased seeds {0,1}	6m	0.02	0.01	0.02	0.54	0.01	-0.00	0.01	-0.06	-0.05	-0.05
		(0.02)	(0.02)	(0.02)	0.50	(0.02)	(0.02)	(0.02)	(0.02)	(0.03)	(0.03)
		(0.221)	(0.523)	(0.261)	4,476	(0.620)	(0.992)	(0.584)	(0.009)	(0.046)	(0.037)
	18m	-0.04	-0.04	-0.03	0.48	0.01	0.01	-0.00			
		(0.02)	(0.02)	(0.02)	0.50	(0.02)	(0.02)	(0.02)			
		(0.055)	(0.055)	(0.155)	4,303	(0.680)	(0.647)	(0.955)			
Used chemical fertilizer {0,1}	6m	0.05	0.02	0.05	0.14	0.03	0.00	0.03	0.00	-0.00	0.01
		(0.02)	(0.02)	(0.02)	0.35	(0.02)	(0.02)	(0.02)	(0.02)	(0.02)	(0.02)
		(0.014)	(0.287)	(0.003)	4,476	(0.066)	(0.986)	(0.139)	(0.963)	(0.841)	(0.620)
	18m	0.05	0.01	0.06	0.15	0.05	0.01	0.04			
		(0.02)	(0.02)	(0.02)	0.35	(0.02)	(0.02)	(0.02)			
		(0.006)	(0.462)	(0.001)	4,303	(0.014)	(0.590)	(0.046)			
Used pytosanitary products {0,1}	6m	0.03	0.01	0.03	0.09	0.03	0.00	0.02	-0.01	0.01	0.01
		(0.01)	(0.01)	(0.01)	0.28	(0.01)	(0.01)	(0.01)	(0.02)	(0.02)	(0.02)
		(0.026)	(0.679)	(0.008)	4,476	(0.033)	(0.711)	(0.090)	(0.557)	(0.557)	(0.391)
	18m	0.02	0.01	0.05	0.07	0.03	0.03	0.00			
		(0.01)	(0.01)	(0.01)	0.25	(0.01)	(0.01)	(0.01)			
		(0.118)	(0.232)	(0.000)	4,303	(0.010)	(0.016)	(0.757)			
Used paid labor {0,1}	6m	0.04	0.03	0.08	0.14	0.05	0.03	0.02	-0.02	-0.01	0.01
		(0.01)	(0.02)	(0.02)	0.35	(0.02)	(0.02)	(0.02)	(0.02)	(0.02)	(0.02)
		(0.003)	(0.064)	(0.000)	4,476	(0.007)	(0.060)	(0.365)	(0.327)	(0.514)	(0.565)
	18m	0.03	0.02	0.09	0.13	0.07	0.06	0.01			
		(0.02)	(0.02)	(0.02)	0.34	(0.02)	(0.02)	(0.02)			
		(0.116)	(0.293)	(0.000)	4,303	(0.000)	(0.001)	(0.609)			
Agricultural inputs index	6m	0.20	0.08	0.25	0.00	0.17	0.05	0.11	-0.10	-0.05	-0.00
		(0.05)	(0.05)	(0.05)	1.00	(0.05)	(0.05)	(0.06)	(0.06)	(0.06)	(0.06)
		(0.000)	(0.114)	(0.000)	4,476	(0.003)	(0.321)	(0.051)	(0.083)	(0.393)	(0.964)
	18m	0.10	0.03	0.25	0.00	0.22	0.15	0.07			
		(0.05)	(0.05)	(0.05)	1.00	(0.05)	(0.05)	(0.05)			
		(0.054)	(0.548)	(0.000)	4,303	(0.000)	(0.002)	(0.175)			
Sold annual crop {0,1}	6m	0.02	0.06	0.04	0.45	-0.02	0.02	-0.05	-0.02	-0.04	-0.03
		(0.02)	(0.02)	(0.02)	0.50	(0.02)	(0.02)	(0.02)	(0.03)	(0.02)	(0.03)
		(0.419)	(0.003)	(0.052)	4,476	(0.272)	(0.298)	(0.045)	(0.376)	(0.076)	(0.274)
	18m	-0.00	0.02	0.01	0.35	-0.01	0.02	-0.03			
		(0.02)	(0.02)	(0.02)	0.48	(0.02)	(0.02)	(0.02)			
		(0.851)	(0.307)	(0.547)	4,303	(0.680)	(0.429)	(0.243)			
Sale value (yearly, USD)	6m	17.61	18.32	16.67	61.65	-1.66	-0.94	-0.72	-14.82	-8.45	-9.28
		(7.47)	(6.32)	(6.01)	125.47	(6.61)	(7.53)	(8.14)	(7.17)	(6.46)	(6.52)
		(0.019)	(0.004)	(0.006)	4,476	(0.802)	(0.901)	(0.930)	(0.039)	(0.191)	(0.154)
	18m	2.79	9.87	7.38	30.07	-2.49	4.59	-7.08			
		(3.52)	(3.91)	(4.11)	69.56	(4.29)	(4.00)	(4.01)			
		(0.428)	(0.012)	(0.073)	4,303	(0.563)	(0.252)	(0.078)			

Continued on next page

Supplementary Table SI.7: Agriculture (Household) – continued from previous page

		Capital (Full w/o Psych.)	Psych. (Full w/o Capital)	Full	Ctrl mean/ Ctrl SD/ N	Full - Psych. (Cash grant gross ME)	Full - Capital (Psych. comp. gross ME)	Capital - Psych.	18m - 6m for Capital	18m - 6m for Psych.	18m - 6m for Full
		coef/se/p				coef/se/p			coef/se/p		
Commercial-ization %	6m	0.02	0.03	0.02	0.12	-0.02	-0.00	-0.01	-0.02	-0.03	-0.02
		(0.01)	(0.01)	(0.01)	0.18	(0.01)	(0.01)	(0.01)	(0.01)	(0.01)	(0.01)
		(0.037)	(0.000)	(0.087)	3,701	(0.114)	(0.767)	(0.204)	(0.114)	(0.017)	(0.078)
	18m	0.00	0.01	-0.00	0.09	-0.01	-0.00	-0.01			
		(0.01)	(0.01)	(0.01)	0.17	(0.01)	(0.01)	(0.01)			
		(0.884)	(0.445)	(0.725)	3,530	(0.240)	(0.620)	(0.562)			

Notes: Results presented are OLS estimates that include controls for randomization strata and, where possible, baseline outcomes. We assign baseline strata means to households surveyed at midline or endline but not at baseline and we control for such missing values with an indicator. See Table SI.3 for details on variable construction. Robust standard errors, clustered at the village level, and two-tailed p-values are shown in parentheses. All monetary amounts are PPP-adjusted USD terms, set at 2016 prices and deflated using Niger CPI published by the World Bank. In 2016, 1 USD = 242.553 XOF PPP. All continuous variables are winsorized at the 98th and 2th percentiles at the most disaggregated level feasible.

Supplementary Table SI.8: Livestock (Household)

		Capital (Full w/o Psych.)	Psych. (Full w/o Capital)	Full	Ctrl mean/ Ctrl SD/ N	Full - Psych. (Cash grant gross ME)	Full - Capital (Psych. comp. gross ME)	Capital - Psych.	18m - 6m for Capital	18m - 6m for Psych.	18m - 6m for Full
		coef/se/p				coef/se/p			coef/se/p		
Livestock count (TLU)	6m	0.75	0.48	0.91	1.58	0.43	0.16	0.27	-0.24	-0.10	-0.14
		(0.12)	(0.12)	(0.13)	2.43	(0.14)	(0.14)	(0.14)	(0.14)	(0.13)	(0.13)
		(0.000)	(0.000)	(0.000)	4,476	(0.003)	(0.259)	(0.045)	(0.077)	(0.456)	(0.264)
	18m	0.51	0.38	0.77	1.41	0.38	0.26	0.12			
		(0.11)	(0.11)	(0.11)	2.32	(0.13)	(0.13)	(0.13)			
		(0.000)	(0.001)	(0.000)	4,303	(0.004)	(0.044)	(0.345)			
Livestock asset value (USD)	6m	268.77	98.48	305.51	705.09	207.02	36.73	170.29	-5.12	14.84	28.74
		(76.89)	(67.45)	(75.82)	1,353.59	(75.27)	(83.42)	(78.24)	(81.27)	(67.74)	(64.02)
		(0.001)	(0.145)	(0.000)	4,418	(0.006)	(0.660)	(0.030)	(0.950)	(0.827)	(0.654)
	18m	263.66	113.33	334.24	651.68	220.92	70.59	150.33			
		(81.72)	(67.95)	(72.16)	1,280.53	(84.81)	(92.97)	(89.99)			
		(0.001)	(0.096)	(0.000)	4,263	(0.010)	(0.448)	(0.096)			
Change in livestock count (yearly, TLU)	6m	0.35	0.09	0.23	-0.28	0.15	-0.12	0.27	-0.39	-0.16	-0.33
		(0.09)	(0.10)	(0.10)	3.22	(0.09)	(0.07)	(0.08)	(0.12)	(0.13)	(0.13)
		(0.000)	(0.403)	(0.018)	4,476	(0.086)	(0.085)	(0.001)	(0.001)	(0.203)	(0.009)
	18m	-0.04	-0.08	-0.10	-0.41	-0.02	-0.06	0.04			
		(0.09)	(0.09)	(0.09)	1.83	(0.09)	(0.08)	(0.09)			
		(0.666)	(0.396)	(0.277)	4,303	(0.861)	(0.485)	(0.652)			
Livestock purchase value (yearly, USD)	6m	98.45	5.31	107.35	67.24	102.03	8.90	93.13	-89.47	-3.95	-87.41
		(10.49)	(8.97)	(10.07)	214.81	(10.04)	(11.52)	(10.84)	(12.52)	(11.49)	(12.26)
		(0.000)	(0.554)	(0.000)	4,476	(0.000)	(0.440)	(0.000)	(0.000)	(0.731)	(0.000)
	18m	8.98	1.36	19.93	48.72	18.57	10.95	7.62			
		(7.60)	(7.72)	(8.30)	190.15	(8.47)	(8.11)	(7.60)			
		(0.238)	(0.860)	(0.017)	4,303	(0.029)	(0.178)	(0.317)			
Livestock revenue (yearly, USD)	6m	7.92	22.57	46.32	138.82	23.76	38.41	-14.65	62.50	14.89	26.28
		(18.46)	(17.23)	(18.08)	382.79	(18.16)	(19.35)	(18.93)	(20.10)	(21.41)	(22.02)
		(0.668)	(0.191)	(0.011)	4,476	(0.192)	(0.048)	(0.440)	(0.002)	(0.487)	(0.233)
	18m	70.42	37.46	72.60	135.31	35.14	2.18	32.96			
		(17.55)	(17.75)	(18.21)	310.51	(19.61)	(19.24)	(19.36)			
		(0.000)	(0.036)	(0.000)	4,303	(0.074)	(0.910)	(0.090)			

Notes: Results presented are OLS estimates that include controls for randomization strata and, where possible, baseline outcomes. We assign baseline strata means to households surveyed at midline or endline but not at baseline and we control for such missing values with an indicator. See Table SI.3 for details on variable construction. Robust standard errors, clustered at the village level, and two-tailed p-values are shown in parentheses. All monetary amounts are PPP-adjusted USD terms, set at 2016 prices and deflated using Niger CPI published by the World Bank. In 2016, 1 USD = 242.553 XOF PPP. All continuous variables are winsorized at the 98th and 2th percentiles at the most disaggregated level feasible. TLU represents Tropical Livestock Units.

Supplementary Table SI.9a: Labor Participation (Household)

		Capital (Full w/o Psych.)	Psych. (Full w/o Capital)	Full	Ctrl mean/ Ctrl SD/ N	Full - Psych. (Cash grant gross ME)	Full - Capital (Psych. comp. gross ME)	Capital - Psych.	18m - 6m for Capital	18m - 6m for Psych.	18m - 6m for Full
		coef/se/p				coef/se/p			coef/se/p		
Days spent in off-farm business (HH)	6m	7.22	6.24	11.93	18.88	5.68	4.70	0.98	-1.30	-1.44	-3.31
		(1.24)	(1.37)	(1.52)	28.50	(1.59)	(1.50)	(1.40)	(1.29)	(1.42)	(1.37)
		(0.000)	(0.000)	(0.000)	4,476	(0.000)	(0.002)	(0.486)	(0.312)	(0.312)	(0.016)
	18m	5.92	4.80	8.61	18.37	3.81	2.69	1.12			
		(1.17)	(1.17)	(1.33)	25.00	(1.35)	(1.36)	(1.25)			
		(0.000)	(0.000)	(0.000)	4,303	(0.005)	(0.049)	(0.372)			
Days spent in agriculture (HH)	6m	0.75	22.22	17.65	207.95	-4.57	16.90	-21.46	-0.44	-20.58	-9.76
		(7.22)	(8.88)	(8.03)	179.29	(8.97)	(7.38)	(8.56)	(10.25)	(11.10)	(10.46)
		(0.917)	(0.013)	(0.029)	4,476	(0.611)	(0.023)	(0.013)	(0.965)	(0.064)	(0.351)
	18m	0.31	1.64	7.89	227.78	6.25	7.58	-1.33			
		(9.72)	(8.77)	(9.26)	200.96	(8.36)	(9.07)	(9.13)			
		(0.975)	(0.852)	(0.395)	4,303	(0.455)	(0.404)	(0.884)			
Days spent raising livestock (HH)	6m	5.74	3.18	9.43	44.94	6.25	3.69	2.56	1.41	-0.35	0.31
		(1.80)	(1.86)	(1.77)	40.55	(1.85)	(1.77)	(1.93)	(2.14)	(2.36)	(2.37)
		(0.002)	(0.089)	(0.000)	4,476	(0.001)	(0.038)	(0.185)	(0.508)	(0.882)	(0.896)
	18m	7.15	2.83	9.74	43.70	6.91	2.59	4.33			
		(1.71)	(1.83)	(1.87)	40.52	(2.01)	(1.82)	(1.86)			
		(0.000)	(0.123)	(0.000)	4,303	(0.001)	(0.156)	(0.021)			
Days spent in salaried employment (HH)	6m	0.21	0.17	0.31	0.76	0.13	0.10	0.03	-0.19	-0.35	-0.40
		(0.15)	(0.13)	(0.15)	3.07	(0.15)	(0.16)	(0.15)	(0.19)	(0.20)	(0.21)
		(0.156)	(0.190)	(0.041)	4,476	(0.371)	(0.529)	(0.817)	(0.315)	(0.077)	(0.058)
	18m	0.02	-0.17	-0.09	0.98	0.08	-0.11	0.19			
		(0.16)	(0.16)	(0.16)	4.16	(0.16)	(0.15)	(0.15)			
		(0.923)	(0.274)	(0.583)	4,252	(0.601)	(0.495)	(0.218)			

Notes: Results presented are OLS estimates that include controls for randomization strata and, where possible, baseline outcomes. We assign baseline strata means to households surveyed at midline or endline but not at baseline and we control for such missing values with an indicator. See Table SI.3 for details on variable construction. Robust standard errors, clustered at the village level, and two-tailed p-values are shown in parentheses.

Supplementary Table SI.9b: Labor Participation (Beneficiary)

		Capital (Full w/o Psych.)	Psych. (Full w/o Capital)	Full	Ctrl mean/ Ctrl SD/ N	Full - Psych. (Cash grant gross ME)	Full - Capital (Psych. comp. gross ME)	Capital - Psych.	18m - 6m for Capital	18m - 6m for Psych.	18m - 6m for Full
		coef/se/p				coef/se/p			coef/se/p		
Days spent in off-farm business (Benef.)	6m	4.00	3.56	4.85	6.19	1.28	0.85	0.44	-1.03	-1.37	-1.17
		(0.53)	(0.52)	(0.55)	10.72	(0.59)	(0.59)	(0.59)	(0.54)	(0.55)	(0.48)
		(0.000)	(0.000)	(0.000)	4,476	(0.030)	(0.150)	(0.461)	(0.057)	(0.014)	(0.014)
	18m	2.97	2.19	3.68	6.05	1.48	0.71	0.77			
		(0.51)	(0.51)	(0.55)	10.21	(0.60)	(0.59)	(0.57)			
		(0.000)	(0.000)	(0.000)	4,252	(0.013)	(0.229)	(0.175)			
Days spent in agriculture (Benef.)	6m	-1.34	1.27	-0.37	30.72	-1.64	0.97	-2.61	-0.31	-2.29	1.09
		(1.58)	(1.79)	(1.74)	38.84	(1.81)	(1.59)	(1.69)	(2.25)	(2.25)	(2.12)
		(0.398)	(0.479)	(0.833)	4,476	(0.365)	(0.542)	(0.123)	(0.890)	(0.308)	(0.608)
	18m	-1.65	-1.02	0.72	28.62	1.74	2.37	-0.63			
		(1.86)	(1.90)	(1.75)	37.67	(1.79)	(1.74)	(1.93)			
		(0.377)	(0.593)	(0.682)	4,252	(0.333)	(0.175)	(0.745)			
Days spent raising livestock (Benef.)	6m	3.13	1.68	3.48	16.94	1.80	0.35	1.45	0.04	-0.05	-0.62
		(0.53)	(0.61)	(0.56)	14.07	(0.60)	(0.50)	(0.58)	(0.68)	(0.77)	(0.65)
		(0.000)	(0.006)	(0.000)	4,476	(0.003)	(0.481)	(0.013)	(0.957)	(0.944)	(0.338)
	18m	3.16	1.62	2.85	14.76	1.23	-0.31	1.54			
		(0.57)	(0.63)	(0.57)	14.21	(0.61)	(0.56)	(0.63)			
		(0.000)	(0.010)	(0.000)	4,252	(0.046)	(0.578)	(0.015)			
Days spent in salaried employment (Benef.)	6m	0.10	0.15	0.18	0.21	0.03	0.08	-0.05	-0.07	-0.18	-0.08
		(0.08)	(0.08)	(0.08)	1.70	(0.09)	(0.08)	(0.09)	(0.12)	(0.11)	(0.13)
		(0.171)	(0.051)	(0.025)	4,476	(0.771)	(0.373)	(0.559)	(0.580)	(0.114)	(0.551)
	18m	0.04	-0.02	0.10	0.25	0.13	0.07	0.06			
		(0.10)	(0.08)	(0.10)	2.31	(0.10)	(0.11)	(0.09)			
		(0.699)	(0.773)	(0.285)	4,252	(0.182)	(0.534)	(0.521)			

Notes: Results presented are OLS estimates that include controls for randomization strata and, where possible, baseline outcomes. We assign baseline strata means to households surveyed at midline or endline but not at baseline and we control for such missing values with an indicator. See Table SI.3 for details on variable construction. Robust standard errors, clustered at the village level, and two-tailed p-values are shown in parentheses.

Supplementary Table SI.10a: Financial Engagement

		Capital (Full w/o Psych.)	Psych. (Full w/o Capital)	Full	Ctrl mean/ Ctrl SD/ N	Full - Psych. (Cash grant gross ME)	Full - Capital (Psych. comp. gross ME)	Capital - Psych.	18m - 6m for Capital	18m - 6m for Psych.	18m - 6m for Full
		coef/se/p				coef/se/p			coef/se/p		
Takes part in tontine/ AVEC {0,1}	6m	0.05	0.04	0.04	0.93	-0.00	-0.01	0.01	0.26	0.23	0.29
		(0.01)	(0.01)	(0.01)	0.26	(0.01)	(0.01)	(0.01)	(0.03)	(0.03)	(0.03)
		(0.000)	(0.001)	(0.001)	4,476	(0.829)	(0.114)	(0.198)	(0.000)	(0.000)	(0.000)
	18m	0.31	0.27	0.33	0.53	0.05	0.02	0.04			
		(0.03)	(0.03)	(0.03)	0.50	(0.02)	(0.02)	(0.02)			
		(0.000)	(0.000)	(0.000)	4,303	(0.013)	(0.363)	(0.106)			
Tontine/ AVEC savings (3 months, USD)	6m	0.84	-0.92	1.40	18.66	2.32	0.56	1.76	14.89	12.54	18.73
		(1.33)	(1.31)	(1.34)	24.16	(1.18)	(1.18)	(1.20)	(2.87)	(2.69)	(3.44)
		(0.525)	(0.485)	(0.297)	4,476	(0.051)	(0.637)	(0.142)	(0.000)	(0.000)	(0.000)
	18m	15.73	11.63	20.13	12.88	8.50	4.40	4.11			
		(3.04)	(2.76)	(3.53)	46.21	(3.56)	(3.73)	(3.33)			
		(0.000)	(0.000)	(0.000)	4,303	(0.017)	(0.239)	(0.218)			
Other savings (3 months, USD)	6m	1.61	1.98	3.45	3.67	1.47	1.83	-0.37	0.53	-1.44	-0.31
		(0.85)	(0.94)	(0.87)	21.75	(0.96)	(0.89)	(0.97)	(1.16)	(1.10)	(1.11)
		(0.060)	(0.036)	(0.000)	4,476	(0.128)	(0.040)	(0.706)	(0.646)	(0.193)	(0.777)
	18m	2.15	0.54	3.13	4.99	2.59	0.99	1.60			
		(0.80)	(0.73)	(0.89)	18.41	(0.91)	(0.95)	(0.82)			
		(0.008)	(0.457)	(0.001)	4,303	(0.005)	(0.300)	(0.052)			
Household asset index	6m	0.06	0.13	0.18	-0.07	0.06	0.12	-0.06	-0.02	0.01	-0.03
		(0.06)	(0.06)	(0.06)	1.31	(0.06)	(0.06)	(0.06)	(0.05)	(0.05)	(0.06)
		(0.280)	(0.027)	(0.002)	4,476	(0.380)	(0.050)	(0.279)	(0.696)	(0.892)	(0.614)
	18m	0.04	0.13	0.15	-0.07	0.02	0.11	-0.09			
		(0.06)	(0.06)	(0.06)	1.28	(0.06)	(0.05)	(0.06)			
		(0.478)	(0.020)	(0.007)	4,303	(0.731)	(0.040)	(0.097)			

Notes: Results presented are OLS estimates that include controls for randomization strata and, where possible, baseline outcomes. We assign baseline strata means to households surveyed at midline or endline but not at baseline and we control for such missing values with an indicator. See Table SI.3 for details on variable construction. Robust standard errors, clustered at the village level, and two-tailed p-values are shown in parentheses. All monetary amounts are PPP-adjusted USD terms, set at 2016 prices and deflated using Niger CPI published by the World Bank. In 2016, 1 USD = 242.553 XOF PPP. All continuous variables are winsorized at the 98th and 2th percentiles at the most disaggregated level feasible. Results from the 2nd follow-up are more reliable due to measurement issues with savings outcomes in the 1st follow-up. In the 1st follow-up survey, our data show that most of the respondents in the control group (93%) declared that they participated in a VSLA/tontine in the 1st follow-up, compared to only 53% in the 2nd follow-up. Disaggregating the participation in saving groups by tontine and VSLA (Supplementary Table SI.10b) shows that 84% of the control declare that they participated in a VSLA and 23% in a tontine in the 1st follow-up. This level of participation in a VSLA in the control at 1st follow-up appears very unlikely. It may come from the way the question was phrased in the 1st follow-up. For instance, it is possible that respondents included in this response their participation in beneficiary groups established for the parenting and child development promotion activities as part of the cash transfer program [2]. These activities were implemented consistently across all treatment and control villages in the sample we analyze in this paper. As part of these groups, beneficiaries met to discuss topics related to child nutrition and development. The activities did not cover savings or productive activities, although beneficiaries sometimes made small contributions for cooking demonstrations. The difference in results between the 1st and 2nd follow-up survey may also have been influenced by the recall period, which was 24 months in the 1st follow-up but 12 months in the 2nd follow-up, or some misunderstanding on the question during the training of surveyors, although results remain unchanged when we control for surveyor fixed effects (Supplementary Table SI.10b).

Supplementary Table SI.10b: Financial Engagement (Extensive Margins)

		Capital (Full w/o Psych.)	Psych. (Full w/o Capital)	Full	Ctrl mean/ Ctrl SD/ N	Full - Psych. (Cash grant gross ME)	Full - Capital (Psych. comp. gross ME)	Capital - Psych.	18m - 6m for Capital	18m - 6m for Psych.	18m - 6m for Full
		coef/se/p				coef/se/p			coef/se/p		
Takes part in tontine/ AVEC {0,1}	6m	0.05	0.04	0.04	0.93	-0.00	-0.01	0.01	0.26	0.23	0.29
		(0.01)	(0.01)	(0.01)	0.26	(0.01)	(0.01)	(0.01)	(0.03)	(0.03)	(0.03)
		(0.000)	(0.001)	(0.001)	4,476	(0.829)	(0.114)	(0.198)	(0.000)	(0.000)	(0.000)
	18m	0.31	0.27	0.33	0.53	0.05	0.02	0.04			
		(0.03)	(0.03)	(0.03)	0.50	(0.02)	(0.02)	(0.02)			
		(0.000)	(0.000)	(0.000)	4,303	(0.013)	(0.363)	(0.106)			
Takes part in tontine/ AVEC (enumerator F.E.)	6m	0.05	0.04	0.04	0.93	0.00	-0.01	0.01	0.26	0.23	0.29
		(0.01)	(0.01)	(0.01)	0.26	(0.01)	(0.01)	(0.01)	(0.03)	(0.03)	(0.02)
		(0.000)	(0.000)	(0.000)	4,476	(0.806)	(0.105)	(0.083)	(0.000)	(0.000)	(0.000)
	18m	0.31	0.27	0.33	0.53	0.06	0.02	0.04			
		(0.03)	(0.03)	(0.03)	0.50	(0.02)	(0.02)	(0.02)			
		(0.000)	(0.000)	(0.000)	4,303	(0.011)	(0.425)	(0.068)			
Takes part in tontine {0,1}	6m	-0.04	-0.05	-0.03	0.23	0.02	0.01	0.01	0.04	0.04	0.04
		(0.02)	(0.02)	(0.02)	0.42	(0.02)	(0.02)	(0.02)	(0.02)	(0.02)	(0.02)
		(0.046)	(0.013)	(0.115)	4,476	(0.288)	(0.607)	(0.633)	(0.100)	(0.085)	(0.140)
	18m	-0.00	-0.01	0.00	0.07	0.02	0.01	0.01			
		(0.01)	(0.01)	(0.01)	0.26	(0.01)	(0.01)	(0.01)			
		(0.802)	(0.308)	(0.842)	4,303	(0.248)	(0.663)	(0.462)			
Takes part in AVEC {0,1}	6m	0.10	0.09	0.08	0.84	-0.01	-0.02	0.01	0.24	0.21	0.28
		(0.02)	(0.02)	(0.02)	0.37	(0.01)	(0.01)	(0.01)	(0.02)	(0.03)	(0.02)
		(0.000)	(0.000)	(0.000)	4,476	(0.364)	(0.088)	(0.548)	(0.000)	(0.000)	(0.000)
	18m	0.34	0.30	0.35	0.49	0.05	0.02	0.03			
		(0.03)	(0.03)	(0.03)	0.50	(0.02)	(0.02)	(0.02)			
		(0.000)	(0.000)	(0.000)	4,303	(0.022)	(0.400)	(0.133)			

Notes: Results presented are OLS estimates that include controls for randomization strata and, where possible, baseline outcomes. We assign baseline strata means to households surveyed at midline or endline but not at baseline and we control for such missing values with an indicator. See Table SI.3 for details on variable construction. Robust standard errors, clustered at the village level, and two-tailed p-values are shown in parentheses. All monetary amounts are PPP-adjusted USD terms, set at 2016 prices and deflated using Niger CPI published by the World Bank. In 2016, 1 USD = 242.553 XOF PPP. All continuous variables are winsorized at the 98th and 2th percentiles at the most disaggregated level feasible.

Supplementary Table SI.11: Assets (Household)

		Capital (Full w/o Psych.)	Psych. (Full w/o Capital)	Full	Ctrl mean/ Ctrl SD/ N	Full - Psych. (Cash grant gross ME)	Full - Capital (Psych. comp. gross ME)	Capital - Psych.	18m - 6m for Capital	18m - 6m for Psych.	18m - 6m for Full
		coef/se/p					coef/se/p			coef/se/p	
Agricultural asset value (USD)	6m	0.87	-8.26	-0.25	187.09	8.01	-1.12	9.13	15.35	10.65	19.03
		(10.96)	(10.36)	(10.26)	244.65	(10.13)	(10.49)	(10.93)	(10.97)	(9.79)	(10.20)
		(0.937)	(0.426)	(0.981)	4,475	(0.430)	(0.915)	(0.404)	(0.162)	(0.277)	(0.062)
	18m	16.22	2.39	18.78	178.51	16.39	2.56	13.82			
		(11.18)	(10.79)	(10.42)	221.06	(10.67)	(10.51)	(11.22)			
		(0.148)	(0.825)	(0.072)	4,302	(0.126)	(0.808)	(0.219)			
Business asset value (USD)	6m	5.64	4.51	14.27	32.21	9.76	8.63	1.13	2.12	2.32	-4.53
		(4.17)	(3.88)	(3.91)	86.35	(4.29)	(4.49)	(4.54)	(4.11)	(4.26)	(4.22)
		(0.178)	(0.246)	(0.000)	4,476	(0.024)	(0.055)	(0.804)	(0.607)	(0.586)	(0.283)
	18m	7.75	6.83	9.73	30.19	2.90	1.98	0.92			
		(3.40)	(3.79)	(3.71)	77.82	(4.21)	(3.75)	(4.04)			
		(0.023)	(0.072)	(0.009)	4,303	(0.491)	(0.598)	(0.820)			

Notes: Results presented are OLS estimates that include controls for randomization strata and, where possible, baseline outcomes. We assign baseline strata means to households surveyed at midline or endline but not at baseline and we control for such missing values with an indicator. See Table SI.3 for details on variable construction. Robust standard errors, clustered at the village level, and two-tailed p-values are shown in parentheses. All monetary amounts are PPP-adjusted USD terms, set at 2016 prices and deflated using Niger CPI published by the World Bank. In 2016, 1 USD = 242.553 XOF PPP. All continuous variables are winsorized at the 98th and 2th percentiles at the most disaggregated level feasible.

Supplementary Table SI.12: Potential Mediators of Spill-Over Effects

		Capital (Full w/o Psych.)	Psych. (Full w/o Capital)	Full	Ctrl mean/ Ctrl SD/ N	Full - Psych. (Cash grant gross ME)	Full - Capital (Psych. comp. gross ME)	Capital - Psych.	18m - 6m for Capital	18m - 6m for Psych.	18m - 6m for Full
		coef/se/p				coef/se/p			coef/se/p		
Used group	6m	0.00	0.01	0.06	0.18	0.05	0.06	-0.01	0.01	-0.00	-0.02
labor on farm		(0.02)	(0.02)	(0.02)	0.38	(0.02)	(0.02)	(0.02)	(0.02)	(0.02)	(0.02)
		(0.834)	(0.514)	(0.001)	4,476	(0.009)	(0.002)	(0.688)	(0.687)	(0.922)	(0.365)
	18m	0.01	0.01	0.04	0.16	0.03	0.03	0.00			
		(0.02)	(0.02)	(0.02)	0.37	(0.02)	(0.02)	(0.02)			
		(0.458)	(0.557)	(0.029)	4,303	(0.083)	(0.125)	(0.859)			
Used hired labor	6m	0.04	0.03	0.08	0.14	0.05	0.03	0.02	-0.02	-0.01	0.01
on farm		(0.01)	(0.02)	(0.02)	0.35	(0.02)	(0.02)	(0.02)	(0.02)	(0.02)	(0.02)
		(0.003)	(0.064)	(0.000)	4,476	(0.007)	(0.060)	(0.365)	(0.327)	(0.514)	(0.565)
	18m	0.03	0.02	0.09	0.13	0.07	0.06	0.01			
		(0.02)	(0.02)	(0.02)	0.34	(0.02)	(0.02)	(0.02)			
		(0.116)	(0.293)	(0.000)	4,303	(0.000)	(0.001)	(0.609)			
Has employees	6m	0.03	0.03	0.01	0.14	-0.02	-0.02	-0.01	-0.02	0.02	0.03
in off-farm		(0.01)	(0.02)	(0.01)	0.35	(0.02)	(0.01)	(0.02)	(0.02)	(0.02)	(0.02)
activity		(0.066)	(0.024)	(0.441)	4,476	(0.126)	(0.268)	(0.641)	(0.376)	(0.426)	(0.141)
	18m	0.01	0.05	0.04	0.12	-0.01	0.03	-0.04			
		(0.01)	(0.01)	(0.01)	0.33	(0.02)	(0.01)	(0.01)			
		(0.391)	(0.000)	(0.005)	4,252	(0.509)	(0.036)	(0.005)			
Cultivated a plot	6m	-0.01	-0.01	-0.03	0.22	-0.02	-0.02	0.00	-0.01	-0.02	0.02
not owned by		(0.02)	(0.02)	(0.02)	0.42	(0.02)	(0.02)	(0.02)	(0.02)	(0.02)	(0.02)
household		(0.661)	(0.588)	(0.150)	4,476	(0.326)	(0.340)	(0.956)	(0.726)	(0.393)	(0.307)
	18m	-0.02	-0.03	-0.01	0.15	0.02	0.01	0.01			
		(0.02)	(0.02)	(0.02)	0.35	(0.01)	(0.01)	(0.01)			
		(0.298)	(0.100)	(0.665)	4,303	(0.188)	(0.509)	(0.467)			
Household	6m	-0.05	-0.00	-0.03	0.31	-0.02	0.02	-0.04	0.02	0.02	0.02
received private		(0.02)	(0.02)	(0.02)	0.46	(0.02)	(0.02)	(0.02)	(0.02)	(0.03)	(0.02)
money		(0.015)	(0.845)	(0.195)	4,476	(0.284)	(0.245)	(0.028)	(0.503)	(0.479)	(0.442)
	18m	-0.03	0.01	-0.01	0.41	-0.02	0.02	-0.04			
		(0.02)	(0.02)	(0.02)	0.49	(0.02)	(0.02)	(0.02)			
		(0.129)	(0.503)	(0.689)	4,303	(0.309)	(0.280)	(0.048)			
Household sent	6m	0.03	0.02	0.03	0.09	0.01	0.01	0.00	-0.01	0.02	0.01
private money		(0.01)	(0.01)	(0.01)	0.29	(0.01)	(0.01)	(0.01)	(0.02)	(0.02)	(0.02)
		(0.041)	(0.068)	(0.014)	4,476	(0.542)	(0.690)	(0.828)	(0.665)	(0.373)	(0.478)
	18m	0.02	0.04	0.04	0.10	0.00	0.03	-0.02			
		(0.01)	(0.01)	(0.01)	0.29	(0.01)	(0.01)	(0.01)			
		(0.186)	(0.005)	(0.002)	4,303	(0.741)	(0.077)	(0.159)			
Community	6m	-0.05	0.00	-0.01	3.63	-0.01	0.04	-0.05	0.08	0.01	0.02
tensions		(0.03)	(0.04)	(0.04)	0.74	(0.04)	(0.03)	(0.04)	(0.05)	(0.05)	(0.05)
infrequent {1-4}		(0.167)	(0.943)	(0.776)	4,476	(0.727)	(0.270)	(0.162)	(0.098)	(0.791)	(0.659)
	18m	0.03	0.02	0.01	3.50	-0.01	-0.02	0.02			
		(0.04)	(0.04)	(0.04)	0.83	(0.04)	(0.04)	(0.04)			
		(0.419)	(0.674)	(0.772)	4,160	(0.888)	(0.577)	(0.680)			

Notes: Results presented are OLS estimates that include controls for randomization strata and, where possible, baseline outcomes. We assign baseline strata means to households surveyed at midline or endline but not at baseline and we control for such missing values with an indicator. See Table SI.3 for details on variable construction. Robust standard errors, clustered at the village level, and two-tailed p-values are shown in parentheses.

Supplementary Table SI.13: Food Prices (Log Median Village Price, Weighted by Purchase Frequency)

		Capital (Full w/o Psych.)	Psych. (Full w/o Capital)	Full	Ctrl mean/ Ctrl SD/ N	Full - Psych. (Cash grant gross ME)	Full - Capital (Psych. comp. gross ME)	Capital - Psych.	18m - 6m for Capital	18m - 6m for Psych.	18m - 6m for Full
		coef/se/p				coef/se/p			coef/se/p		
Log grain prices	6m	0.01	-0.02	0.00	7.21	0.02	-0.01	0.03	-0.10	-0.00	-0.06
		(0.02)	(0.02)	(0.02)	1.57	(0.02)	(0.02)	(0.01)	(0.04)	(0.04)	(0.04)
		(0.636)	(0.324)	(0.870)	2,101	(0.125)	(0.668)	(0.036)	(0.009)	(0.995)	(0.158)
	18m	0.00	0.02	0.01	7.43	-0.01	0.00	-0.01			
		(0.01)	(0.01)	(0.01)	1.65	(0.01)	(0.01)	(0.01)			
		(0.706)	(0.208)	(0.558)	2,215	(0.550)	(0.818)	(0.399)			
Log tuber prices	6m	0.04	-0.01	0.02	5.44	0.03	-0.02	0.05	-0.03	0.02	0.04
		(0.03)	(0.03)	(0.03)	1.20	(0.03)	(0.03)	(0.03)	(0.05)	(0.05)	(0.05)
		(0.221)	(0.688)	(0.613)	1,285	(0.353)	(0.475)	(0.096)	(0.532)	(0.657)	(0.427)
	18m	0.02	0.02	0.04	5.45	0.02	0.02	-0.00			
		(0.03)	(0.03)	(0.03)	1.14	(0.03)	(0.03)	(0.03)			
		(0.559)	(0.559)	(0.236)	1,342	(0.550)	(0.489)	(0.966)			
Log vegetable prices	6m	0.03	0.00	0.03	5.09	0.03	0.00	0.02	-0.01	0.01	0.04
		(0.02)	(0.02)	(0.02)	1.37	(0.02)	(0.02)	(0.02)	(0.04)	(0.04)	(0.04)
		(0.194)	(0.832)	(0.177)	2,776	(0.250)	(0.875)	(0.282)	(0.828)	(0.818)	(0.294)
	18m	0.02	0.04	0.05	5.10	0.00	0.02	-0.02			
		(0.02)	(0.03)	(0.03)	1.32	(0.02)	(0.02)	(0.02)			
		(0.346)	(0.114)	(0.081)	2,712	(0.879)	(0.325)	(0.431)			
Log meat prices	6m	0.01	-0.04	0.02	5.97	0.07	0.01	0.06	-0.05	0.04	0.07
		(0.05)	(0.05)	(0.05)	1.41	(0.05)	(0.04)	(0.05)	(0.08)	(0.08)	(0.07)
		(0.774)	(0.406)	(0.655)	1,480	(0.155)	(0.872)	(0.260)	(0.507)	(0.593)	(0.335)
	18m	-0.03	0.01	0.07	5.94	0.06	0.10	-0.04			
		(0.05)	(0.05)	(0.05)	1.46	(0.05)	(0.05)	(0.05)			
		(0.568)	(0.829)	(0.157)	1,595	(0.223)	(0.054)	(0.437)			

Notes: Observations are log median village-level prices for food and unit, as observed in the household surveys. Results presented are OLS estimates that include controls for randomization strata and the specific type and unit of food, weighted by the number of households that report the food/unit combination in each village. Robust standard errors, clustered at the village level, and two-tailed p-values are shown in parentheses.

Supplementary Table SI.14: Mental Health Index Components

		Capital (Full w/o Psych.)	Psych. (Full w/o Capital)	Full	Ctrl mean/ Ctrl SD/ N	Full - Psych. (Cash grant gross ME)	Full - Capital (Psych. comp. gross ME)	Capital - Psych.	18m - 6m for Capital	18m - 6m for Psych.	18m - 6m for Full
		coef/se/p				coef/se/p			coef/se/p		
Mental health index	6m	0.13	0.10	0.23	0.00	0.13	0.10	0.02	0.03	0.11	0.03
		(0.04)	(0.04)	(0.04)	1.00	(0.04)	(0.04)	(0.04)	(0.05)	(0.05)	(0.05)
		(0.003)	(0.016)	(0.000)	4,476	(0.002)	(0.012)	(0.560)	(0.625)	(0.022)	(0.569)
	18m	0.15	0.21	0.26	0.00	0.04	0.10	-0.06			
		(0.04)	(0.04)	(0.04)	1.00	(0.04)	(0.04)	(0.04)			
		(0.000)	(0.000)	(0.000)	4,175	(0.247)	(0.007)	(0.149)			
Less depression {0-70}	6m	0.60	0.60	1.22	46.72	0.62	0.63	-0.00	0.89	1.09	0.84
		(0.47)	(0.47)	(0.47)	11.53	(0.49)	(0.48)	(0.50)	(0.60)	(0.58)	(0.61)
		(0.204)	(0.205)	(0.009)	4,476	(0.208)	(0.194)	(0.994)	(0.141)	(0.063)	(0.167)
	18m	1.48	1.69	2.06	45.24	0.38	0.58	-0.20			
		(0.42)	(0.43)	(0.45)	11.35	(0.46)	(0.43)	(0.43)			
		(0.000)	(0.000)	(0.000)	4,175	(0.408)	(0.178)	(0.636)			
Less disability {0-28}	6m	0.24	0.15	0.56	21.27	0.41	0.32	0.09	0.38	0.69	0.21
		(0.24)	(0.23)	(0.22)	5.47	(0.20)	(0.21)	(0.22)	(0.28)	(0.28)	(0.26)
		(0.309)	(0.516)	(0.013)	4,476	(0.041)	(0.128)	(0.671)	(0.183)	(0.013)	(0.411)
	18m	0.62	0.84	0.77	20.81	-0.07	0.16	-0.22			
		(0.21)	(0.21)	(0.19)	5.16	(0.19)	(0.19)	(0.22)			
		(0.003)	(0.000)	(0.000)	4,175	(0.731)	(0.422)	(0.306)			
Life satisfaction {1-10}	6m	0.40	0.29	0.62	5.16	0.33	0.22	0.11	-0.20	-0.02	-0.17
		(0.09)	(0.09)	(0.09)	2.07	(0.10)	(0.09)	(0.10)	(0.12)	(0.12)	(0.12)
		(0.000)	(0.002)	(0.000)	4,476	(0.001)	(0.010)	(0.260)	(0.089)	(0.881)	(0.156)
	18m	0.20	0.28	0.45	5.40	0.18	0.25	-0.07			
		(0.09)	(0.09)	(0.09)	1.90	(0.08)	(0.08)	(0.09)			
		(0.028)	(0.003)	(0.000)	4,175	(0.039)	(0.003)	(0.407)			
Inner peace {1-10}	6m	0.23	0.22	0.32	6.74	0.11	0.09	0.02	-0.02	0.04	0.02
		(0.09)	(0.09)	(0.09)	2.04	(0.09)	(0.09)	(0.09)	(0.11)	(0.10)	(0.10)
		(0.012)	(0.018)	(0.001)	4,476	(0.221)	(0.302)	(0.835)	(0.881)	(0.689)	(0.833)
	18m	0.22	0.26	0.34	6.81	0.09	0.13	-0.04			
		(0.07)	(0.07)	(0.07)	1.91	(0.07)	(0.08)	(0.08)			
		(0.003)	(0.001)	(0.000)	4,175	(0.230)	(0.092)	(0.604)			
Self-reported mental health	6m	-0.02	-0.02	0.01	0.01	0.03	0.03	0.00	0.01	0.05	-0.01
		(0.04)	(0.04)	(0.04)	1.01	(0.04)	(0.04)	(0.04)	(0.06)	(0.06)	(0.05)
		(0.644)	(0.634)	(0.739)	4,476	(0.425)	(0.427)	(0.985)	(0.926)	(0.332)	(0.924)
	18m	-0.01	0.04	0.01	-0.01	-0.03	0.02	-0.05			
		(0.04)	(0.04)	(0.04)	1.01	(0.04)	(0.04)	(0.04)			
		(0.724)	(0.357)	(0.807)	4,175	(0.485)	(0.563)	(0.245)			

Notes: Results presented are OLS estimates that include controls for randomization strata and, where possible, baseline outcomes. We assign baseline strata means to households surveyed at midline or endline but not at baseline and we control for such missing values with an indicator. See Table SI.4 for details on variable construction. Robust standard errors, clustered at the village level, and two-tailed p-values are shown in parentheses.

Supplementary Table SI.15: Self-Efficacy Index Components

		Capital (Full w/o Psych.)	Psych. (Full w/o Capital)	Full	Ctrl mean/ Ctrl SD/ N	Full - Psych. (Cash grant gross ME)	Full - Capital (Psych. comp. gross ME)	Capital - Psych.	18m - 6m for Capital	18m - 6m for Psych.	18m - 6m for Full
		coef/se/p				coef/se/p			coef/se/p		
Self efficacy index	6m	0.12	0.16	0.29	0.00	0.13	0.17	-0.04	-0.02	0.02	-0.06
		(0.04)	(0.04)	(0.04)	1.00	(0.04)	(0.04)	(0.04)	(0.05)	(0.05)	(0.05)
		(0.005)	(0.000)	(0.000)	4,476	(0.001)	(0.000)	(0.310)	(0.743)	(0.688)	(0.238)
	18m	0.10	0.18	0.23	0.00	0.04	0.12	-0.08			
		(0.04)	(0.04)	(0.04)	1.00	(0.04)	(0.04)	(0.05)			
		(0.019)	(0.000)	(0.000)	4,175	(0.297)	(0.004)	(0.089)			
You put effort to solve problems {1-4}	6m	0.12	0.14	0.24	2.87	0.10	0.12	-0.02	-0.03	-0.01	-0.08
		(0.03)	(0.03)	(0.03)	0.80	(0.03)	(0.03)	(0.03)	(0.04)	(0.04)	(0.04)
		(0.000)	(0.000)	(0.000)	4,476	(0.000)	(0.000)	(0.538)	(0.494)	(0.729)	(0.036)
	18m	0.10	0.13	0.17	2.81	0.04	0.07	-0.03			
		(0.03)	(0.03)	(0.03)	0.79	(0.03)	(0.03)	(0.04)			
		(0.004)	(0.000)	(0.000)	4,175	(0.192)	(0.021)	(0.405)			
You do what you want anyway {1-4}	6m	0.03	0.06	0.12	2.76	0.06	0.09	-0.03	0.02	0.10	0.02
		(0.03)	(0.03)	(0.03)	0.82	(0.03)	(0.03)	(0.03)	(0.04)	(0.04)	(0.04)
		(0.414)	(0.046)	(0.000)	4,476	(0.028)	(0.007)	(0.353)	(0.673)	(0.018)	(0.611)
	18m	0.05	0.16	0.14	2.74	-0.01	0.09	-0.11			
		(0.04)	(0.03)	(0.03)	0.79	(0.03)	(0.04)	(0.04)			
		(0.213)	(0.000)	(0.000)	4,175	(0.641)	(0.008)	(0.002)			
You stay on your plan and achieve goals {1-4}	6m	-0.00	0.01	0.10	3.03	0.09	0.10	-0.02	0.04	0.04	-0.02
		(0.03)	(0.03)	(0.03)	0.72	(0.02)	(0.03)	(0.03)	(0.04)	(0.04)	(0.04)
		(0.956)	(0.601)	(0.000)	4,476	(0.000)	(0.000)	(0.522)	(0.285)	(0.259)	(0.534)
	18m	0.04	0.06	0.08	2.96	0.02	0.04	-0.02			
		(0.03)	(0.03)	(0.03)	0.71	(0.03)	(0.03)	(0.03)			
		(0.165)	(0.056)	(0.007)	4,175	(0.554)	(0.209)	(0.570)			
You cope with contigencies {1-4}	6m	0.01	0.01	0.05	2.79	0.04	0.05	-0.00	0.02	0.01	-0.01
		(0.03)	(0.03)	(0.03)	0.84	(0.03)	(0.03)	(0.03)	(0.05)	(0.04)	(0.05)
		(0.801)	(0.746)	(0.116)	4,476	(0.183)	(0.179)	(0.958)	(0.684)	(0.810)	(0.785)
	18m	0.03	0.02	0.04	2.81	0.02	0.02	0.01			
		(0.03)	(0.03)	(0.03)	0.82	(0.03)	(0.03)	(0.03)			
		(0.428)	(0.527)	(0.198)	4,175	(0.495)	(0.653)	(0.848)			
You adapt and handle difficulties {1-4}	6m	0.05	0.07	0.16	2.93	0.09	0.11	-0.02	-0.05	-0.02	-0.09
		(0.03)	(0.03)	(0.03)	0.78	(0.03)	(0.03)	(0.03)	(0.04)	(0.04)	(0.04)
		(0.177)	(0.035)	(0.000)	4,476	(0.002)	(0.000)	(0.533)	(0.276)	(0.665)	(0.024)
	18m	-0.00	0.05	0.07	2.92	0.02	0.07	-0.05			
		(0.03)	(0.03)	(0.03)	0.72	(0.03)	(0.03)	(0.03)			
		(0.925)	(0.125)	(0.030)	4,175	(0.571)	(0.025)	(0.113)			
You find multiple solutions {1-4}	6m	0.11	0.15	0.16	2.81	0.01	0.06	-0.05	-0.03	-0.04	-0.02
		(0.03)	(0.03)	(0.03)	0.77	(0.03)	(0.03)	(0.03)	(0.04)	(0.04)	(0.04)
		(0.001)	(0.000)	(0.000)	4,476	(0.787)	(0.057)	(0.146)	(0.505)	(0.324)	(0.659)
	18m	0.08	0.11	0.15	2.80	0.04	0.07	-0.03			
		(0.03)	(0.03)	(0.03)	0.77	(0.03)	(0.03)	(0.03)			
		(0.016)	(0.001)	(0.000)	4,175	(0.280)	(0.033)	(0.347)			
You usually find solutions {1-4}	6m	0.09	0.09	0.14	2.84	0.05	0.04	0.01	-0.03	0.00	-0.01
		(0.03)	(0.03)	(0.03)	0.75	(0.03)	(0.03)	(0.03)	(0.04)	(0.04)	(0.04)
		(0.001)	(0.003)	(0.000)	4,476	(0.106)	(0.129)	(0.859)	(0.512)	(0.951)	(0.860)
	18m	0.06	0.09	0.13	2.85	0.04	0.06	-0.03			
		(0.03)	(0.03)	(0.03)	0.76	(0.03)	(0.03)	(0.03)			
		(0.059)	(0.006)	(0.000)	4,175	(0.219)	(0.045)	(0.417)			
You do as well as others {1-4}	6m	0.07	0.09	0.13	2.97	0.04	0.06	-0.03	-0.01	0.01	-0.01
		(0.03)	(0.03)	(0.03)	0.84	(0.03)	(0.03)	(0.03)	(0.04)	(0.04)	(0.04)
		(0.028)	(0.002)	(0.000)	4,476	(0.190)	(0.039)	(0.373)	(0.841)	(0.787)	(0.789)
	18m	0.06	0.10	0.12	2.88	0.01	0.06	-0.04			
		(0.03)	(0.03)	(0.03)	0.79	(0.03)	(0.03)	(0.03)			
		(0.063)	(0.000)	(0.000)	4,175	(0.649)	(0.082)	(0.159)			

Notes: Results presented are OLS estimates that include controls for randomization strata and, where possible, baseline outcomes. We assign baseline strata means to households surveyed at midline or endline but not at baseline and we control for such missing values with an indicator. See Table SI.4 for details on variable construction. Robust standard errors, clustered at the village level, and two-tailed p-values are shown in parentheses.

Supplementary Table SI.16: Future Expectation Index Components (kids under 30)

		Capital (Full w/o Psych.)	Psych. (Full w/o Capital)	Full	Ctrl mean/ Ctrl SD/ N	Full - Psych. (Cash grant gross ME)	Full - Capital (Psych. comp. gross ME)	Capital - Psych.	18m - 6m for Capital	18m - 6m for Psych.	18m - 6m for Full
		coef/se/p				coef/se/p			coef/se/p		
Future expectations index	6m	0.12	0.15	0.28	0.00	0.13	0.16	-0.03	-0.00	-0.01	-0.08
		(0.04)	(0.04)	(0.03)	1.00	(0.04)	(0.04)	(0.04)	(0.05)	(0.05)	(0.05)
		(0.002)	(0.000)	(0.000)	4,476	(0.001)	(0.000)	(0.437)	(0.948)	(0.920)	(0.087)
	18m	0.11	0.15	0.20	0.00	0.05	0.08	-0.03			
		(0.04)	(0.05)	(0.04)	1.00	(0.04)	(0.04)	(0.05)			
		(0.011)	(0.002)	(0.000)	4,175	(0.222)	(0.047)	(0.505)			
Expected social status	6m	0.16	0.33	0.61	6.32	0.28	0.45	-0.17	0.07	0.02	-0.15
		(0.09)	(0.09)	(0.08)	2.27	(0.08)	(0.09)	(0.10)	(0.10)	(0.11)	(0.10)
		(0.070)	(0.000)	(0.000)	4,476	(0.001)	(0.000)	(0.073)	(0.510)	(0.829)	(0.134)
	18m	0.23	0.36	0.46	6.43	0.10	0.23	-0.13			
		(0.09)	(0.10)	(0.09)	2.11	(0.09)	(0.08)	(0.10)			
		(0.015)	(0.000)	(0.000)	4,175	(0.264)	(0.006)	(0.198)			
Expected life satisfaction	6m	0.28	0.30	0.54	6.79	0.24	0.26	-0.03	-0.08	-0.02	-0.16
		(0.08)	(0.09)	(0.08)	2.02	(0.09)	(0.08)	(0.09)	(0.11)	(0.11)	(0.11)
		(0.001)	(0.001)	(0.000)	4,476	(0.007)	(0.001)	(0.779)	(0.475)	(0.881)	(0.140)
	18m	0.20	0.28	0.38	6.88	0.10	0.18	-0.09			
		(0.08)	(0.09)	(0.08)	1.87	(0.08)	(0.08)	(0.09)			
		(0.020)	(0.002)	(0.000)	4,175	(0.253)	(0.026)	(0.339)			
Expected child status (kids under 30)	6m	0.16	0.16	0.29	8.26	0.13	0.13	0.00	-0.02	-0.07	-0.15
		(0.07)	(0.07)	(0.07)	1.85	(0.07)	(0.07)	(0.07)	(0.09)	(0.09)	(0.09)
		(0.018)	(0.020)	(0.000)	4,445	(0.057)	(0.058)	(0.969)	(0.798)	(0.461)	(0.098)
	18m	0.14	0.09	0.14	7.95	0.05	0.00	0.05			
		(0.08)	(0.08)	(0.08)	1.92	(0.08)	(0.07)	(0.08)			
		(0.076)	(0.270)	(0.073)	4,162	(0.475)	(0.949)	(0.518)			

Notes: Results presented are OLS estimates that include controls for randomization strata and, where possible, baseline outcomes. We assign baseline strata means to households surveyed at midline or endline but not at baseline and we control for such missing values with an indicator. See Table SI.4 for details on variable construction. Robust standard errors, clustered at the village level, and two-tailed p-values are shown in parentheses.

Supplementary Table SI.17: Financial Support Index Components

		Capital (Full w/o Psych.)	Psych. (Full w/o Capital)	Full	Ctrl mean/ Ctrl SD/ N	Full - Psych. (Cash grant gross ME)	Full - Capital (Psych. comp. gross ME)	Capital - Psych.	18m - 6m for Capital	18m - 6m for Psych.	18m - 6m for Full
		coef/se/p				coef/se/p			coef/se/p		
Financial support index	6m	0.29	0.35	0.48	0.00	0.13	0.19	-0.06	-0.10	-0.13	-0.13
		(0.05)	(0.05)	(0.05)	1.00	(0.05)	(0.05)	(0.05)	(0.06)	(0.05)	(0.05)
		(0.000)	(0.000)	(0.000)	4,476	(0.012)	(0.000)	(0.252)	(0.084)	(0.012)	(0.014)
	18m	0.19	0.21	0.35	0.00	0.13	0.15	-0.02			
		(0.04)	(0.04)	(0.05)	1.00	(0.05)	(0.04)	(0.04)			
		(0.000)	(0.000)	(0.000)	4,252	(0.004)	(0.000)	(0.607)			
Village financial support {1-4}	6m	0.10	0.17	0.18	2.60	0.02	0.08	-0.07	-0.05	-0.10	-0.05
		(0.04)	(0.04)	(0.03)	0.87	(0.04)	(0.03)	(0.04)	(0.05)	(0.05)	(0.05)
		(0.006)	(0.000)	(0.000)	4,476	(0.657)	(0.012)	(0.087)	(0.330)	(0.038)	(0.281)
	18m	0.05	0.07	0.13	2.63	0.06	0.08	-0.02			
		(0.04)	(0.04)	(0.04)	0.87	(0.03)	(0.03)	(0.03)			
		(0.177)	(0.069)	(0.001)	4,160	(0.069)	(0.019)	(0.607)			
No. of financial supporters	6m	0.27	0.84	0.97	6.30	0.13	0.70	-0.57	-0.19	-0.44	-0.52
		(0.24)	(0.25)	(0.23)	5.93	(0.25)	(0.23)	(0.26)	(0.31)	(0.29)	(0.32)
		(0.247)	(0.001)	(0.000)	4,476	(0.604)	(0.003)	(0.031)	(0.532)	(0.130)	(0.105)
	18m	0.08	0.40	0.46	6.49	0.06	0.37	-0.32			
		(0.22)	(0.24)	(0.23)	5.64	(0.23)	(0.21)	(0.22)			
		(0.708)	(0.092)	(0.050)	4,252	(0.798)	(0.078)	(0.156)			
Fundraising probability {1-4}	6m	0.33	0.28	0.45	1.49	0.17	0.12	0.05	-0.08	-0.05	-0.08
		(0.04)	(0.04)	(0.05)	0.82	(0.05)	(0.05)	(0.05)	(0.04)	(0.05)	(0.05)
		(0.000)	(0.000)	(0.000)	4,476	(0.001)	(0.015)	(0.294)	(0.068)	(0.237)	(0.104)
	18m	0.25	0.22	0.37	1.43	0.14	0.12	0.02			
		(0.04)	(0.04)	(0.04)	0.78	(0.05)	(0.05)	(0.04)			
		(0.000)	(0.000)	(0.000)	4,252	(0.002)	(0.008)	(0.596)			

Notes: Results presented are OLS estimates that include controls for randomization strata and, where possible, baseline outcomes. We assign baseline strata means to households surveyed at midline or endline but not at baseline and we control for such missing values with an indicator. See Table SI.4 for details on variable construction. Robust standard errors, clustered at the village level, and two-tailed p-values are shown in parentheses.

Supplementary Table SI.18: Social Support Index Components

		Capital (Full w/o Psych.)	Psych. (Full w/o Capital)	Full	Ctrl mean/ Ctrl SD/ N	Full - Psych. (Cash grant gross ME)	Full - Capital (Psych. comp. gross ME)	Capital - Psych.	18m - 6m for Capital	18m - 6m for Psych.	18m - 6m for Full
		coef/se/p				coef/se/p			coef/se/p		
Social support index	6m	0.27	0.24	0.32	0.00	0.08	0.05	0.03	-0.15	-0.06	-0.14
		(0.06)	(0.04)	(0.05)	1.00	(0.05)	(0.06)	(0.06)	(0.07)	(0.06)	(0.06)
		(0.000)	(0.000)	(0.000)	4,476	(0.075)	(0.417)	(0.569)	(0.025)	(0.288)	(0.016)
	18m	0.13	0.18	0.18	0.00	0.00	0.06	-0.05			
		(0.04)	(0.05)	(0.04)	1.00	(0.05)	(0.05)	(0.05)			
		(0.004)	(0.000)	(0.000)	4,160	(0.968)	(0.217)	(0.284)			
No. of role models	6m	0.70	0.47	0.59	3.45	0.12	-0.11	0.23	-0.44	-0.28	-0.11
		(0.22)	(0.15)	(0.16)	2.79	(0.15)	(0.23)	(0.22)	(0.27)	(0.20)	(0.24)
		(0.002)	(0.002)	(0.000)	4,476	(0.415)	(0.630)	(0.284)	(0.095)	(0.165)	(0.636)
	18m	0.26	0.19	0.48	3.36	0.29	0.22	0.07			
		(0.18)	(0.17)	(0.21)	4.24	(0.20)	(0.21)	(0.18)			
		(0.162)	(0.269)	(0.021)	4,160	(0.141)	(0.287)	(0.695)			
No. of activity advisors	6m	0.31	0.34	0.47	2.22	0.12	0.16	-0.03	-0.21	-0.04	-0.19
		(0.12)	(0.09)	(0.08)	2.08	(0.09)	(0.11)	(0.13)	(0.14)	(0.15)	(0.12)
		(0.008)	(0.000)	(0.000)	4,476	(0.175)	(0.158)	(0.792)	(0.135)	(0.813)	(0.129)
	18m	0.10	0.31	0.28	2.37	-0.03	0.18	-0.20			
		(0.09)	(0.12)	(0.10)	2.33	(0.12)	(0.09)	(0.12)			
		(0.247)	(0.014)	(0.004)	4,160	(0.820)	(0.055)	(0.100)			
No. of activity mentees	6m	0.57	0.54	0.68	1.30	0.14	0.11	0.03	-0.25	-0.15	-0.25
		(0.09)	(0.09)	(0.09)	1.82	(0.10)	(0.10)	(0.10)	(0.11)	(0.12)	(0.12)
		(0.000)	(0.000)	(0.000)	4,476	(0.170)	(0.260)	(0.778)	(0.024)	(0.206)	(0.034)
	18m	0.32	0.39	0.44	1.55	0.04	0.12	-0.07			
		(0.09)	(0.09)	(0.10)	1.90	(0.11)	(0.11)	(0.10)			
		(0.000)	(0.000)	(0.000)	4,160	(0.685)	(0.284)	(0.478)			
No. of conflict advisors	6m	0.35	0.16	0.31	2.48	0.15	-0.03	0.19	-0.20	-0.01	-0.26
		(0.19)	(0.08)	(0.09)	1.90	(0.10)	(0.19)	(0.19)	(0.20)	(0.11)	(0.12)
		(0.067)	(0.053)	(0.001)	4,476	(0.112)	(0.861)	(0.336)	(0.334)	(0.903)	(0.040)
	18m	0.15	0.15	0.06	2.54	-0.09	-0.09	0.00			
		(0.09)	(0.08)	(0.08)	1.77	(0.08)	(0.09)	(0.09)			
		(0.093)	(0.071)	(0.484)	4,160	(0.286)	(0.296)	(0.964)			
No. of conflict mentees	6m	0.15	0.18	0.21	1.72	0.03	0.06	-0.03	-0.12	0.04	-0.09
		(0.08)	(0.08)	(0.09)	2.08	(0.09)	(0.09)	(0.09)	(0.12)	(0.11)	(0.12)
		(0.074)	(0.025)	(0.016)	4,476	(0.768)	(0.550)	(0.742)	(0.322)	(0.731)	(0.444)
	18m	0.03	0.22	0.12	1.79	-0.10	0.08	-0.18			
		(0.09)	(0.09)	(0.08)	2.05	(0.08)	(0.08)	(0.08)			
		(0.703)	(0.011)	(0.169)	4,160	(0.192)	(0.281)	(0.024)			
No. of market intermediaries	6m	0.05	0.14	0.18	1.54	0.04	0.13	-0.08	0.10	0.04	0.04
		(0.07)	(0.07)	(0.08)	1.72	(0.07)	(0.07)	(0.07)	(0.09)	(0.09)	(0.09)
		(0.465)	(0.056)	(0.017)	4,476	(0.523)	(0.070)	(0.205)	(0.270)	(0.711)	(0.692)
	18m	0.16	0.17	0.22	1.68	0.05	0.06	-0.01			
		(0.07)	(0.08)	(0.07)	1.60	(0.08)	(0.07)	(0.08)			
		(0.022)	(0.028)	(0.001)	4,160	(0.573)	(0.381)	(0.862)			

Notes: Results presented are OLS estimates that include controls for randomization strata and, where possible, baseline outcomes. We assign baseline strata means to households surveyed at midline or endline but not at baseline and we control for such missing values with an indicator. See Table SI.4 for details on variable construction. Robust standard errors, clustered at the village level, and two-tailed p-values are shown in parentheses.

Supplementary Table SI.19: Social Standing Index Components

		Capital (Full w/o Psych.)	Psych. (Full w/o Capital)	Full	Ctrl mean/ Ctrl SD/ N	Full - Psych. (Cash grant gross ME)	Full - Capital (Psych. comp. gross ME)	Capital - Psych.	18m - 6m for Capital	18m - 6m for Psych.	18m - 6m for Full
		coef/se/p					coef/se/p			coef/se/p	
Social standing index	6m	0.14	0.18	0.28	0.00	0.10	0.14	-0.04	-0.06	-0.05	-0.11
		(0.04)	(0.04)	(0.04)	1.00	(0.04)	(0.04)	(0.04)	(0.05)	(0.05)	(0.05)
		(0.001)	(0.000)	(0.000)	4,476	(0.015)	(0.001)	(0.350)	(0.253)	(0.335)	(0.037)
	18m	0.08	0.13	0.17	0.00	0.04	0.09	-0.05			
		(0.04)	(0.04)	(0.04)	1.00	(0.04)	(0.04)	(0.04)			
		(0.082)	(0.005)	(0.000)	4,175	(0.260)	(0.021)	(0.279)			
Good person {1-10}	6m	0.11	0.14	0.30	6.79	0.15	0.18	-0.03	-0.10	-0.03	-0.15
		(0.07)	(0.07)	(0.07)	1.87	(0.07)	(0.07)	(0.07)	(0.10)	(0.10)	(0.10)
		(0.093)	(0.053)	(0.000)	4,476	(0.039)	(0.008)	(0.682)	(0.305)	(0.765)	(0.115)
	18m	0.01	0.11	0.14	6.83	0.03	0.13	-0.10			
		(0.07)	(0.07)	(0.07)	1.69	(0.07)	(0.07)	(0.07)			
		(0.845)	(0.138)	(0.050)	4,175	(0.655)	(0.067)	(0.191)			
Respected person {1-10}	6m	0.20	0.24	0.33	6.34	0.08	0.13	-0.04	-0.17	-0.13	-0.23
		(0.08)	(0.09)	(0.08)	1.97	(0.08)	(0.07)	(0.08)	(0.10)	(0.11)	(0.10)
		(0.014)	(0.007)	(0.000)	4,476	(0.322)	(0.081)	(0.594)	(0.109)	(0.264)	(0.026)
	18m	0.03	0.12	0.10	6.53	-0.02	0.07	-0.09			
		(0.07)	(0.07)	(0.07)	1.87	(0.07)	(0.07)	(0.07)			
		(0.680)	(0.119)	(0.156)	4,175	(0.812)	(0.324)	(0.252)			
Opinion followed {1-10}	6m	0.25	0.26	0.42	5.71	0.16	0.17	-0.01	-0.08	-0.07	-0.19
		(0.09)	(0.08)	(0.08)	2.17	(0.09)	(0.09)	(0.09)	(0.11)	(0.12)	(0.11)
		(0.004)	(0.002)	(0.000)	4,476	(0.071)	(0.053)	(0.896)	(0.505)	(0.524)	(0.073)
	18m	0.18	0.19	0.23	5.94	0.04	0.05	-0.01			
		(0.09)	(0.08)	(0.08)	2.06	(0.08)	(0.08)	(0.09)			
		(0.042)	(0.026)	(0.003)	4,175	(0.596)	(0.476)	(0.877)			
Social position {1-10}	6m	0.32	0.47	0.70	4.39	0.23	0.38	-0.15	-0.06	-0.12	-0.14
		(0.10)	(0.09)	(0.09)	2.19	(0.09)	(0.09)	(0.10)	(0.11)	(0.11)	(0.11)
		(0.001)	(0.000)	(0.000)	4,476	(0.009)	(0.000)	(0.117)	(0.600)	(0.263)	(0.221)
	18m	0.26	0.34	0.56	4.81	0.22	0.30	-0.09			
		(0.10)	(0.10)	(0.09)	2.09	(0.09)	(0.09)	(0.10)			
		(0.011)	(0.001)	(0.000)	4,175	(0.022)	(0.001)	(0.409)			

Notes: Results presented are OLS estimates that include controls for randomization strata and, where possible, baseline outcomes. We assign baseline strata means to households surveyed at midline or endline but not at baseline and we control for such missing values with an indicator. See Table SI.4 for details on variable construction. Robust standard errors, clustered at the village level, and two-tailed p-values are shown in parentheses.

Supplementary Table SI.20: Social Norms Index Components

		Capital (Full w/o Psych.)	Psych. (Full w/o Capital)	Full	Ctrl mean/ Ctrl SD/ N	Full - Psych. (Cash grant gross ME)	Full - Capital (Psych. comp. gross ME)	Capital - Psych.	18m - 6m for Capital	18m - 6m for Psych.	18m - 6m for Full
		coef/se/p				coef/se/p			coef/se/p		
Social norms index	6m	0.15	0.19	0.19	0.00	-0.01	0.04	-0.05	-0.07	-0.08	-0.02
		(0.04)	(0.04)	(0.04)	1.00	(0.04)	(0.04)	(0.04)	(0.05)	(0.05)	(0.05)
		(0.001)	(0.000)	(0.000)	4,476	(0.884)	(0.334)	(0.294)	(0.201)	(0.108)	(0.727)
	18m	0.08	0.11	0.17	0.00	0.06	0.09	-0.03			
		(0.05)	(0.05)	(0.05)	1.00	(0.05)	(0.05)	(0.05)			
		(0.109)	(0.014)	(0.001)	4,160	(0.261)	(0.090)	(0.525)			
Descriptive norms index	6m	0.18	0.21	0.20	0.00	-0.01	0.02	-0.03	-0.15	-0.12	-0.06
		(0.05)	(0.05)	(0.04)	1.00	(0.05)	(0.04)	(0.05)	(0.05)	(0.05)	(0.05)
		(0.000)	(0.000)	(0.000)	4,476	(0.827)	(0.609)	(0.535)	(0.005)	(0.029)	(0.292)
	18m	0.03	0.09	0.14	0.00	0.05	0.11	-0.06			
		(0.05)	(0.05)	(0.05)	1.00	(0.06)	(0.06)	(0.06)			
		(0.543)	(0.087)	(0.008)	4,160	(0.369)	(0.045)	(0.309)			
Know women vendors {0-10}	6m	0.12	0.28	0.24	4.46	-0.04	0.12	-0.16	-0.16	-0.19	-0.09
		(0.09)	(0.10)	(0.08)	2.28	(0.10)	(0.09)	(0.11)	(0.12)	(0.12)	(0.14)
		(0.178)	(0.004)	(0.005)	4,476	(0.701)	(0.202)	(0.142)	(0.197)	(0.113)	(0.486)
	18m	-0.03	0.08	0.15	4.67	0.06	0.18	-0.12			
		(0.10)	(0.10)	(0.11)	2.19	(0.10)	(0.10)	(0.10)			
		(0.742)	(0.387)	(0.165)	4,160	(0.541)	(0.082)	(0.243)			
Know women with loans {0-10}	6m	0.17	0.32	0.33	4.15	0.01	0.16	-0.15	-0.19	-0.14	-0.16
		(0.09)	(0.09)	(0.08)	1.98	(0.09)	(0.08)	(0.10)	(0.10)	(0.10)	(0.10)
		(0.046)	(0.000)	(0.000)	4,476	(0.886)	(0.048)	(0.124)	(0.055)	(0.152)	(0.094)
	18m	-0.01	0.18	0.17	4.20	-0.01	0.18	-0.19			
		(0.07)	(0.07)	(0.08)	1.73	(0.08)	(0.08)	(0.07)			
		(0.830)	(0.014)	(0.033)	4,160	(0.927)	(0.019)	(0.009)			
Know women who started activities {0-10}	6m	0.61	0.42	0.53	4.22	0.10	-0.08	0.18	-0.40	-0.16	-0.13
		(0.12)	(0.12)	(0.12)	2.31	(0.12)	(0.11)	(0.12)	(0.12)	(0.12)	(0.13)
		(0.000)	(0.001)	(0.000)	4,476	(0.375)	(0.458)	(0.135)	(0.001)	(0.198)	(0.305)
	18m	0.21	0.27	0.40	4.14	0.13	0.18	-0.05			
		(0.11)	(0.11)	(0.11)	2.12	(0.12)	(0.12)	(0.13)			
		(0.057)	(0.019)	(0.000)	4,160	(0.260)	(0.112)	(0.684)			
Know women travel freely {0-10}	6m	0.12	0.16	0.03	3.26	-0.14	-0.09	-0.05	-0.12	-0.25	-0.02
		(0.08)	(0.08)	(0.09)	2.21	(0.09)	(0.09)	(0.09)	(0.12)	(0.12)	(0.11)
		(0.168)	(0.052)	(0.756)	4,476	(0.121)	(0.314)	(0.583)	(0.292)	(0.034)	(0.851)
	18m	-0.00	-0.08	0.01	3.56	0.09	0.01	0.08			
		(0.09)	(0.09)	(0.09)	2.02	(0.09)	(0.09)	(0.10)			
		(0.961)	(0.376)	(0.928)	4,160	(0.325)	(0.890)	(0.428)			
Prescriptive norms index	6m	0.04	0.08	0.08	0.00	0.00	0.04	-0.04	0.04	-0.01	0.02
		(0.04)	(0.04)	(0.03)	1.00	(0.04)	(0.04)	(0.04)	(0.05)	(0.05)	(0.05)
		(0.303)	(0.032)	(0.024)	4,476	(0.971)	(0.342)	(0.387)	(0.435)	(0.899)	(0.708)
	18m	0.08	0.07	0.10	0.00	0.03	0.01	0.01			
		(0.04)	(0.04)	(0.04)	1.00	(0.04)	(0.04)	(0.04)			
		(0.040)	(0.074)	(0.015)	4,160	(0.468)	(0.695)	(0.750)			
No. men who think women shd travel freely {0-10}	6m	0.06	0.08	-0.02	4.05	-0.11	-0.08	-0.03	-0.07	-0.09	0.11
		(0.11)	(0.10)	(0.10)	2.73	(0.10)	(0.11)	(0.11)	(0.15)	(0.14)	(0.14)
		(0.610)	(0.419)	(0.828)	4,476	(0.310)	(0.470)	(0.814)	(0.623)	(0.520)	(0.411)
	18m	-0.02	-0.01	0.09	4.37	0.10	0.11	-0.01			
		(0.11)	(0.10)	(0.10)	2.47	(0.10)	(0.10)	(0.11)			
		(0.886)	(0.945)	(0.360)	4,160	(0.329)	(0.303)	(0.938)			
No. men who think women shd have own work {0-10}	6m	0.17	0.32	0.24	5.71	-0.08	0.08	-0.15	0.08	-0.04	0.02
		(0.10)	(0.09)	(0.09)	2.48	(0.10)	(0.10)	(0.11)	(0.13)	(0.14)	(0.13)
		(0.083)	(0.001)	(0.008)	4,476	(0.470)	(0.455)	(0.163)	(0.574)	(0.790)	(0.893)
	18m	0.24	0.28	0.26	5.40	-0.02	0.02	-0.04			
		(0.10)	(0.09)	(0.09)	2.32	(0.09)	(0.09)	(0.09)			
		(0.012)	(0.003)	(0.005)	4,160	(0.825)	(0.828)	(0.671)			
No. women who think women shd travel freely {0-10}	6m	-0.02	-0.09	-0.04	5.49	0.05	-0.01	0.07	0.02	-0.03	0.10
		(0.11)	(0.10)	(0.10)	2.62	(0.10)	(0.11)	(0.11)	(0.13)	(0.13)	(0.12)
		(0.833)	(0.388)	(0.721)	4,476	(0.612)	(0.908)	(0.567)	(0.873)	(0.787)	(0.405)
	18m	-0.00	-0.12	0.07	5.62	0.19	0.07	0.12			
		(0.08)	(0.08)	(0.08)	2.19	(0.09)	(0.08)	(0.08)			
		(0.980)	(0.147)	(0.402)	4,160	(0.026)	(0.372)	(0.151)			
No. women who think women shd have own work {0-10}	6m	0.08	0.21	0.34	6.76	0.13	0.26	-0.13	0.22	0.09	-0.14
		(0.10)	(0.10)	(0.10)	2.55	(0.10)	(0.10)	(0.11)	(0.14)	(0.13)	(0.13)
		(0.460)	(0.037)	(0.000)	4,476	(0.165)	(0.008)	(0.218)	(0.116)	(0.517)	(0.254)
	18m	0.29	0.29	0.20	6.50	-0.10	-0.10	-0.00			
		(0.09)	(0.09)	(0.09)	2.27	(0.09)	(0.09)	(0.09)			
		(0.001)	(0.001)	(0.030)	4,160	(0.275)	(0.277)	(0.997)			

Continued on next page

Supplementary Table SI.20: Social Norms Index Components – continued from previous page

	Capital (Full w/o Psych.)	Psych. (Full w/o Capital)	Full	Ctrl mean/ Ctrl SD/ N	Full - Psych. (Cash grant gross ME)	Full - Capital (Psych. comp. gross ME)	Capital - Psych.	18m - 6m for Capital	18m - 6m for Psych.	18m - 6m for Full
	coef/se/p				coef/se/p			coef/se/p		

Notes: Results presented are OLS estimates that include controls for randomization strata and, where possible, baseline outcomes. We assign baseline strata means to households surveyed at midline or endline but not at baseline and we control for such missing values with an indicator. See Table SI.4 for details on variable construction. Robust standard errors, clustered at the village level, and two-tailed p-values are shown in parentheses.

Supplementary Table SI.21: Social Cohesion and Community Closeness Index Components

		Capital (Full w/o Psych.)	Psych. (Full w/o Capital)	Full	Ctrl mean/ Ctrl SD/ N	Full - Psych. (Cash grant gross ME)	Full - Capital (Psych. comp. gross ME)	Capital - Psych.	18m - 6m for Capital	18m - 6m for Psych.	18m - 6m for Full
		coef/se/p				coef/se/p			coef/se/p		
Social cohesion and community closeness index	6m	0.06	0.13	0.11	0.00	-0.01	0.06	-0.07	0.04	0.08	-0.02
		(0.05)	(0.05)	(0.04)	1.00	(0.05)	(0.04)	(0.05)	(0.06)	(0.06)	(0.06)
		(0.230)	(0.012)	(0.013)	4,476	(0.764)	(0.215)	(0.175)	(0.460)	(0.200)	(0.789)
	18m	0.10	0.20	0.10	0.00	-0.11	-0.00	-0.10			
		(0.05)	(0.05)	(0.05)	1.00	(0.05)	(0.04)	(0.05)			
		(0.031)	(0.000)	(0.035)	4,160	(0.021)	(0.944)	(0.032)			
Trusts village women {1-4}	6m	0.09	0.16	0.15	2.79	-0.01	0.06	-0.07	-0.01	-0.07	-0.04
		(0.04)	(0.04)	(0.03)	0.87	(0.03)	(0.03)	(0.04)	(0.04)	(0.04)	(0.05)
		(0.011)	(0.000)	(0.000)	4,476	(0.737)	(0.043)	(0.044)	(0.864)	(0.126)	(0.392)
	18m	0.08	0.10	0.11	2.82	0.02	0.03	-0.01			
		(0.03)	(0.03)	(0.03)	0.82	(0.04)	(0.03)	(0.03)			
		(0.011)	(0.005)	(0.001)	4,160	(0.620)	(0.348)	(0.690)			
No. of trusted villagers {1-10}	6m	0.04	0.01	-0.01	4.95	-0.02	-0.05	0.02	-0.01	0.07	0.11
		(0.08)	(0.09)	(0.08)	2.00	(0.09)	(0.08)	(0.09)	(0.10)	(0.10)	(0.10)
		(0.628)	(0.866)	(0.919)	4,476	(0.796)	(0.544)	(0.783)	(0.957)	(0.501)	(0.276)
	18m	0.03	0.08	0.10	4.90	0.02	0.07	-0.05			
		(0.07)	(0.07)	(0.08)	1.96	(0.08)	(0.08)	(0.08)			
		(0.632)	(0.245)	(0.180)	4,160	(0.782)	(0.385)	(0.530)			
Don't have enemies {1-4}	6m	-0.03	-0.03	0.03	2.87	0.07	0.06	0.00	0.05	0.12	-0.05
		(0.04)	(0.04)	(0.04)	0.99	(0.04)	(0.04)	(0.05)	(0.05)	(0.05)	(0.05)
		(0.472)	(0.402)	(0.445)	4,476	(0.111)	(0.144)	(0.952)	(0.323)	(0.016)	(0.291)
	18m	0.02	0.09	-0.02	2.86	-0.11	-0.04	-0.07			
		(0.04)	(0.05)	(0.05)	1.02	(0.05)	(0.05)	(0.05)			
		(0.657)	(0.058)	(0.648)	4,160	(0.029)	(0.373)	(0.158)			
Community tensions infrequent {1-4}	6m	-0.05	0.00	-0.01	3.63	-0.01	0.04	-0.05	0.08	0.01	0.02
		(0.03)	(0.04)	(0.04)	0.74	(0.04)	(0.03)	(0.04)	(0.05)	(0.05)	(0.05)
		(0.167)	(0.943)	(0.776)	4,476	(0.727)	(0.270)	(0.162)	(0.098)	(0.791)	(0.659)
	18m	0.03	0.02	0.01	3.50	-0.01	-0.02	0.02			
		(0.04)	(0.04)	(0.04)	0.83	(0.04)	(0.04)	(0.04)			
		(0.419)	(0.674)	(0.772)	4,160	(0.888)	(0.577)	(0.680)			
Community inclusiveness {1-4}	6m	0.06	0.15	0.11	2.54	-0.03	0.06	-0.09	-0.03	-0.07	-0.07
		(0.04)	(0.04)	(0.04)	0.87	(0.03)	(0.04)	(0.04)	(0.05)	(0.05)	(0.05)
		(0.156)	(0.000)	(0.001)	4,476	(0.292)	(0.120)	(0.015)	(0.485)	(0.146)	(0.164)
	18m	0.02	0.08	0.05	2.48	-0.03	0.02	-0.05			
		(0.04)	(0.04)	(0.04)	0.85	(0.04)	(0.03)	(0.03)			
		(0.506)	(0.035)	(0.223)	4,160	(0.413)	(0.512)	(0.125)			
Consider community opinions {1-4}	6m	0.05	0.09	0.07	3.07	-0.02	0.02	-0.04	0.04	0.01	0.01
		(0.03)	(0.03)	(0.03)	0.78	(0.03)	(0.03)	(0.03)	(0.04)	(0.05)	(0.04)
		(0.071)	(0.005)	(0.012)	4,444	(0.572)	(0.578)	(0.307)	(0.343)	(0.776)	(0.800)
	18m	0.09	0.10	0.08	3.01	-0.02	-0.01	-0.01			
		(0.03)	(0.03)	(0.03)	0.76	(0.03)	(0.03)	(0.03)			
		(0.002)	(0.002)	(0.014)	4,105	(0.563)	(0.718)	(0.801)			
Prefer to be diff. from community (reversed) {1-4}	6m	-0.01	-0.03	-0.09	2.40	-0.06	-0.07	0.01	0.02	0.10	0.01
		(0.04)	(0.04)	(0.04)	1.00	(0.04)	(0.04)	(0.05)	(0.06)	(0.06)	(0.05)
		(0.730)	(0.520)	(0.026)	4,437	(0.170)	(0.067)	(0.762)	(0.684)	(0.083)	(0.805)
	18m	0.01	0.07	-0.07	2.27	-0.15	-0.08	-0.06			
		(0.04)	(0.04)	(0.04)	0.89	(0.04)	(0.04)	(0.04)			
		(0.822)	(0.059)	(0.058)	4,106	(0.000)	(0.026)	(0.090)			
Selflessly care for village {1-4}	6m	0.03	0.09	0.07	2.86	-0.02	0.04	-0.06	-0.05	0.01	-0.04
		(0.04)	(0.04)	(0.04)	0.94	(0.04)	(0.04)	(0.04)	(0.05)	(0.06)	(0.05)
		(0.469)	(0.023)	(0.054)	4,404	(0.599)	(0.236)	(0.121)	(0.318)	(0.903)	(0.475)
	18m	-0.02	0.10	0.03	2.81	-0.06	0.06	-0.12			
		(0.04)	(0.04)	(0.04)	0.86	(0.04)	(0.04)	(0.04)			
		(0.499)	(0.014)	(0.366)	4,039	(0.106)	(0.096)	(0.002)			
Respect household decisions {1-4}	6m	0.03	-0.02	0.02	3.19	0.04	-0.01	0.05	0.01	0.08	0.02
		(0.03)	(0.03)	(0.03)	0.78	(0.03)	(0.03)	(0.04)	(0.05)	(0.05)	(0.04)
		(0.330)	(0.621)	(0.592)	4,462	(0.312)	(0.652)	(0.160)	(0.767)	(0.103)	(0.584)
	18m	0.05	0.06	0.04	3.01	-0.02	-0.00	-0.01			
		(0.03)	(0.03)	(0.03)	0.78	(0.03)	(0.03)	(0.03)			
		(0.165)	(0.066)	(0.218)	4,126	(0.586)	(0.912)	(0.667)			

Notes: Results presented are OLS estimates that include controls for randomization strata and, where possible, baseline outcomes. We assign baseline strata means to households surveyed at midline or endline but not at baseline and we control for such missing values with an indicator. See Table SI.4 for details on variable construction. Robust standard errors, clustered at the village level, and two-tailed p-values are shown in parentheses.

Supplementary Table SI.22: Collective Action Index Components

		Capital (Full w/o Psych.)	Psych. (Full w/o Capital)	Full	Ctrl mean/ Ctrl SD/ N	Full - Psych. (Cash grant gross ME)	Full - Capital (Psych. comp. gross ME)	Capital - Psych.	18m - 6m for Capital	18m - 6m for Psych.	18m - 6m for Full
		coef/se/p				coef/se/p			coef/se/p		
Collective action index	6m	0.30	0.34	0.42	0.00	0.08	0.12	-0.04	-0.03	-0.08	-0.07
		(0.05)	(0.05)	(0.05)	1.00	(0.05)	(0.05)	(0.05)	(0.06)	(0.06)	(0.06)
		(0.000)	(0.000)	(0.000)	4,476	(0.119)	(0.018)	(0.347)	(0.568)	(0.202)	(0.253)
	18m	0.27	0.27	0.35	0.00	0.08	0.08	0.00			
		(0.05)	(0.05)	(0.05)	1.00	(0.05)	(0.05)	(0.05)			
		(0.000)	(0.000)	(0.000)	4,160	(0.092)	(0.105)	(0.997)			
No. of associations where member	6m	0.35	0.34	0.37	0.74	0.03	0.03	0.01	-0.04	-0.05	0.04
		(0.05)	(0.05)	(0.05)	0.90	(0.05)	(0.05)	(0.05)	(0.07)	(0.08)	(0.08)
		(0.000)	(0.000)	(0.000)	4,476	(0.496)	(0.560)	(0.890)	(0.613)	(0.545)	(0.581)
	18m	0.31	0.29	0.42	0.51	0.12	0.11	0.02			
		(0.07)	(0.07)	(0.07)	1.30	(0.07)	(0.07)	(0.07)			
		(0.000)	(0.000)	(0.000)	4,160	(0.084)	(0.116)	(0.786)			
No. of association responsibilities	6m	0.06	0.10	0.11	0.17	0.01	0.05	-0.03	0.01	-0.03	-0.01
		(0.02)	(0.03)	(0.03)	0.55	(0.03)	(0.03)	(0.03)	(0.03)	(0.03)	(0.03)
		(0.007)	(0.000)	(0.000)	4,476	(0.692)	(0.113)	(0.231)	(0.708)	(0.452)	(0.696)
	18m	0.08	0.07	0.10	0.11	0.02	0.02	0.00			
		(0.02)	(0.02)	(0.02)	0.46	(0.02)	(0.02)	(0.02)			
		(0.000)	(0.000)	(0.000)	4,160	(0.226)	(0.320)	(0.852)			
Community project donations (USD)	6m	0.32	0.21	0.70	0.77	0.49	0.38	0.11	-0.22	-0.07	-0.54
		(0.14)	(0.13)	(0.15)	2.73	(0.14)	(0.14)	(0.13)	(0.15)	(0.14)	(0.16)
		(0.018)	(0.119)	(0.000)	4,476	(0.000)	(0.008)	(0.371)	(0.133)	(0.626)	(0.001)
	18m	0.10	0.14	0.17	0.19	0.02	0.06	-0.04			
		(0.05)	(0.05)	(0.06)	1.04	(0.05)	(0.06)	(0.05)			
		(0.047)	(0.006)	(0.003)	4,160	(0.651)	(0.256)	(0.453)			
Volunteering days	6m	0.19	0.36	0.26	1.13	-0.09	0.07	-0.17	-0.13	-0.30	-0.15
		(0.14)	(0.14)	(0.14)	3.64	(0.14)	(0.14)	(0.14)	(0.16)	(0.17)	(0.18)
		(0.188)	(0.009)	(0.065)	4,476	(0.492)	(0.604)	(0.232)	(0.422)	(0.077)	(0.400)
	18m	0.06	0.06	0.11	0.65	0.05	0.05	-0.00			
		(0.10)	(0.11)	(0.12)	2.45	(0.11)	(0.10)	(0.10)			
		(0.565)	(0.591)	(0.355)	4,160	(0.652)	(0.614)	(0.992)			
Works with community {1-4}	6m	0.09	0.13	0.12	2.99	-0.01	0.03	-0.04	0.00	-0.05	-0.04
		(0.03)	(0.03)	(0.03)	0.74	(0.03)	(0.03)	(0.03)	(0.04)	(0.04)	(0.04)
		(0.005)	(0.000)	(0.000)	4,476	(0.748)	(0.242)	(0.163)	(0.998)	(0.206)	(0.309)
	18m	0.09	0.07	0.08	3.02	0.01	-0.01	0.01			
		(0.03)	(0.03)	(0.03)	0.69	(0.03)	(0.03)	(0.03)			
		(0.005)	(0.022)	(0.007)	4,160	(0.853)	(0.857)	(0.730)			

Notes: Results presented are OLS estimates that include controls for randomization strata and, where possible, baseline outcomes. We assign baseline strata means to households surveyed at midline or endline but not at baseline and we control for such missing values with an indicator. See Table SI.4 for details on variable construction. Robust standard errors, clustered at the village level, and two-tailed p-values are shown in parentheses. All monetary amounts are PPP-adjusted USD terms, set at 2016 prices and deflated using Niger CPI published by the World Bank. In 2016, 1 USD = 242.553 XOF PPP. All continuous variables are winsorized at the 98th and 2th percentiles at the most disaggregated level feasible.

Supplementary Table SI.23: Intra-Household Dynamics Index Components

		Capital (Full w/o Psych.)	Psych. (Full w/o Capital)	Full	Ctrl mean/ Ctrl SD/ N	Full - Psych. (Cash grant gross ME)	Full - Capital (Psych. comp. gross ME)	Capital - Psych.	18m - 6m for Capital	18m - 6m for Psych.	18m - 6m for Full
		coef/se/p				coef/se/p			coef/se/p		
Intra-household dynamics index	6m	0.07	0.07	0.06	0.00	-0.00	-0.00	0.00	-0.05	-0.03	-0.08
		(0.04)	(0.04)	(0.04)	1.00	(0.04)	(0.04)	(0.04)	(0.06)	(0.05)	(0.06)
		(0.114)	(0.094)	(0.108)	4,476	(0.982)	(0.946)	(0.961)	(0.403)	(0.629)	(0.188)
	18m	0.02	0.04	-0.01	0.00	-0.05	-0.03	-0.02			
		(0.04)	(0.04)	(0.04)	1.00	(0.04)	(0.04)	(0.04)			
		(0.634)	(0.323)	(0.787)	4,160	(0.210)	(0.466)	(0.642)			
Partner dynamics index	6m	0.09	0.08	0.03	0.02	-0.05	-0.06	0.01	-0.07	0.04	-0.01
		(0.04)	(0.04)	(0.04)	0.99	(0.04)	(0.04)	(0.04)	(0.06)	(0.05)	(0.06)
		(0.053)	(0.063)	(0.487)	3,880	(0.242)	(0.193)	(0.793)	(0.222)	(0.448)	(0.811)
	18m	0.01	0.12	0.02	0.00	-0.10	0.00	-0.10			
		(0.05)	(0.04)	(0.04)	0.99	(0.04)	(0.04)	(0.05)			
		(0.752)	(0.007)	(0.714)	3,557	(0.017)	(0.966)	(0.024)			
Comfortable disagreeing with partner {1-4}	6m	0.06	0.06	-0.05	2.60	-0.11	-0.11	0.00	0.03	0.06	0.11
		(0.05)	(0.05)	(0.06)	1.14	(0.05)	(0.06)	(0.05)	(0.08)	(0.08)	(0.08)
		(0.266)	(0.224)	(0.367)	3,765	(0.040)	(0.059)	(0.995)	(0.676)	(0.429)	(0.160)
	18m	0.09	0.12	0.06	2.73	-0.06	-0.03	-0.03			
		(0.06)	(0.06)	(0.05)	1.17	(0.05)	(0.05)	(0.05)			
		(0.107)	(0.038)	(0.288)	3,493	(0.237)	(0.514)	(0.596)			
Trusts partner {1-4}	6m	-0.02	0.05	0.07	3.64	0.02	0.08	-0.07	0.00	-0.02	-0.08
		(0.03)	(0.03)	(0.03)	0.64	(0.03)	(0.03)	(0.03)	(0.04)	(0.03)	(0.04)
		(0.515)	(0.088)	(0.019)	3,768	(0.514)	(0.003)	(0.025)	(0.952)	(0.611)	(0.038)
	18m	-0.02	0.03	-0.01	3.69	-0.04	0.01	-0.05			
		(0.03)	(0.03)	(0.03)	0.60	(0.03)	(0.03)	(0.03)			
		(0.536)	(0.270)	(0.709)	3,490	(0.147)	(0.810)	(0.097)			
Partner inclusiveness {1-4}	6m	0.06	0.02	0.02	3.71	0.01	-0.03	0.04	-0.06	0.02	-0.02
		(0.02)	(0.02)	(0.02)	0.55	(0.02)	(0.02)	(0.02)	(0.03)	(0.03)	(0.03)
		(0.013)	(0.459)	(0.312)	3,826	(0.778)	(0.099)	(0.066)	(0.027)	(0.449)	(0.499)
	18m	-0.01	0.04	0.00	3.78	-0.04	0.01	-0.05			
		(0.02)	(0.02)	(0.02)	0.50	(0.02)	(0.02)	(0.02)			
		(0.796)	(0.065)	(0.887)	3,498	(0.084)	(0.687)	(0.035)			
Household dynamics index	6m	0.04	0.02	0.06	0.00	0.04	0.02	0.02	-0.03	-0.07	-0.09
		(0.04)	(0.04)	(0.04)	1.00	(0.04)	(0.04)	(0.04)	(0.05)	(0.05)	(0.06)
		(0.270)	(0.568)	(0.120)	4,476	(0.346)	(0.703)	(0.603)	(0.608)	(0.218)	(0.098)
	18m	0.02	-0.04	-0.03	0.00	0.01	-0.05	0.06			
		(0.04)	(0.04)	(0.04)	1.00	(0.04)	(0.04)	(0.04)			
		(0.687)	(0.307)	(0.435)	4,160	(0.798)	(0.230)	(0.151)			
Household allows family visits {0,1}	6m	0.01	-0.01	0.00	0.93	0.01	-0.01	0.02	-0.01	-0.02	-0.01
		(0.01)	(0.01)	(0.01)	0.25	(0.01)	(0.01)	(0.01)	(0.01)	(0.01)	(0.01)
		(0.343)	(0.392)	(0.764)	4,476	(0.282)	(0.549)	(0.097)	(0.378)	(0.210)	(0.436)
	18m	-0.00	-0.03	-0.01	0.91	0.02	-0.01	0.02			
		(0.01)	(0.01)	(0.01)	0.29	(0.01)	(0.01)	(0.01)			
		(0.760)	(0.041)	(0.507)	4,160	(0.145)	(0.666)	(0.051)			
Household tensions infrequent {1-4}	6m	-0.01	0.00	0.00	3.82	0.00	0.01	-0.01	0.01	-0.01	-0.00
		(0.02)	(0.02)	(0.02)	0.53	(0.02)	(0.02)	(0.02)	(0.03)	(0.03)	(0.03)
		(0.701)	(0.977)	(0.852)	4,476	(0.878)	(0.583)	(0.690)	(0.847)	(0.767)	(0.950)
	18m	-0.00	-0.01	0.00	3.76	0.01	0.00	0.01			
		(0.02)	(0.03)	(0.02)	0.58	(0.03)	(0.02)	(0.03)			
		(0.928)	(0.735)	(0.935)	4,160	(0.672)	(0.864)	(0.802)			
Household inclusiveness {1-4}	6m	0.04	0.05	0.06	3.38	0.01	0.02	-0.01	-0.01	-0.03	-0.09
		(0.03)	(0.03)	(0.03)	0.72	(0.03)	(0.03)	(0.03)	(0.04)	(0.04)	(0.04)
		(0.168)	(0.084)	(0.036)	4,476	(0.675)	(0.527)	(0.812)	(0.840)	(0.401)	(0.026)
	18m	0.04	0.02	-0.03	3.30	-0.05	-0.06	0.02			
		(0.03)	(0.03)	(0.03)	0.75	(0.03)	(0.03)	(0.03)			
		(0.202)	(0.516)	(0.348)	4,160	(0.109)	(0.026)	(0.497)			

Notes: Results presented are OLS estimates that include controls for randomization strata and, where possible, baseline outcomes. We assign baseline strata means to households surveyed at midline or endline but not at baseline and we control for such missing values with an indicator. See Table SI.4 for details on variable construction. Robust standard errors, clustered at the village level, and two-tailed p-values are shown in parentheses.

Supplementary Table SI.24: Violence Perceptions Index Components

		Capital (Full w/o Psych.)	Psych. (Full w/o Capital)	Full	Ctrl mean/ Ctrl SD/ N	Full - Psych. (Cash grant gross ME)	Full - Capital (Psych. comp. gross ME)	Capital - Psych.	18m - 6m for Capital	18m - 6m for Psych.	18m - 6m for Full
		coef/se/p				coef/se/p			coef/se/p		
Violence perceptions index	6m	-0.04	-0.04	-0.04	0.00	-0.00	0.00	-0.00	0.06	-0.04	-0.07
		(0.03)	(0.04)	(0.04)	1.00	(0.04)	(0.03)	(0.04)	(0.04)	(0.05)	(0.04)
		(0.231)	(0.317)	(0.281)	4,476	(0.971)	(0.987)	(0.957)	(0.158)	(0.430)	(0.099)
	18m	0.02	-0.08	-0.11	0.00	-0.04	-0.13	0.10			
		(0.04)	(0.04)	(0.04)	1.00	(0.04)	(0.04)	(0.04)			
		(0.593)	(0.064)	(0.008)	4,160	(0.399)	(0.003)	(0.026)			
Know women with HH-tension {0-10}	6m	0.01	0.00	-0.17	3.35	-0.17	-0.18	0.01	0.02	-0.01	0.03
		(0.10)	(0.10)	(0.11)	2.42	(0.10)	(0.09)	(0.09)	(0.13)	(0.13)	(0.13)
		(0.956)	(0.999)	(0.105)	4,476	(0.083)	(0.047)	(0.954)	(0.903)	(0.924)	(0.842)
	18m	0.02	-0.01	-0.15	3.70	-0.13	-0.17	0.03			
		(0.10)	(0.10)	(0.09)	2.29	(0.10)	(0.10)	(0.11)			
		(0.824)	(0.897)	(0.104)	4,160	(0.190)	(0.095)	(0.759)			
Women beaten for burning food {1-4}	6m	-0.05	-0.06	-0.03	1.45	0.03	0.03	0.01	0.06	-0.01	-0.03
		(0.03)	(0.03)	(0.03)	0.70	(0.03)	(0.03)	(0.03)	(0.04)	(0.04)	(0.03)
		(0.058)	(0.045)	(0.329)	4,476	(0.255)	(0.331)	(0.813)	(0.090)	(0.797)	(0.319)
	18m	0.01	-0.07	-0.06	1.48	0.01	-0.07	0.08			
		(0.03)	(0.03)	(0.03)	0.74	(0.03)	(0.03)	(0.03)			
		(0.731)	(0.010)	(0.037)	4,160	(0.779)	(0.021)	(0.006)			
Women beaten for neglecting children {1-4}	6m	-0.01	0.01	0.02	1.64	0.02	0.03	-0.01	0.03	-0.06	-0.10
		(0.03)	(0.03)	(0.03)	0.83	(0.03)	(0.03)	(0.03)	(0.04)	(0.04)	(0.05)
		(0.818)	(0.849)	(0.431)	4,476	(0.572)	(0.237)	(0.668)	(0.519)	(0.172)	(0.029)
	18m	0.02	-0.05	-0.08	1.80	-0.03	-0.10	0.07			
		(0.04)	(0.04)	(0.04)	0.91	(0.04)	(0.04)	(0.04)			
		(0.565)	(0.133)	(0.060)	4,160	(0.535)	(0.019)	(0.043)			

Notes: Results presented are OLS estimates that include controls for randomization strata and, where possible, baseline outcomes. We assign baseline strata means to households surveyed at midline or endline but not at baseline and we control for such missing values with an indicator. See Table SI.4 for details on variable construction. Robust standard errors, clustered at the village level, and two-tailed p-values are shown in parentheses.

Supplementary Table SI.25: Control Over Earnings and Productive Agency Index Components

		Capital (Full w/o Psych.)	Psych. (Full w/o Capital)	Full	Ctrl mean/ Ctrl SD/ N	Full - Psych. (Cash grant gross ME)	Full - Capital (Psych. comp. gross ME)	Capital - Psych.	18m - 6m for Capital	18m - 6m for Psych.	18m - 6m for Full
		coef/se/p				coef/se/p			coef/se/p		
Controls earnings index	6m	0.26	0.21	0.33	0.00	0.12	0.07	0.05	-0.01	-0.05	-0.07
		(0.04)	(0.04)	(0.04)	1.00	(0.04)	(0.04)	(0.04)	(0.05)	(0.05)	(0.05)
		(0.000)	(0.000)	(0.000)	4,476	(0.004)	(0.081)	(0.231)	(0.768)	(0.312)	(0.112)
	18m	0.25	0.16	0.25	0.00	0.09	0.01	0.08			
		(0.05)	(0.05)	(0.04)	1.00	(0.05)	(0.04)	(0.05)			
		(0.000)	(0.000)	(0.000)	4,252	(0.045)	(0.891)	(0.075)			
Own earnings influence {1-3}	6m	0.08	0.03	0.09	2.70	0.06	0.01	0.05	-0.07	-0.00	-0.04
		(0.02)	(0.02)	(0.02)	0.60	(0.02)	(0.02)	(0.02)	(0.03)	(0.03)	(0.04)
		(0.001)	(0.256)	(0.000)	4,042	(0.007)	(0.610)	(0.024)	(0.041)	(0.932)	(0.274)
	18m	0.01	0.02	0.05	2.78	0.03	0.04	-0.01			
		(0.02)	(0.02)	(0.02)	0.53	(0.02)	(0.02)	(0.02)			
		(0.630)	(0.274)	(0.038)	3,928	(0.278)	(0.098)	(0.546)			
Own earnings unil. power {1-3}	6m	0.00	0.04	0.05	2.15	0.01	0.04	-0.04	0.09	-0.01	0.01
		(0.04)	(0.04)	(0.04)	0.92	(0.04)	(0.04)	(0.04)	(0.05)	(0.05)	(0.05)
		(0.906)	(0.294)	(0.212)	4,068	(0.850)	(0.249)	(0.361)	(0.098)	(0.836)	(0.829)
	18m	0.09	0.03	0.06	2.36	0.03	-0.03	0.06			
		(0.04)	(0.04)	(0.04)	0.85	(0.04)	(0.04)	(0.04)			
		(0.020)	(0.440)	(0.105)	3,945	(0.414)	(0.420)	(0.132)			
Agriculture influence {1-3}	6m	0.01	0.04	0.06	2.42	0.03	0.05	-0.02	0.00	-0.03	-0.07
		(0.03)	(0.03)	(0.03)	0.73	(0.03)	(0.03)	(0.03)	(0.04)	(0.04)	(0.04)
		(0.644)	(0.255)	(0.050)	3,944	(0.446)	(0.125)	(0.497)	(0.922)	(0.523)	(0.099)
	18m	0.02	0.01	-0.01	2.61	-0.02	-0.03	0.01			
		(0.03)	(0.03)	(0.03)	0.65	(0.03)	(0.03)	(0.03)			
		(0.522)	(0.633)	(0.768)	3,862	(0.450)	(0.363)	(0.838)			
Livestock influence {1-3}	6m	0.10	0.08	0.12	2.67	0.04	0.02	0.03	-0.05	-0.04	-0.04
		(0.03)	(0.03)	(0.03)	0.63	(0.03)	(0.03)	(0.03)	(0.04)	(0.03)	(0.04)
		(0.000)	(0.005)	(0.000)	3,421	(0.105)	(0.553)	(0.344)	(0.174)	(0.259)	(0.234)
	18m	0.06	0.04	0.08	2.74	0.04	0.02	0.02			
		(0.02)	(0.03)	(0.03)	0.54	(0.03)	(0.02)	(0.03)			
		(0.025)	(0.126)	(0.002)	3,305	(0.139)	(0.337)	(0.553)			
Off-farm business influence {1-3}	6m	0.17	0.15	0.21	2.48	0.06	0.04	0.01	-0.04	-0.03	-0.05
		(0.03)	(0.04)	(0.04)	0.76	(0.04)	(0.03)	(0.03)	(0.05)	(0.04)	(0.05)
		(0.000)	(0.000)	(0.000)	3,230	(0.102)	(0.157)	(0.665)	(0.361)	(0.432)	(0.245)
	18m	0.12	0.12	0.15	2.57	0.04	0.03	0.01			
		(0.04)	(0.03)	(0.03)	0.73	(0.03)	(0.03)	(0.03)			
		(0.001)	(0.001)	(0.000)	3,113	(0.219)	(0.311)	(0.876)			
Benef. free to work {0,1}	6m	0.01	-0.00	0.01	0.88	0.02	-0.00	0.02	-0.01	-0.00	-0.02
		(0.01)	(0.01)	(0.01)	0.33	(0.01)	(0.01)	(0.01)	(0.02)	(0.02)	(0.02)
		(0.369)	(0.700)	(0.382)	4,476	(0.210)	(0.999)	(0.195)	(0.636)	(0.894)	(0.239)
	18m	0.00	-0.01	-0.01	0.89	-0.00	-0.01	0.01			
		(0.01)	(0.01)	(0.01)	0.31	(0.01)	(0.01)	(0.01)			
		(0.820)	(0.578)	(0.489)	4,160	(0.914)	(0.316)	(0.420)			
Benef. controls crop revenue {0,1}	6m	0.01	0.02	0.01	0.16	-0.01	-0.00	-0.00	-0.02	-0.01	-0.03
		(0.02)	(0.02)	(0.01)	0.37	(0.02)	(0.02)	(0.02)	(0.02)	(0.02)	(0.02)
		(0.406)	(0.242)	(0.456)	4,476	(0.650)	(0.872)	(0.807)	(0.446)	(0.583)	(0.111)
	18m	-0.00	0.01	-0.02	0.13	-0.02	-0.02	-0.01			
		(0.01)	(0.01)	(0.01)	0.33	(0.01)	(0.01)	(0.02)			
		(0.926)	(0.644)	(0.147)	4,252	(0.097)	(0.262)	(0.640)			
Beneficiary has a business {0,1}	6m	0.18	0.19	0.22	0.44	0.03	0.03	-0.00	-0.04	-0.07	-0.05
		(0.02)	(0.02)	(0.02)	0.50	(0.02)	(0.02)	(0.02)	(0.02)	(0.02)	(0.02)
		(0.000)	(0.000)	(0.000)	4,476	(0.152)	(0.118)	(0.995)	(0.068)	(0.006)	(0.019)
	18m	0.14	0.12	0.17	0.51	0.05	0.02	0.02			
		(0.02)	(0.02)	(0.02)	0.50	(0.02)	(0.02)	(0.02)			
		(0.000)	(0.000)	(0.000)	4,252	(0.054)	(0.283)	(0.390)			
Benef. owns livestock {0,1}	6m	0.16	0.06	0.16	0.59	0.11	-0.00	0.11	-0.04	0.00	-0.02
		(0.02)	(0.02)	(0.02)	0.49	(0.02)	(0.02)	(0.02)	(0.02)	(0.03)	(0.03)
		(0.000)	(0.010)	(0.000)	4,476	(0.000)	(0.961)	(0.000)	(0.130)	(0.966)	(0.511)
	18m	0.13	0.06	0.14	0.57	0.09	0.02	0.07			
		(0.02)	(0.02)	(0.02)	0.49	(0.02)	(0.02)	(0.02)			
		(0.000)	(0.013)	(0.000)	4,252	(0.000)	(0.377)	(0.003)			
Benef. controls livestock revenue {0,1}	6m	0.02	0.04	0.02	0.19	-0.03	0.00	-0.03	0.05	-0.02	0.05
		(0.02)	(0.02)	(0.02)	0.39	(0.02)	(0.02)	(0.02)	(0.02)	(0.02)	(0.02)
		(0.371)	(0.015)	(0.260)	4,476	(0.142)	(0.849)	(0.127)	(0.020)	(0.437)	(0.032)
	18m	0.07	0.03	0.07	0.21	0.04	-0.00	0.04			
		(0.02)	(0.02)	(0.02)	0.41	(0.02)	(0.02)	(0.02)			
		(0.000)	(0.171)	(0.000)	4,252	(0.027)	(0.998)	(0.029)			

Continued on next page

Supplementary Table SI.25: Control Over Earnings and Productive Agency Index Components – continued from previous page

		Capital (Full w/o Psych.)	Psych. (Full w/o Capital)	Full	Ctrl mean/ Ctrl SD/ N	Full - Psych. (Cash grant gross ME)	Full - Capital (Psych. comp. gross ME)	Capital - Psych.	18m - 6m for Capital	18m - 6m for Psych.	18m - 6m for Full
		coef/se/p				coef/se/p			coef/se/p		
Benef. traveled for work {0,1}	6m	-0.01	-0.01	-0.01	0.02	0.00	-0.00	0.00	0.01	0.01	-0.00
		(0.01)	(0.00)	(0.00)	0.15	(0.00)	(0.00)	(0.00)	(0.01)	(0.01)	(0.01)
		(0.175)	(0.058)	(0.104)	4,476	(0.774)	(0.815)	(0.614)	(0.362)	(0.173)	(0.531)
	18m	-0.00	-0.00	-0.01	0.02	-0.01	-0.01	-0.00			
		(0.01)	(0.01)	(0.00)	0.13	(0.00)	(0.00)	(0.01)			
		(0.841)	(0.978)	(0.015)	4,252	(0.018)	(0.033)	(0.864)			

Notes: Results presented are OLS estimates that include controls for randomization strata and, where possible, baseline outcomes. We assign baseline strata means to households surveyed at midline or endline but not at baseline and we control for such missing values with an indicator. See Table SI.4 for details on variable construction. Robust standard errors, clustered at the village level, and two-tailed p-values are shown in parentheses.

Supplementary Table SI.26: Control Over Household Resources Index Components

		Capital (Full w/o Psych.)	Psych. (Full w/o Capital)	Full	Ctrl mean/ Ctrl SD/ N	Full - Psych. (Cash grant gross ME)	Full - Capital (Psych. comp. gross ME)	Capital - Psych.	18m - 6m for Capital	18m - 6m for Psych.	18m - 6m for Full
		coef/se/p				coef/se/p			coef/se/p		
Controls HH	6m	0.03	0.05	0.06	0.00	0.01	0.04	-0.03	-0.04	0.00	-0.03
resources index		(0.04)	(0.04)	(0.04)	1.00	(0.04)	(0.04)	(0.04)	(0.06)	(0.06)	(0.06)
		(0.496)	(0.167)	(0.142)	4,161	(0.777)	(0.386)	(0.522)	(0.493)	(0.937)	(0.676)
	18m	-0.01	0.06	0.04	0.00	-0.02	0.05	-0.07			
		(0.05)	(0.05)	(0.05)	1.00	(0.04)	(0.04)	(0.04)			
		(0.776)	(0.234)	(0.419)	4,055	(0.664)	(0.218)	(0.110)			
Daily spending	6m	0.03	0.04	0.05	2.63	0.01	0.03	-0.01	-0.01	0.00	-0.01
influence {1-3}		(0.02)	(0.02)	(0.02)	0.60	(0.02)	(0.02)	(0.02)	(0.04)	(0.04)	(0.04)
		(0.289)	(0.099)	(0.036)	4,078	(0.571)	(0.273)	(0.589)	(0.777)	(0.926)	(0.894)
	18m	0.02	0.04	0.05	2.63	0.00	0.03	-0.03			
		(0.03)	(0.03)	(0.03)	0.63	(0.03)	(0.03)	(0.03)			
		(0.608)	(0.134)	(0.100)	4,006	(0.859)	(0.244)	(0.328)			
Daily spending	6m	0.00	0.05	0.04	2.12	-0.01	0.03	-0.04	0.03	0.02	0.00
unil. power		(0.03)	(0.04)	(0.04)	0.90	(0.04)	(0.04)	(0.04)	(0.05)	(0.04)	(0.05)
{1-3}		(0.893)	(0.180)	(0.350)	4,094	(0.747)	(0.402)	(0.230)	(0.602)	(0.710)	(0.961)
	18m	0.03	0.07	0.04	2.27	-0.03	0.01	-0.04			
		(0.04)	(0.04)	(0.03)	0.86	(0.03)	(0.03)	(0.04)			
		(0.432)	(0.070)	(0.270)	4,016	(0.419)	(0.811)	(0.342)			
Large purchases	6m	0.05	0.06	0.07	2.50	0.01	0.03	-0.02	-0.02	-0.01	-0.03
influence {1-3}		(0.03)	(0.03)	(0.03)	0.71	(0.03)	(0.03)	(0.03)	(0.04)	(0.04)	(0.04)
		(0.125)	(0.032)	(0.012)	4,079	(0.656)	(0.301)	(0.558)	(0.564)	(0.761)	(0.495)
	18m	0.02	0.05	0.05	2.50	-0.00	0.03	-0.03			
		(0.03)	(0.03)	(0.03)	0.73	(0.03)	(0.03)	(0.03)			
		(0.512)	(0.130)	(0.164)	4,010	(0.901)	(0.394)	(0.327)			
Large purchases	6m	-0.02	0.03	0.01	1.97	-0.02	0.03	-0.05	0.04	0.04	0.03
unil. power		(0.04)	(0.04)	(0.04)	0.91	(0.04)	(0.04)	(0.04)	(0.05)	(0.05)	(0.05)
{1-3}		(0.622)	(0.429)	(0.727)	4,090	(0.684)	(0.410)	(0.220)	(0.341)	(0.421)	(0.521)
	18m	0.03	0.07	0.05	2.08	-0.02	0.02	-0.04			
		(0.04)	(0.04)	(0.04)	0.90	(0.03)	(0.03)	(0.04)			
		(0.486)	(0.075)	(0.206)	4,006	(0.519)	(0.540)	(0.245)			
Family planning	6m	0.07	0.02	0.00	2.32	-0.01	-0.07	0.05	-0.10	0.01	-0.08
influence {1-3}		(0.04)	(0.04)	(0.04)	0.82	(0.04)	(0.04)	(0.04)	(0.06)	(0.06)	(0.06)
		(0.082)	(0.699)	(0.931)	2,730	(0.770)	(0.103)	(0.202)	(0.085)	(0.912)	(0.185)
	18m	-0.03	0.02	-0.08	2.28	-0.10	-0.05	-0.06			
		(0.05)	(0.05)	(0.05)	0.84	(0.05)	(0.05)	(0.05)			
		(0.504)	(0.626)	(0.123)	2,510	(0.032)	(0.341)	(0.232)			
Family planning	6m	0.01	-0.04	-0.05	1.68	-0.01	-0.06	0.05	-0.08	0.00	0.01
unil. power		(0.04)	(0.05)	(0.05)	0.87	(0.05)	(0.05)	(0.05)	(0.06)	(0.06)	(0.06)
{1-3}		(0.806)	(0.359)	(0.295)	2,740	(0.889)	(0.213)	(0.280)	(0.175)	(0.956)	(0.903)
	18m	-0.07	-0.04	-0.04	1.82	-0.00	0.03	-0.03			
		(0.05)	(0.04)	(0.04)	0.90	(0.04)	(0.04)	(0.05)			
		(0.125)	(0.370)	(0.352)	2,539	(0.950)	(0.484)	(0.459)			
Own healthcare	6m	0.00	-0.02	0.01	2.84	0.03	0.01	0.02	-0.01	0.02	-0.01
influence {1-3}		(0.02)	(0.02)	(0.02)	0.43	(0.02)	(0.02)	(0.02)	(0.03)	(0.03)	(0.03)
		(0.879)	(0.301)	(0.544)	4,171	(0.088)	(0.600)	(0.204)	(0.719)	(0.402)	(0.605)
	18m	-0.01	0.00	-0.00	2.84	-0.01	0.00	-0.01			
		(0.02)	(0.02)	(0.02)	0.41	(0.02)	(0.02)	(0.02)			
		(0.714)	(0.850)	(0.899)	4,106	(0.766)	(0.802)	(0.624)			
Own healthcare	6m	-0.03	0.02	0.01	2.25	-0.01	0.05	-0.06	0.04	0.03	0.04
unil. power		(0.04)	(0.04)	(0.04)	0.90	(0.04)	(0.04)	(0.04)	(0.05)	(0.05)	(0.05)
{1-3}		(0.331)	(0.529)	(0.743)	4,198	(0.794)	(0.240)	(0.152)	(0.397)	(0.622)	(0.424)
	18m	0.01	0.05	0.05	2.34	0.01	0.05	-0.04			
		(0.03)	(0.04)	(0.03)	0.85	(0.03)	(0.03)	(0.03)			
		(0.830)	(0.189)	(0.102)	4,111	(0.836)	(0.113)	(0.244)			
Partner's	6m	-0.01	0.05	0.03	2.25	-0.02	0.04	-0.06	-0.01	-0.01	-0.02
earnings		(0.03)	(0.03)	(0.04)	0.82	(0.04)	(0.04)	(0.04)	(0.04)	(0.04)	(0.05)
influence {1-3}		(0.760)	(0.165)	(0.388)	3,843	(0.648)	(0.249)	(0.099)	(0.770)	(0.860)	(0.666)
	18m	-0.02	0.04	0.01	2.32	-0.03	0.03	-0.06			
		(0.03)	(0.03)	(0.04)	0.76	(0.04)	(0.03)	(0.03)			
		(0.506)	(0.253)	(0.770)	3,627	(0.412)	(0.339)	(0.066)			
Child education	6m	0.01	0.01	0.03	2.80	0.02	0.02	-0.00	-0.03	-0.03	-0.02
influence {1-3}		(0.02)	(0.02)	(0.02)	0.49	(0.02)	(0.02)	(0.02)	(0.03)	(0.03)	(0.03)
		(0.681)	(0.677)	(0.245)	3,596	(0.469)	(0.428)	(0.983)	(0.374)	(0.363)	(0.456)
	18m	-0.02	-0.02	0.00	2.86	0.02	0.02	0.00			
		(0.02)	(0.02)	(0.02)	0.41	(0.02)	(0.02)	(0.02)			
		(0.424)	(0.377)	(0.924)	3,520	(0.335)	(0.381)	(0.920)			

Continued on next page

Supplementary Table SI.26: Control Over Household Resources Index Components – continued from previous page

	Capital (Full w/o Psych.)	Psych. (Full w/o Capital)	Full	Ctrl mean/ Ctrl SD/ N	Full - Psych. (Cash grant gross ME)	Full - Capital (Psych. comp. gross ME)	Capital - Psych.	18m - 6m for Capital	18m - 6m for Psych.	18m - 6m for Full
	coef/se/p				coef/se/p			coef/se/p		

Notes: Results presented are OLS estimates that include controls for randomization strata and, where possible, baseline outcomes. We assign baseline strata means to households surveyed at midline or endline but not at baseline and we control for such missing values with an indicator. See Table SI.4 for details on variable construction. Robust standard errors, clustered at the village level, and two-tailed p-values are shown in parentheses.

Supplementary Table SI.27: Cost-benefit analysis (assuming linear growth post-intervention)

		Capital	Psychosocial	Full
**Panel 1: Program costs per beneficiary, USD PPP 2016**				
	Program administration	78	78	78
	Identification, Communication	3	3	3
	Measure 0. Group formation	1	1	1
	Measure 1. Coaching	9	9	9
	Measure 2. Community sensitization on aspirations and social norms	0	38	38
	Measure 3. Facilitation of community savings and loan groups	2	2	2
	Measure 4. Life-skills training	0	64	64
	Measure 5. Micro-entrepreneurship training	65	65	65
	Measure 6. Cash grants	321	0	321
	Measure 7. Facilitation of market and information access	4	4	4
	Total costs, calculated as if all incurred immediately at beginning of year 0	482	263	584
(1)	Total costs, inflated to year 2 at 5% annual discount rate	531	290	644
**Panel 2: Benefits per household, USD PPP 2016, all values inflated to year 2 at 5% annual social discount rate**				
(2)	Year 1 gross consumption treatment effect (linear growth for months 1-6)	93	54	153
(3)	Year 2 gross consumption treatment effect (linear growth for months 7-18)	294	311	553
(4)	B1: Year 3 onward gross consumption treatment effect, assumed dissipation of 75%	92	97	173
	B2: Year 3 onward gross consumption treatment effect, assumed dissipation of 50%	267	282	503
	B3: Year 3 onward gross consumption treatment effect, assumed dissipation of 25%	734	777	1382
(5)	C: Year 3 onward gross consumption treatment effect, assuming year 2 gains persist in perpetuity	5595	5918	10533
(6)	A: Total benefits: (2) + (3) = (6) using 5%, no impact after year 2	387	365	706
(7)	B1: Total benefits: (2) + (3) + (4) = (7) 5% discount rate, 75% annual dissipation	479	462	879
	B2: Total benefits using 5% discount rate, 50% annual dissipation	654	647	1208
	B3: Total benefits using 5% discount rate, 25% annual dissipation	1122	1141	2088
(8)	C: Total benefits: (2) + (3) + (5) = (8) using 5% discount rate, assuming year 2 gains persist in perpetuity	5983	6283	11239
**Panel 3: Benefit/cost ratios**				
(9)	A: Total benefits/total costs ratio: (6) / (1) = (9) at 5% discount rate	73%	126%	110%
	A: Benefit/cost ratio, at discount rate of 7%	72%	123%	108%
	A: Benefit/cost ratio, at discount rate of 10%	70%	120%	105%
(10)	B1: Total Benefits/Total costs: (7) / (1) = (10), 5% discount, 75% annual dissipation	90%	159%	137%
	B2: Benefit/cost ratio using 5% discount rate, 50% annual dissipation	123%	223%	188%
	B3: Benefit/cost ratio using 5% discount rate, 25% annual dissipation	211%	394%	324%
(11)	C: Total benefits/total costs ratio: (8) / (1) = (11) using 5% discount rate, assuming year 2 gains persist in perpetuity	1126%	2169%	1746%
(12)	Real internal rate of return (IRR)			
	A: Assuming dissipation of 100% after year 2	-12%	19%	11%
	B1: Assuming annual dissipation of 75%	0%	31%	22%
	B2: Assuming annual dissipation of 50%	14%	45%	36%
	B3: Assuming annual dissipation of 25%	30%	60%	50%
	C: Assuming effects are sustained in perpetuity	47%	76%	67%

Notes: We use 98% winsorized consumption estimates in our benefits calculation. Note that we measure benefits 6 months after the intervention on average in year 1 and 18 months on average in year 2. All monetary amounts are PPP-adjusted USD terms, set at 2016 prices and deflated using Niger CPI published by the World Bank. In 2016, 1 USD = 242.553 XOF PPP. We assume linear growth of impacts by month post-intervention. In nominal terms, in 2016, 1 US dollar = 592.445 XOF. We consider the costs to be incurred in 2018, with an inflation rate of 5.85%. Hence 10,000 XOF in 2018 = 10000 / (592.4 * 1.0585) = $15.95 in 2016. Differences in benefit-cost ratios (row 9) between Psychosocial and Full: 1.259-1.097=0.162, s.e=0.362, p=0.654; between Capital and Full: 0.729-1.097=-0.368, s.e.=0.196, p=0.060; and between Capital and Psychosocial: 0.729-1.259=-0.530, s.e=0.348, p=0.127. Consistent results are found using other scenarios.

Supplementary Table SI.28: Costs and Psychosocial Effects

Treatment group	Capital	Psychosocial	Full
Year 0 cost per beneficiary (USD 2016)	482	263	584
Life satisfaction (year 2)			
Treatment effect in standard deviations	0.107	0.145	0.237
Cost per 0.1 standard deviations (USD 2016)	451	181	246
Depression (years 1 & 2)			
No. of depression cases averted	25	68	87
Cost per case of depression averted (USD 2016)	20,681	4,028	7,422

Notes: Depressive symptoms were assessed with the CES-D-10 screening tool [52] and a score of 13 or more on a 0-30 point scale was considered high risk for depression [62]. We use the benchmark of 0.10 SD given it is the meta-analytic effect of economic interventions on psychological well-being [63].

Supplementary Table SI.29: Heterogeneity by Baseline Consumption

	18 months later	
	(1)<br>Gross consumption (daily, USD/adult eq.)<br>b/se/p	(2)<br>Mental health index<br>b/se/p
Capital	0.18	0.11
	(0.06)	(0.06)
	(0.004)	(0.072)
Psychosocial	0.23	0.18
	(0.07)	(0.06)
	(0.001)	(0.002)
Full	0.39	0.26
	(0.07)	(0.06)
	(0.000)	(0.000)
Household consumption below median @ baseline	0.08	-0.11
	(0.07)	(0.06)
	(0.247)	(0.077)
Capital × Household consumption below median @ baseline	-0.12	0.10
	(0.08)	(0.09)
	(0.133)	(0.229)
Psychosocial × Household consumption below median @ baseline	-0.10	0.07
	(0.09)	(0.08)
	(0.222)	(0.356)
Full × Household consumption below median @ baseline	-0.27	0.03
	(0.08)	(0.09)
	(0.001)	(0.715)
Observations	4167	4072
Control mean @ followup above median	1.78	0.05
Control mean @ followup below median	1.60	-0.05

Notes: Results presented are OLS estimates that include controls for randomization strata and, where possible, baseline outcomes. We assign baseline strata means to households surveyed at endline but not at baseline and we control for such missing values with an indicator. Robust standard errors, clustered at the village level, and two-tailed p-values are shown in parentheses. *** $p < 0.01$, ** $p < 0.05$, * $p < 0.1$. All monetary amounts are PPP-adjusted USD terms, set at 2016 prices and deflated using Niger CPI published by the World Bank. In 2016, 1 USD = 242.553 XOF PPP. All continuous variables are winsorized at the 98th and 2th percentiles at the most disaggregated level feasible. See Tables SI.3 and SI.4 for details on variable construction.

Supplementary Table SI.30: Heterogeneity by Baseline Mental Health Index

	18 months later	
	(1) Gross consumption (daily, USD/adult eq.) b/se/p	(2) Mental health index b/se/p
Capital	0.17	0.23
	(0.06)	(0.05)
	(0.007)	(0.000)
Psychosocial	0.19	0.25
	(0.07)	(0.06)
	(0.009)	(0.000)
Full	0.29	0.35
	(0.07)	(0.06)
	(0.000)	(0.000)
Beneficiary's mental health below median @ baseline	-0.00	0.04
	(0.06)	(0.07)
	(0.943)	(0.600)
Capital × Beneficiary's mental health below median @ baseline	-0.10	-0.14
	(0.09)	(0.09)
	(0.245)	(0.116)
Psychosocial × Beneficiary's mental health below median @ baseline	-0.00	-0.07
	(0.09)	(0.09)
	(0.959)	(0.435)
Full × Beneficiary's mental health below median @ baseline	-0.08	-0.18
	(0.09)	(0.08)
	(0.369)	(0.037)
Observations	4167	4105
Control mean @ followup above median	1.71	0.10
Control mean @ followup below median	1.69	-0.11

Notes: Results presented are OLS estimates that include controls for randomization strata and, where possible, baseline outcomes. We assign baseline strata means to households surveyed at endline but not at baseline and we control for such missing values with an indicator. Robust standard errors, clustered at the village level, and two-tailed p-values are shown in parentheses. *** $p < 0.01$, ** $p < 0.05$, * $p < 0.1$. All monetary amounts are PPP-adjusted USD terms, set at 2016 prices and deflated using Niger CPI published by the World Bank. In 2016, 1 USD = 242.553 XOF PPP. All continuous variables are winsorized at the 98th and 2th percentiles at the most disaggregated level feasible. See Tables SI.3 and SI.4 for details on variable construction.

Supplementary Information References

1. World Bank. *Republic of Niger Priorities for Ending Poverty and Boosting Shared Prosperity : Systematic Country Diagnostic* 2017. https://openknowledge.worldbank.org/handle/10986/28994https://openknowledge.worldbank.org/bitstream/handle/10986/28994/NIGER-SCD-12012017.pdf.
2. Premand, P. & Barry, O. *Behavioral Change Promotion, Cash Transfers and Early Childhood Development: Experimental Evidence from a Government Program in a Low-Income Setting* 2020. https://openknowledge.worldbank.org/handle/10986/34385.
3. Premand, P. & Schnitzer, P. Efficiency, legitimacy, and impacts of targeting methods: Evidence from an experiment in Niger. *The World Bank Economic Review* **35,** 892–920 (2021).
4. Archibald, E., Bossuroy, T. & Premand, P. Productive Inclusion Measures and Adaptive Social Protection in the Sahel: A Case Study. http://documents1.worldbank.org/curated/en/945281604463808844/pdf/A-Case-Study.pdf (2020).
5. Markus, H. R. & Kitayama, S. Cultures and Selves: A Cycle of Mutual Constitution. *Perspectives on psychological science* **5.** Publisher: Sage Publications Sage CA: Los Angeles, CA, 420–430 (2010).
6. Lewin, K. in *Readings in social psychology* 459–473 (1952).
7. Walton, G. M. & Yeager, D. S. Seed and Soil: Psychological Affordances in Contexts Help to Explain Where Wise Interventions Succeed or Fail. *Current Directions in Psychological Science* **29,** 219–226 (2020).
8. Gelfand, M. J., Raver, J. L., Nishii, L., Leslie, L. M., Lun, J., Lim, B. C., Duan, L., Almaliach, A., Ang, S. & Arnadottir, J. Differences between Tight and Loose Cultures: A 33-Nation Study. *Science* **332,** 1100–1104 (2011).
9. Hofstede, G. *Culture's Consequences: Comparing Values, Behaviors, Institutions, and Organizations across Nations* http://www.vlebooks.com/vleweb/product/openreader?id=none&isbn=9781452207933 (Sage, Thousand Oaks, Calif.; London, 2001).
10. Leung, A. K.-Y. & Cohen, D. Within-and between-Culture Variation: Individual Differences and the Cultural Logics of Honor, Face, and Dignity Cultures. *Journal of personality and social psychology* **100.** Publisher: American Psychological Association, 507 (2011).
11. Markus, H. R. & Conner, A. *Clash!: How to Thrive in a Multicultural World* 320 pp. (Penguin, 2014).
12. Markus, H. R. What Moves People to Action? Culture and Motivation. *Current Opinion in Psychology. Culture* **8,** 161–166 (2016).
13. Riemer, H., Shavitt, S., Koo, M. & Markus, H. R. Preferences Don't Have to Be Personal: Expanding Attitude Theorizing With a Cross-Cultural Perspective. *Psychological review* **121,** 619 (2014).
14. Campos, F., Frese, M., Goldstein, M., Iacovone, L., Johnson, H. C., McKenzie, D. & Mensmann, M. Teaching personal initiative beats traditional training in boosting small business in West Africa. *Science* **357,** 1287–1290 (2017).
15. Feinberg, M. & Willer, R. From Gulf to Bridge: When Do Moral Arguments Facilitate Political Influence? *Personality and Social Psychology Bulletin* **41,** 1665–1681 (2015).
16. Stephens, N. M., Fryberg, S. A., Markus, H. R., Johnson, C. S. & Covarrubias, R. Unseen Disadvantage: How American Universities' Focus on Independence Undermines the Academic Performance of First-Generation College Students. *Journal of Personality and Social Psychology* **102.** Publisher: American Psychological Association, 1178 (2012).
17. Thomas, C. C. & Markus, H. R. *Enculturating Development Science: Beyond the WEIRD Independent Paradigm* Mimeo. Forthcoming.
18. Thomas, C. C., Otis, N. G., Abraham, J. R., Markus, H. R. & Walton, G. M. Toward a Science of Delivering Aid with Dignity: Experimental Evidence and Local Forecasts from Kenya. *Proceedings of the National Academy of Sciences* **117,** 15546–15553 (2020).

19. Bandura, A. Social Cognitive Theory of Mass Communication. *Media Psychology* **3,** 265–299 (2001).

20. Paluck, E. L. What's in a Norm? Sources and Processes of Norm Change. *Journal of Personality and Social Psychology* **96,** 594–600 (2009).

21. Paluck, E. L. & Shepherd, H. The Salience of Social Referents: A Field Experiment on Collective Norms and Harassment Behavior in a School Social Network. *Journal of Personality and Social Psychology* **103,** 899–915 (2012).

22. San Martin, A., Sinaceur, M., Madi, A., Tompson, S., Maddux, W. W. & Kitayama, S. Self-Assertive Interdependence in Arab Culture. *Nature Human Behaviour* **2.** Publisher: Nature Publishing Group, 830–837 (2018).

23. Slater, M. D. & Rouner, D. Entertainment—Education and Elaboration Likelihood: Understanding the Processing of Narrative Persuasion. *Communication Theory* **12,** 173–191 (2002).

24. Oettingen, G. *Rethinking Positive Thinking: Inside the New Science of Motivation* 219 pp. (Current, New York, NY, 2014).

25. Acevedo, P., Cruces, G., Gertler, P. & Martinez, S. Living Up to Expectations: How Job Training Made Women Better Off and Men Worse Off. *NBER Working Paper No. 23264* (2017).

26. Adhvaryu, A., Kala, N. & Nyshadham, A. The Skills to Pay the Bills: Returns to On-the-job Soft Skills Training. *NBER Working Paper No. 24313.* `http://www.nber.org/papers/w24313.pdf` (2018).

27. Blattman, C., Jamison, J. C. & Sheridan, M. Reducing Crime and Violence: Experimental Evidence From Cognitive Behavioral Therapy in Liberia. *American Economic Review* **107,** 1165–1206 (2017).

28. Ashraf, N., Bau, N., Low, C. & McGinn, K. Negotiating a Better Future: How Interpersonal Skills Facilitate Intergenerational Investment. *The Quarterly Journal of Economics* **135,** 1095–1151 (2020).

29. Heckman, J., Stixrud, J. & Urzua, S. The Effects of Cognitive and Noncognitive Abilities on Labor Market Outcomes and Social Behavior. *NBER Working Paper No. 12006.* `http://www.nber.org/papers/w12006.pdf` (2006).

30. Barrera-Osorio, F., Kugler, A. & Silliman, M. Hard and Soft Skills in Vocational Training: Experimental Evidence from Colombia. *NBER Working Paper No. 27548* (2020).

31. Knowles, M. S. *Andragogy in Action: Applying Modern Principles of Adult Learning* (Jossey Bass, San Francisco, CA, 1984).

32. Esopo, K., Mellow, D., Thomas, C., Uckat, H., Abraham, J., Jain, P., Jang, C., Otis, N., Riis-Vestergaard, M., Starcev, A., *et al.* Measuring self-efficacy, executive function, and temporal discounting in Kenya. *Behaviour Research and Therapy* **101,** 30–45 (2018).

33. Laajaj, R., Macours, K., Hernandez, D. A. P., Arias, O., Gosling, S. D., Potter, J., Rubio-Codina, M. & Vakis, R. Challenges to capture the big five personality traits in non-WEIRD populations. *Science advances* **5,** eaaw5226 (2019).

34. Operario, D., Adler, N. E. & Williams, D. R. Subjective Social Status: Reliability and Predictive Utility for Global Health. *Psychology & health* **19,** 237–246 (2004).

35. Wuepper, D. & Lybbert, T. J. Perceived Self-Efficacy, Poverty, and Economic Development. *Annual Review of Resource Economics* **9,** 383–404 (2017).

36. Dalton, P. S., Ghosal, S. & Mani, A. Poverty and Aspirations Failure. *The Economic Journal* **126,** 165–188 (2016).

37. Duflo, E. *Human Values and the Design of the Fight Against Poverty* Tanner Lectures. Harvard University, 2012.

38. Ridley, M., Rao, G., Schilbach, F. & Patel, V. Poverty, Depression, and Anxiety: Causal Evidence and Mechanisms. *Science* **370.** `https://www.sciencemag.org/lookup/doi/10.1126/science.aay0214` (2020).

39. Thomas, C. C., Rathod, S., De Silva, M., Weiss, H. & Patel, V. The 12-Item WHO Disability Assessment Schedule II as an Outcome Measure for Treatment of Common Mental Disorders. *Global Mental Health* **3,** e14 (2016).

40. Ali, G.-C., Ryan, G. & De Silva, M. J. Validated Screening Tools for Common Mental Disorders in Low and Middle Income Countries: A Systematic Review. *PLOS ONE* **11,** e0156939 (2016).

41. Osei-Tutu, A., Dzokoto, V. A., Affram, A. A., Adams, G., Norberg, J. & Doosje, B. Cultural Models of Well-Being Implicit in Four Ghanaian Languages. *Frontiers in Psychology* **11,** 1798 (2020).

42. Bandura, A. *Self-Efficacy: The Exercise of Control* 604 pp. (W.H. Freeman, New York, 1997).

43. Delavande, A., Giné, X. & McKenzie, D. Measuring Subjective Expectations in Developing Countries: A Critical Review and New Evidence. *Journal of development economics* **94,** 151–163 (2011).

44. Woolcock, M. & Narayan, D. Social Capital: Implications for Development Theory, Research, and Policy. *The World Bank Research Observer* **15,** 225–249 (2000).

45. Akyeampong, E., Bates, R. H., Nunn, N. & Robinson, J. *Africa's Development in Historical Perspective* (Cambridge University Press, 2014).

46. Hitokoto, H. & Uchida, Y. Interdependent Happiness: Theoretical Importance and Measurement Validity. *Journal of Happiness Studies* **16,** 211–239 (2015).

47. Adams, G. The Cultural Grounding of Personal Relationship: Enemyship in North American and West African Worlds. *Journal of personality and social psychology* **88.** Publisher: American Psychological Association, 948 (2005).

48. Nord, M., Cafiero, C. & Viviani, S. Methods for Estimating Comparable Prevalence Rates of Food Insecurity Experienced by Adults in 147 Countries and Areas. *Journal of Physics: Conference Series.* `https://doi.org/10.1088/1742-6596/772/1/012060` (2016).

49. Ballard, T. J., Kepple, A. W. & Cafiero, C. The Food Insecurity Experience Scale: Development of a Global Standard for Monitoring Hunger Worldwide. *FAO, Rome.* `http://www.fao.org/fileadmin/templates/ess/voh/FIES_Technical_Paper_v1.1.pdf` (2013).

50. WFP. *Food Consumption Analysis: Calculation and Use of the Food Consumption Score in Food Security Analysis* 2008.

51. Njuki, J., Poole, E. J., Johnson, J., Baltenweck, I., Pali, P. N., Lokman, Z. & Mburu, S. *Gender, livestock and livelihood indicators* 2011.

52. Radloff, L. S. The Ces-D Scale: A Self-Report Depression Scale for Research in the General Population. *Applied psychological measurement* **1,** 385–401 (1977).

53. Harding, T. W., De Arango, V., Baltazar, J., Climent, C., Ibrahim, H., Ladrido-Ignacio, L. & Wig, N. Mental Disorders in Primary Health Care: A Study of Their Frequency and Diagnosis in Four Developing Countries. *Psychological medicine* **10,** 231–241 (1980).

54. Cantril, H. *et al. Pattern of human concerns* (Rutgers University Press, 1965).

55. Deaton, A. Income, health, and well-being around the world: Evidence from the Gallup World Poll. *Journal of Economic perspectives* **22,** 53–72 (2008).

56. Schwarzer, R., Jerusalem, M., *et al.* Generalized Self-Efficacy Scale. *Measures in health psychology: A user's portfolio. Causal and control beliefs* **1,** 35–37 (1995).

57. Rosenberg, M. *Society and the Adolescent Self-Image* (Princeton University Press, 2015).

58. Singelis, T. M. The measurement of independent and interdependent self-construals. *Personality and social psychology bulletin* **20,** 580–591 (1994).

59. Triandis, H. C. & Gelfand, M. J. Converging measurement of horizontal and vertical individualism and collectivism. *Journal of personality and social psychology* **74,** 118 (1998).

60. Benjamini, Y. & Hochberg, Y. Controlling the False Discovery Rate: A Practical and Powerful Approach to Multiple Testing. *Journal of the Royal statistical society: series B (Methodological)* **57,** 289–300 (1995).

61. Barsbai, T., Licuanan, V., Steinmayr, A., Tiongson, E. & Yang, D. Information and the Acquisition of Social Network Connections. *NBER Working Paper No. 27346* (2020).

62. Baron, E. C., Davies, T. & Lund, C. Validation of the 10-item centre for epidemiological studies depression scale (CES-D-10) in Zulu, Xhosa and Afrikaans populations in South Africa. *BMC psychiatry* **17,** 1–14 (2017).

63. Romero, J., Esopo, K., McGuire, J. & Haushofer, J. The Effect of Economic Transfers on Psychological Well-Being and Mental Health. *Working Paper.* `https://haushofer.ne.su.se/publications/Romero_et_al_2021.pdf` (2021).
