# Supplementary material for: Tackling psychosocial and capital constraints to alleviate poverty
Source: Nature. 2022 Apr 27;605(7909):291–7. doi: 10.1038/s41586-022-04647-8 (PMC9095470; doi:10.1038/s41586-022-04647-8)
Supplement: Supplementary file 2 — Reporting Summary [file 41586_2022_4647_MOESM2_ESM.pdf]

nature portfolio

Corresponding author(s): Dean Karlan and Patrick Premand

Last updated by author(s): Feb 14, 2022

Reporting Summary

Nature Portfolio wishes to improve the reproducibility of the work that we publish. This form provides structure for consistency and transparency in reporting. For further information on Nature Portfolio policies, see our Editorial Policies and the Editorial Policy Checklist.

Statistics

For all statistical analyses, confirm that the following items are present in the figure legend, table legend, main text, or Methods section.

n/a	Confirmed	
☐	☒	The exact sample size (*n*) for each experimental group/condition, given as a discrete number and unit of measurement
☐	☒	A statement on whether measurements were taken from distinct samples or whether the same sample was measured repeatedly
☐	☒	The statistical test(s) used AND whether they are one- or two-sided<br>*Only common tests should be described solely by name; describe more complex techniques in the Methods section.*
☐	☒	A description of all covariates tested
☐	☒	A description of any assumptions or corrections, such as tests of normality and adjustment for multiple comparisons
☐	☒	A full description of the statistical parameters including central tendency (e.g. means) or other basic estimates (e.g. regression coefficient) AND variation (e.g. standard deviation) or associated estimates of uncertainty (e.g. confidence intervals)
☐	☒	For null hypothesis testing, the test statistic (e.g. *F*, *t*, *r*) with confidence intervals, effect sizes, degrees of freedom and *P* value noted<br>*Give P values as exact values whenever suitable.*
☒	☐	For Bayesian analysis, information on the choice of priors and Markov chain Monte Carlo settings
☒	☐	For hierarchical and complex designs, identification of the appropriate level for tests and full reporting of outcomes
☒	☐	Estimates of effect sizes (e.g. Cohen's *d*, Pearson's *r*), indicating how they were calculated

Our web collection on statistics for biologists contains articles on many of the points above.

Software and code

Policy information about availability of computer code

Data collection	Data used in this study were collected using Android tablets and the SurveyCTO Platform developed by Dobility, Inc, versions 2.0 - 2.6.
Data analysis	Data in this study was analyzed using the Stata software package (v15) developed by StataCorp.

For manuscripts utilizing custom algorithms or software that are central to the research but not yet described in published literature, software must be made available to editors and reviewers. We strongly encourage code deposition in a community repository (e.g. GitHub). See the Nature Portfolio guidelines for submitting code & software for further information.

Data

Policy information about availability of data

All manuscripts must include a data availability statement. This statement should provide the following information, where applicable:

- Accession codes, unique identifiers, or web links for publicly available datasets
- A description of any restrictions on data availability
- For clinical datasets or third party data, please ensure that the statement adheres to our policy

The code needed to reproduce the results is available at: https://github.com/dime-worldbank/niger-asp-reprod.git. The data used in this paper is available at: https://microdata.worldbank.org/index.php/catalog/4294.

Field-specific reporting

Please select the one below that is the best fit for your research. If you are not sure, read the appropriate sections before making your selection.

☐ Life sciences ☒ Behavioural & social sciences ☐ Ecological, evolutionary & environmental sciences

For a reference copy of the document with all sections, see nature.com/documents/nr-reporting-summary-flat.pdf

Behavioural & social sciences study design

All studies must disclose on these points even when the disclosure is negative.

Study description	We conduct a quantitative randomized evaluation among beneficiaries of a national cash transfer government program in Niger.
Research sample	The study sample consists of poor households already chosen to receive cash transfers from the government of Niger. Within the household, the nominal beneficiary is a woman over the age of 20.
Sampling strategy	Three alternative targeting methods were previously tested and randomized at the village level in the sample used for this study, including proxy means testing, community-based targeting, and a formula to proxy temporary food insecurity (described in Premand and Schnitzer, 2021). Out of the 22,507 cash transfer beneficiaries that were assigned to the 4 treatment variants described in the paper, 4,712 households were drawn into a sample for data collection (1206 households in control, 1191 households in capital, 1112 households in psychosocial and 1203 households in full). Before the study, we conducted power calculations assuming an ICC of 0.10 (based on data from Ghana and a Niger national household survey) and equal sized arms. To maximize power, we sampled all villages in this phase. Sampling 15 households per village allowed for minimum detectable sizes of 0.057 SD between arms, before adjusting for baseline outcomes or strata.
Data collection	Survey teams, blind to treatment status, were assigned to villages; but participants could indirectly reveal their treatment status to the enumerator in the last module of the midline survey. During the fieldwork, a remote team checked and updated the field plan for treatment balance across teams and survey weeks.
Timing	Baseline data collection took place between April and June 2017. The public lotteries took place after data collection in June 2017. Two follow-up surveys were collected. The midline occurred in February and March 2019, a median of 6 months (3 to 9 months) post-intervention. The endline survey occurred a year later in February and March 2020, a median of 18 months post-intervention.
Data exclusions	No surveyed households were excluded from the analysis.<br><br>We winsorize rather than trim continuous variables with observations above the 98th percentile. Where a component variable is missing, the aggregate variable itself is set to missing, except in harvest and consumption values.<br><br>Where an uninterpretable crop or food consumption entry If a crop or food represents less than 10% of the harvest or consumption value for at least 90% of the households, the crop or food component value is set to 0. If it represents more than 10% of the consumption or harvest value, the total harvest or consumption value is set to missing.
Non-participation	At midline and endline, 95.0% and 91.3% of baseline households were successfully interviewed, respectively. The remainders could not be located. Attrition was balanced across the treatment arms.
Randomization	The study is a cluster-randomized controlled trial in which villages with existing cash transfer beneficiaries were randomly allocated to one of four treatment arms. Within each village there was no additional randomization across households, and thus all eligible households within each village received the same treatment. Randomization of the villages was stratified by commune (17 overall) and the targeting method used to select cash transfer beneficiaries in each village (which is part of a complementary study) and took place in public lotteries. Specifically, to promote the transparency gained from public lotteries while maintaining balance across targeting methods, we proceeded in two stages. First, for each commune we randomly assigned villages into four lists stratified by targeting method. (The strata were based on a categorical variable with four values, one for each of three randomized targeting methods and a fourth for not being part of the targeting study). This stage did not assign the experimental arm label to each list. Second, we organized a public lottery in each of the 17 communes to randomly assign each list to one of the four experimental arms. The lottery was organized by the cash transfer program government team and held in the capital of the commune in the presence of village chiefs or elders.

Reporting for specific materials, systems and methods

We require information from authors about some types of materials, experimental systems and methods used in many studies. Here, indicate whether each material, system or method listed is relevant to your study. If you are not sure if a list item applies to your research, read the appropriate section before selecting a response.

Materials & experimental systems

n/a	Involved in the study	
☒	☐	Antibodies
☒	☐	Eukaryotic cell lines
☒	☐	Palaeontology and archaeology
☒	☐	Animals and other organisms
☐	☒	Human research participants
☒	☐	Clinical data
☒	☐	Dual use research of concern

Methods

n/a	Involved in the study	
☒	☐	ChIP-seq
☒	☐	Flow cytometry
☒	☐	MRI-based neuroimaging

Human research participants

Policy information about studies involving human research participants

Population characteristics	See above.
Recruitment	See above.
Ethics oversight	The research protocol was approved by Innovations for Poverty Action Institutional Review Board and preregistered in the AEA RCT Registry (study 0002544). The pre-analysis plan is registered at https://www.socialscienceregistry.org/versions/52534/docs/version/document. All survey participants completed informed consent. They were not compensated for their time as they were all part of the national cash transfer program.

Note that full information on the approval of the study protocol must also be provided in the manuscript.
